# Magnetic nanocatalyst for microwave-assisted synthesis of Benzo[4,5]imidazo[1,2-*a*]pyrimidines via A3 coupling

**DOI:** 10.3389/fchem.2025.1631183

**Published:** 2025-07-14

**Authors:** Yuqiang Pan

**Affiliations:** Guangxi Guida Agricultural Technology Co., Ltd., Nanning, Guangxi, China

**Keywords:** NiFe2O4@MCM-41@IL/pt, A3 coupling reactions, nanomagnetic catalyst, microwave-assisted, imidazo[1,2-a]pyrimidines

## Abstract

This manuscript introduces an innovative and environmentally benign magnetic nanocatalyst (NiFe_2_O_4_@MCM-41@IL/Pt(II)) designed to synthesize benzoimidazo[1,2-*a*]pyrimidines via a microwave-assisted, one-pot A3 coupling reaction. The methodology employs aromatic and heteroaromatic aldehydes, 2-aminobenzimidazole derivatives, and terminal alkynes in water as a green solvent, leveraging the synergistic effects of nanocatalysis and microwave irradiation. The magnetic nanocatalyst, characterized by its robust structure and high surface reactivity, facilitates rapid reaction kinetics, achieving excellent yields while significantly reducing energy consumption and reaction time compared to conventional thermal approaches. Its inherent magnetic properties enable straightforward separation and reuse across multiple cycles without appreciable loss in catalytic efficiency, aligning with sustainable chemistry principles. The protocol demonstrates broad substrate compatibility, successfully accommodating diverse aldehydes, including challenging heteroaromatic systems, to furnish a library of pharmaceutically relevant heterocycles. This broad substrate compatibility underscores the versatility of the nanocatalyst, making it a valuable tool for a wide range of chemical synthesis applications. This work highlights the transformative role of hybrid methodologies in addressing both efficiency and environmental impact in chemical synthesis.

## Graphical Abstract

**Figure:**
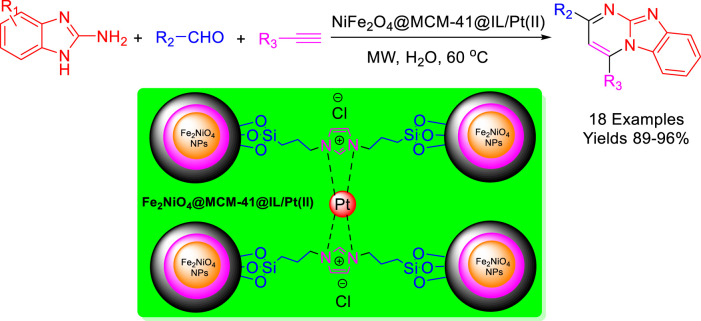

## 1 Introduction

The field of organic synthesis is increasingly embracing greener and more efficient methodologies, primarily driven by green chemistry principles. This shift aims to minimize hazardous substances and waste, utilize sustainable resources, and improve the efficiency of chemical reactions (Rotstein et al., 2014; Javahershenas and Nikzat, 2023; Zhu et al., 2015). Among the innovative strategies emerging, one-pot multicomponent reactions (MCRs) stand out as a powerful approach in organic synthesis, inspiring new possibilities. MCRs enable the simultaneous condensation of multiple reagents to create complex structures while reducing the formation of by-products (Georg Thieme Verlag KG, 2014; Javahershenas et al., 2024a).

**FIGURE 1:**
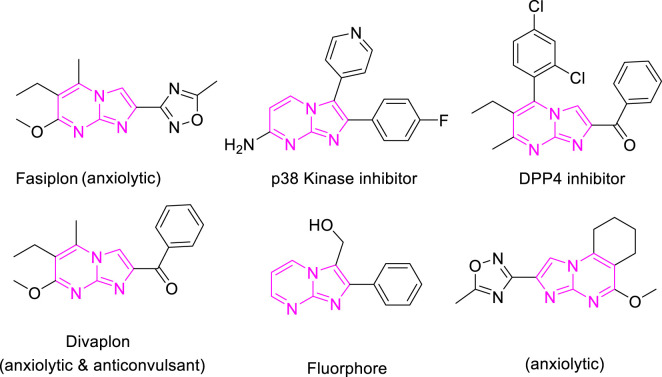
Biologically potent imidazo[1,2-*a*]pyrimidines.

Imidazo[1,2-a]pyrimidines are a notable class of heterocyclic compounds that have attracted attention for their diverse biological activities and potential applications in medicinal chemistry and materials science (Berson et al., 2001; Harrison and Keating, 2005; Hanson et al., 2008; Monti et al., 2009; Enguehard-Gueiffier and Gueiffier, 2007; Denora et al., 2008; Trapani et al., 2005). These fused ring systems exhibit various pharmacological properties, including antibacterial (Rival et al., 1992), anticancer (Panda et al., 2022), antimicrobial (Revankar et al., 1975), and antifungal (Rival et al., 1991) activities. Traditional synthetic routes to these valuable scaffolds often involve multi-step procedures that require harsh reaction conditions and environmentally harmful reagents. Therefore, there is a pressing need for more sustainable and efficient methods to synthesize these important molecular frameworks, underscoring the significance of our work in the field (Figure 1) (Tully et al., 1991; Rupert et al., 2003; Feely et al., 1989; O’Connor et al., 2010; Linton et al., 2011).

Microwave-assisted organic synthesis (MAOS) stands out as a potent technique, accelerating chemical reactions with its unique advantages. It offers enhanced reaction rates, higher yields, and superior selectivity compared to conventional heating methods. By employing microwave irradiation, MAOS facilitates chemical reactions, resulting in increased reaction rates, reduced energy consumption, and improved yields (Gulati et al., 2021; Javahershenas et al., 2024b). The technique’s ability to interact uniquely with polar reactants and solvents, promoting uniform heating and accelerating reaction kinetics, is a game-changer. The underlying mechanism of microwave-assisted synthesis involves rapid and uniform heating of reaction mixtures, which enhances molecular interactions under mild conditions. In green chemistry, microwave-assisted reactions are particularly advantageous as they reduce energy consumption and minimize reaction times, making them ideal for scalable industrial processes. The ability to perform reactions efficiently within short timeframes positions microwave-assisted synthesis as a valuable tool for producing complex organic molecules (Kappe and Dall’Acqua, 2019; Zhang and Cai, 2020; Kappe and Stadler, 2006).

The role of nanotechnologies, particularly nanocatalysis, in revolutionizing the optimization of chemical reactions cannot be overstated. Developing efficient and reusable catalysts is essential for achieving sustainable chemical processes. Nanocatalysts, such as NiFe_2_O_4_, offer distinct advantages, including high catalytic activity, stability, and ease of separation from reaction mixtures through magnetic decantation (Amrutkar et al., 2022; Kanithan et al., 2022; Chandra, 2021). This capability significantly reduces waste and enhances catalyst recovery, aligning with one of the core principles of green chemistry—minimizing waste. When combined with mesoporous materials like MCM-41, nanocatalysts can form an efficient catalyst system, enhancing reaction rates and product selectivity (Kazemi, 2020; Wang et al., 2021).

Integrating mesoporous materials, like MCM-41, with metal oxides creates a synergistic effect that enhances the dispersion and accessibility of active catalytic sites. Mesoporous materials’ high surface area and favorable pore structure are essential for accommodating reactants and facilitating catalytic activity (Snoussi et al., 2018; Kefayati et al., 2016). Furthermore, when combined with ionic liquids (ILs), known for their low volatility and high thermal stability, these composites exhibit enhanced efficiency and selectivity, often surpassing traditional catalytic systems. Ionic liquids, composed of organic cations and inorganic anions, possess unique properties such as negligible vapor pressure and tunable solubility, making them versatile in catalysis and green chemistry. In heterogeneous catalysis, ILs can function as both reaction media and catalyst modifiers, boosting the stability and activity of supported metal species (Sead et al., 2025e; Zhu et al., 2010; Javahershenas et al., 2025).

By merging the magnetic properties of NiFe_2_O_4_ with the mesoporosity of MCM-41 and the solvation capabilities of ionic liquids, novel catalytic systems such as the NiFe_2_O_4_@MCM-41@IL/Pt(II) complex can be developed. This multi-faceted approach maximizes the benefits of each component, leading to a robust and versatile catalytic framework (Rogers and Seddon, 2002; Parvulescu and Hardacre, 2007; ; Saha et al., 2009; Zare et al., 2009; Hasaninejad et al., 2010).

The synthesis of fused imidazo[1,2-*a*]pyrimidines via A3 coupling reactions is a remarkable advancement in one-pot multicomponent reactions (MCRs). The A3 coupling process, which integrates an aldehyde, an alkyne, and an amine, showcases the efficiency of atom-economical methodologies. These methodologies allow for the construction of complex molecular architectures in a single synthetic step, a feat that was previously challenging to achieve (Javahershenas and Mole, 2023; Khan et al., 2019; Zheng et al., 2020).

Utilizing the NiFe_2_O_4_@MCM-41@IL/Pt (II) complex as a catalyst within this MCR framework can significantly improve the reaction’s efficiency and sustainability. The incorporation of platinum (Pt) further enhances the catalytic properties of this nanocomposite. Platinum is well-known for its effectiveness as a catalyst in cross-coupling reactions, including A3 coupling, where it facilitates the condensation of an aldehyde, an amine, and an alkyne to form intricately fused imidazo[1,2-*a*]pyridine frameworks. The appeal of the A3 coupling reaction lies in its operational simplicity and minimal waste production (Pourhasan-Kisomi et al., 2018; Kazemi and Ghobadi, 2017; Kazemi, 2023; Wang et al., 2021; Abedi et al., 2021; Huang et al., 2021; Sead et al., 2025a).

However, challenges such as Pt leaching and catalyst recovery have highlighted the need for robust catalytic systems that effectively address these issues. Developing a stable and recyclable catalyst is crucial for optimizing the sustainability of the A3 coupling reaction while maintaining high catalytic activity (Sead et al., 2025b; Sead et al., 2025c; Sead F. F. et al., 2025).

In this study, we introduce the synthesis and application of a promising microwave-assisted NiFe_2_O_4_@MCM-41@IL/Pt(II) complex nanomagnetic catalyst for the one-pot green synthesis of fused imidazo[1,2-a]pyrimidines through A3 coupling reactions. The hybrid catalyst, meticulously crafted by combining NiFe_2_O_4_ nanoparticles with MCM-41 and then functionalizing with ionic liquid components and platinum, demonstrates superior catalytic performance, high recyclability, and environmental compatibility. This catalyst holds great promise for advancing sustainable chemistry principles in the field of organic synthesis.

The primary objective of this research is to investigate the efficiency and reusability of the NiFe_2_O_4_@MCM-41@IL/Pt (II) catalyst in various A3 coupling reactions under microwave irradiation. The rapid reaction times and enhanced yields achieved using this system highlight the potential of microwave-assisted synthesis for the efficient construction of valuable fused imidazo[1,2-*a*]pyridine derivatives. Additionally, the catalyst’s magnetic properties facilitate easy recovery from reaction mixtures, significantly simplifying the purification process and enhancing the sustainability of the overall synthetic pathway.

Understanding the A3 coupling reaction’s mechanistic aspects is crucial. It provides insights into each component’s contributions within the catalyst system. This understanding is key to how the synergistic interactions among NiFe_2_O_4_, MCM-41, ionic liquids, and platinum enhance catalytic performance. It will pave the way for designing even more advanced catalytic systems.

In summary, developing the NiFe_2_O_4_@MCM-41@IL/Pt (II) complex nanomagnetic catalyst significantly advances sustainable organic synthesis. By leveraging the advantages of microwave-assisted synthesis, nanocatalysis, and the unique properties of ionic liquids, this work offers a robust and efficient method for synthesizing fused imidazo[1,2-a]pyrimidines. The implications of this research extend beyond immediate synthetic applications, potentially influencing a broader range of catalytic processes and initiatives aimed at environmental sustainability in organic chemistry.

## 2 Result and discussion

**SCHEME 1:**
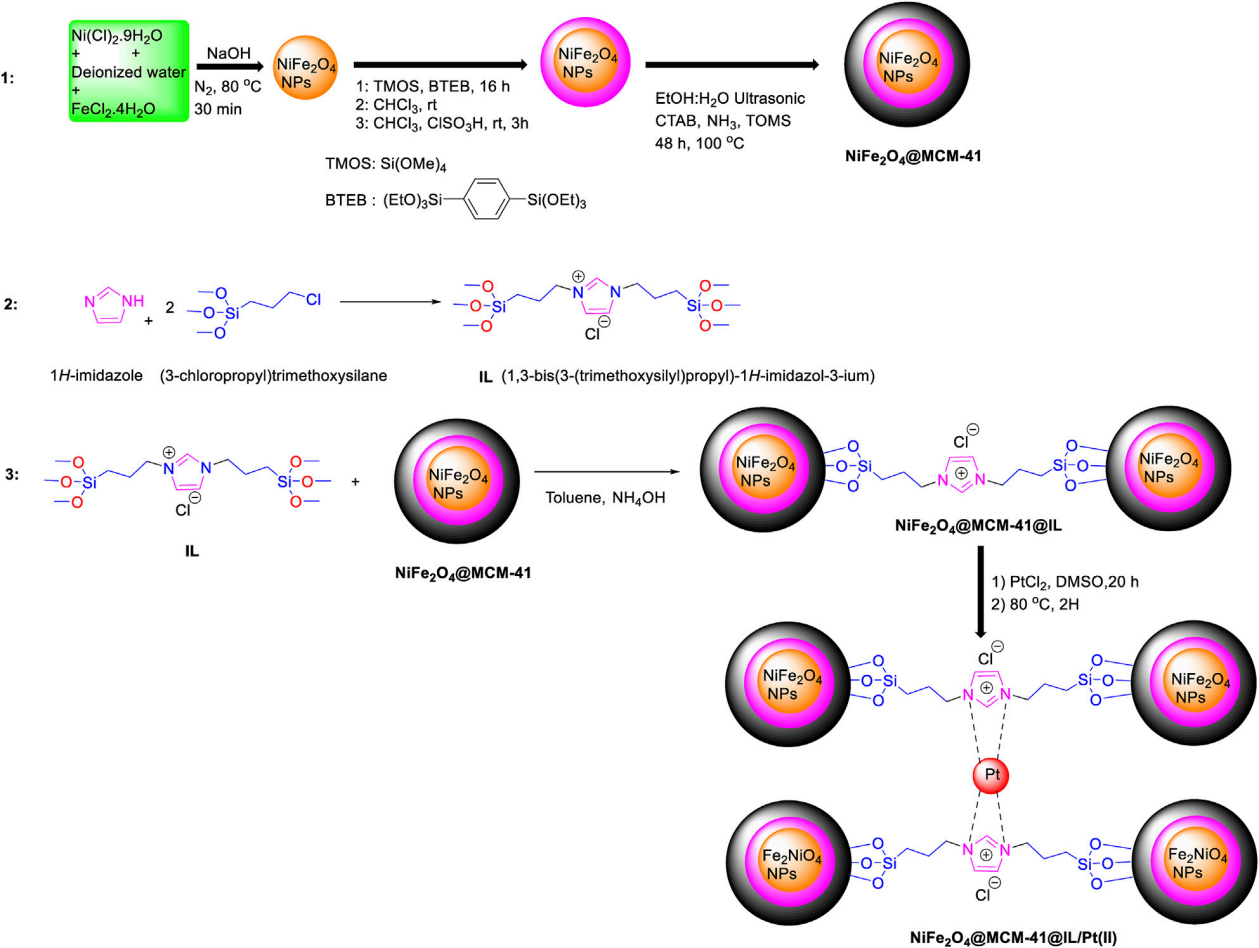
Preparation of NiFe_2_O_4_@MCM41@IL/Pt (II) nanocatalyst.

Scheme 1 outlined the synthetic route for preparing a novel nanocatalyst, NiFe_2_O_4_@MCM-41@IL/Pt. Nickel chloride hexahydrate (NiCl_2_·9H_2_O) and iron chloride tetrahydrate (FeCl_2_·4H_2_O) were dissolved in deionized water. Sodium hydroxide (NaOH) was added to the solution, and the mixture was heated at 80°C for 30 min, leading to the formation of NiFe_2_O_4_ nanoparticles.

**SCHEME 2:**
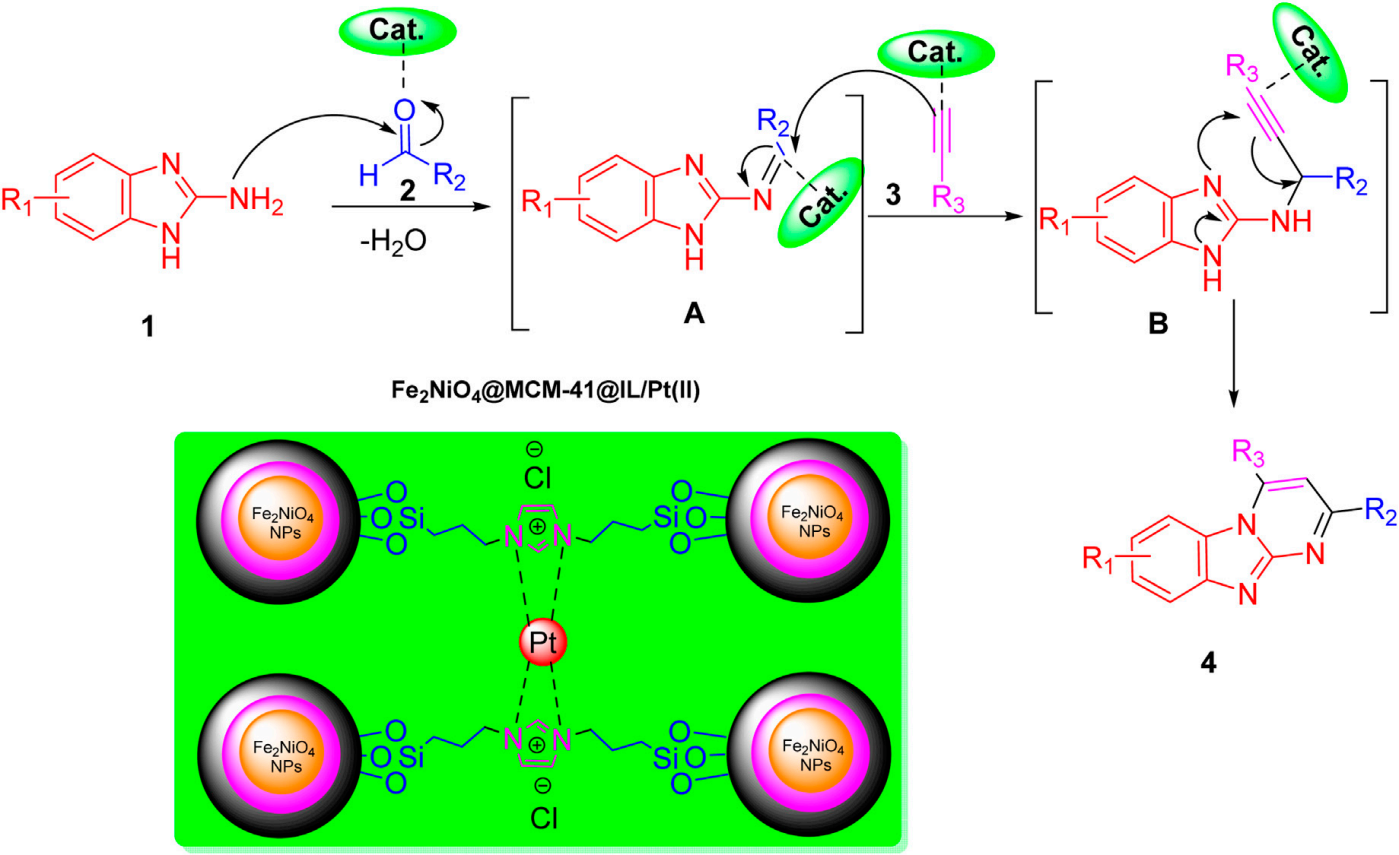
A plausible mechanism for the synthesis of imidazo[1,2-a]pyrimidines derivatives catalyzed by NiFe_2_O_4_@MCM‐41@IL/Pt nanocomposite.

For the functionalization of SiO_2_ with organic groups, tetramethoxysilane (TMOS) and bis (triethoxysilyl) benzene (BTEB) were added to the NiFe_2_O_4_ nanoparticles. The mixture was stirred for 16 h. Subsequently, chlorosulfonic acid (ClSO_3_H) was introduced, and the mixture was stirred in chloroform (CHCl_3_) for 3 h.

The functionalized MCM-41 was mixed with a solution containing NiFe_2_O_4_ nanoparticle nanoparticles, cetyltrimethylammonium bromide (CTAB), ammonia (NH_3_), and toluene. The mixture was sonicated for 48 h at 100°C, resulting in the attachment of NiFe_2_O_4_ nanoparticles to the MCM-41 support via the organic functional groups.

For immobilization of NiFe_2_O_4_@MCM-41 nanoparticles onto the ionic liquid (IL), (3-chloropropyl)trimethoxysilane was reacted with 1H-imidazole to produce IL (1,3-bis(3-(trimethoxysilyl)propyl)-1H-imidazol-3-ium). The mixture of NiFe_2_O_4_@MCM-41 with this IL was then added to a dispersion of toluene and NH_4_OH and stirred for 24 h.

The final nanocatalyst, NiFe_2_O_4_@MCM-41@IL, was dispersed in a solution containing PtCl_2_ and DMSO. The mixture was stirred for 20 h at room temperature, then heated at 80°C for 2 h. The product was filtered and washed with ethanol to obtain the final NiFe_2_O_4_@MCM-41@IL/Pt nanocatalyst.

This nanocatalyst was expected to exhibit unique catalytic properties owing to the synergistic effects of NiFe_2_O_4_ nanoparticles, the MCM-41 support, the ionic liquid, and platinum nanoparticles.

**FIGURE 2:**
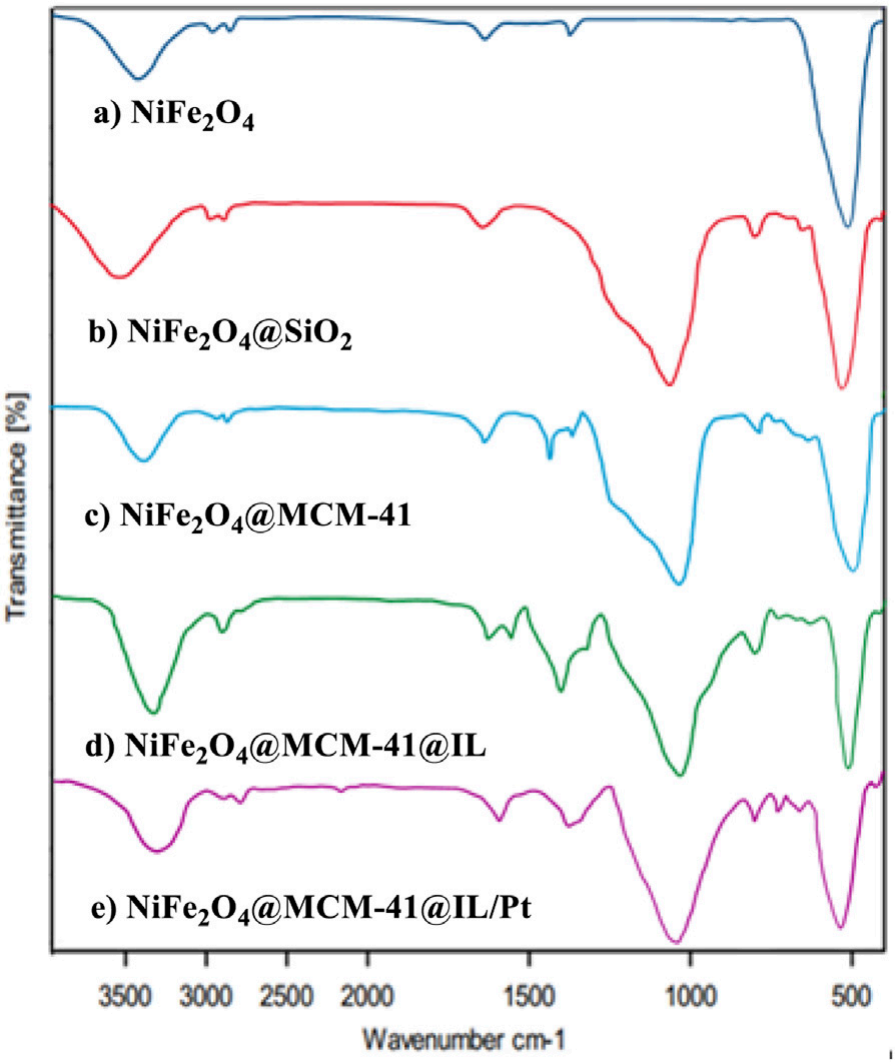
FT-IR spectrums of NiFe_2_O_4_ NPs, NiFe_2_O_4_@SiO_2_, NiFe_2_O_4_@MCM‐41, NiFe_2_O_4_@MCM‐41@IL, and NiFe_2_O_4_@MCM‐41@IL/Pt (II) nanocomposites.

This study presents a comprehensive analysis of the Fourier-transform infrared (FT-IR) spectra of various nickel-iron oxide nanocomposites, as illustrated in Figure 2. The spectra encompass NiFe_2_O_4_ nanoparticles (NPs) and their functionalized derivatives, including NiFe_2_O_4_@SiO_2_, NiFe_2_O_4_@MCM-41, NiFe_2_O_4_@MCM-41@IL, and NiFe_2_O_4_@MCM-41@IL/Pt(II). Each spectrum provides insights into the structural and functional characteristics of these materials.

The spectrum for pure NiFe_2_O_4_ nanoparticle NPs (curve a) displays characteristic absorption bands corresponding to the metal-oxygen vibrations typical of spinel ferrites. Notably, the peaks around 580 cm^-1^ indicate the Fe-O bonds in the octahedral sites, while those near 400 cm^-1^ correspond to the tetrahedral sites. These features confirm the successful synthesis of NiFe_2_O_4_ with its expected crystalline structure.

Additional peaks emerge that signify Si-O stretching vibrations upon functionalization with silica in curve b (NiFe_2_O_4_@SiO_2_). This indicates successful incorporation of silica onto the nickel iron oxide framework, enhancing its stability and inspiring potential applications in catalysis and adsorption processes.

The spectrum for NiFe_2_O_4_@MCM-41 (curve c) reveals further modifications. The presence of MCM-41, a mesoporous silica material, is evidenced by distinct absorption bands associated with silanol groups and the characteristic pore structure of MCM-41. This modification increases surface area and facilitates enhanced interaction with reactants in catalytic applications.

In curve d, representing NiFe_2_O_4_@MCM-41@IL, we observe additional peaks related to ionic liquid functionalities. Incorporating ionic liquids is significant as it can improve solubility and enhance catalytic activity by providing a unique environment for reaction processes. This modification suggests a tailored approach to optimize catalytic performance through solvent effects.

Finally, curve e illustrates the FT-IR spectrum of NiFe_2_O_4_@MCM-41@IL/Pt (II), introducing platinum species into the composite. The presence of Pt(II) is confirmed by new absorption bands that emerge in this spectrum, indicating successful loading of platinum onto the nanocomposite. This addition is crucial as platinum is known for its exceptional catalytic properties, particularly in hydrogenation reactions and fuel cells.

Comparative analysis of these spectra reveals significant insights into how each modification impacts the structural integrity and functionality of the nickel-iron oxide nanocomposites. The progressive introduction of silica, mesoporous structures, ionic liquids, and platinum enhances not only the physical properties but also expands their applicability across various fields, such as catalysis and environmental remediation.

This FT-IR analysis highlights the importance of each component within these nanocomposites and underscores how systematic modifications, a crucial part of the research process, can lead to materials with tailored properties suitable for advanced applications. Such insights contribute to ongoing research to optimize catalyst design for improved efficiency and effectiveness in chemical processes.

**FIGURE 3:**
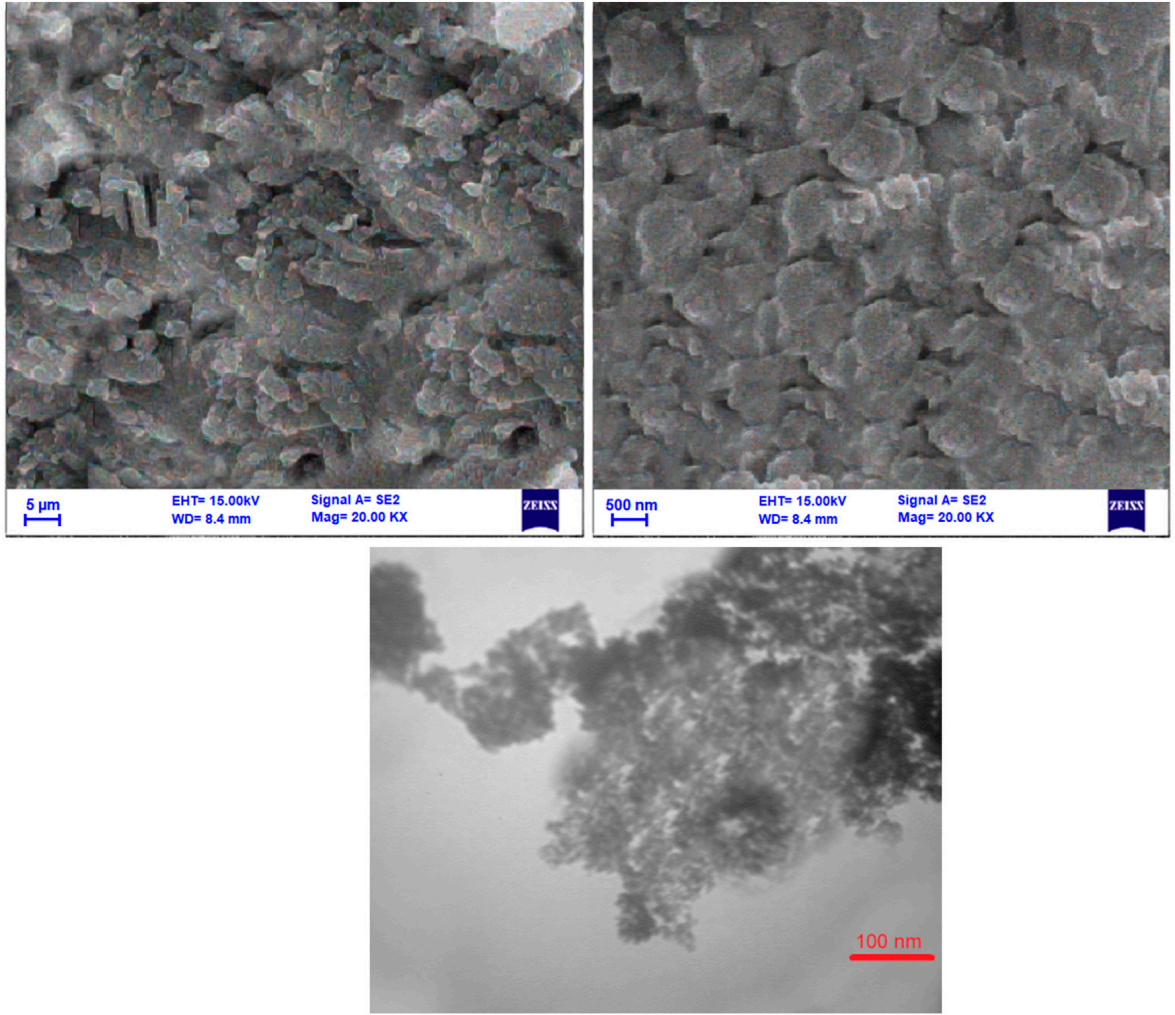
SEM and TEM images of NiFe_2_O_4_@MCM‐41@IL/Pt nanocatalyst at different magnifications.

Examining the morphology and structure of the catalyst is a critical aspect of contemporary catalysis research. In this regard, SEM and TEM analyses were used to investigate the morphology and shape of the NiFe_2_O_4_@MCM‐41@IL/Pt (II) catalyst particles in Figure 3. The SEM images reveal a porous, agglomerated structure with a rough surface. The higher magnification image (100 nm scale bar) shows a more detailed view of the individual particles, which appear spherical or slightly irregular. The TEM image provides a closer look at the nanocatalyst’s morphology. It shows a well-dispersed distribution of nanoparticles, with some agglomeration visible. The particles exhibit a spherical or slightly elongated shape with a relatively uniform size distribution.

Morphology: Both techniques confirm the presence of a porous structure with agglomerated particles. The TEM image offers a more detailed insight into the individual particle morphology, revealing their spherical or slightly elongated shape.

Particle Size Distribution: The TEM image suggests the nanoparticles’ relatively uniform size distribution, consistent with the SEM observations.

Dispersion: The TEM image shows a well-dispersed distribution of nanoparticles, indicating good dispersion within the support material.

The SEM and TEM images provide valuable information about the morphology and structure of the NiFe_2_O_4_@MCM-41@IL/Pt nanocatalyst. The porous structure observed in both images is likely beneficial for catalytic applications as it can provide a large surface area for reactant adsorption and product desorption. The uniform size distribution and good dispersion of the nanoparticles are also desirable features for catalytic activity.

**FIGURE 4:**
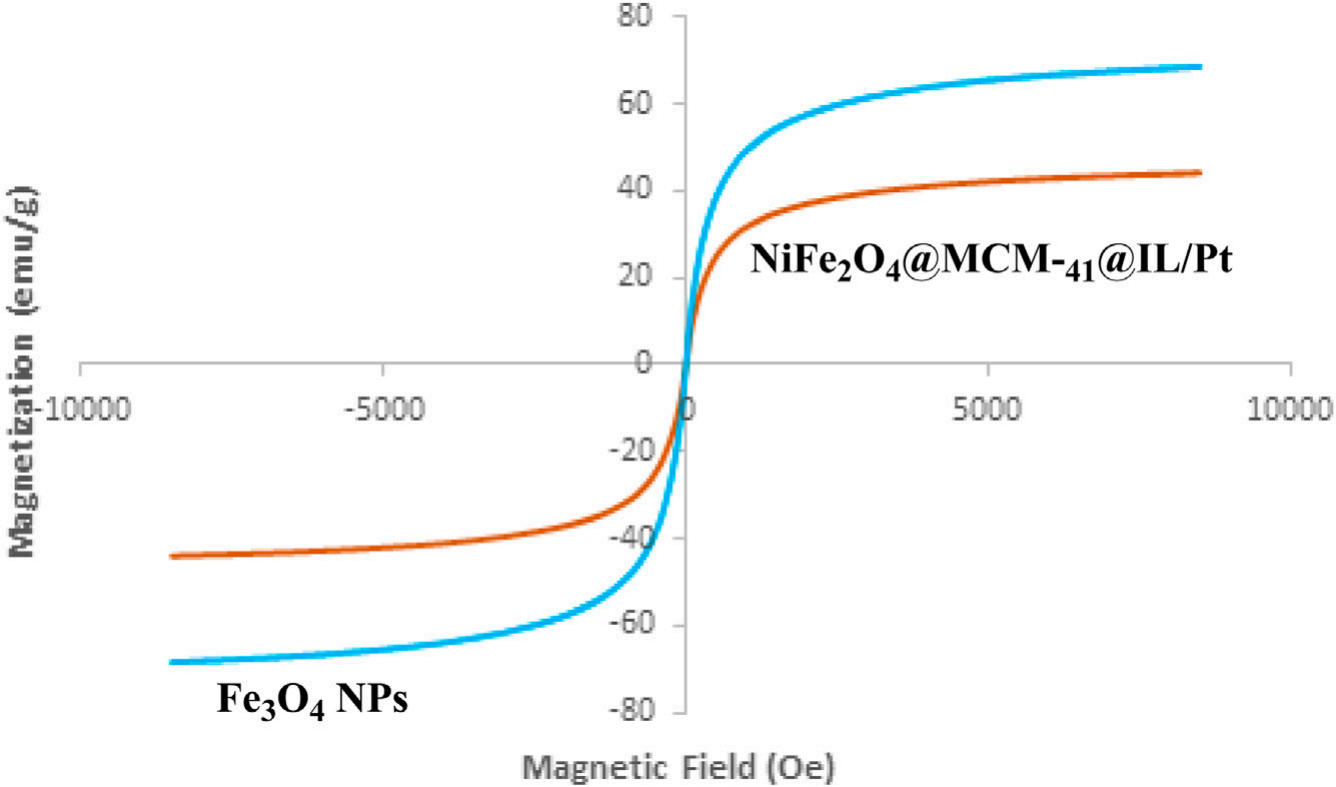
VSM analysis of Fe_3_O_4_ NPs and NiFe_2_O_4_@MCM‐41@IL/Pt nanocatalyst.

Figure 4 presents the magnetization curves obtained from Vibrating Sample Magnetometry (VSM) measurements for Fe_3_O_4_ NPs and NiFe_2_O_4_@MCM-41@IL/Pt nanocatalyst. The magnetization (M) is plotted against the applied magnetic field (H) in units of emu/g and Oe, respectively.

Saturation Magnetization: The saturation magnetization (Ms) is the maximum magnetization achieved when a material is subjected to a strong magnetic field. From the curves, it is evident that the NiFe_2_O_4_@MCM-41@IL/Pt nanocatalyst exhibits a significantly higher Ms compared to Fe_3_O_4_ NPs. This suggests that the NiFe_2_O_4_@MCM-41@IL/Pt nanocatalyst possesses a stronger magnetic response than the Fe_3_O_4_ NPs.

Hysteresis Loop: The hysteresis loop is the curve traced by the magnetization as the applied magnetic field increases and then decreases. The area enclosed by the hysteresis loop represents the energy loss during a magnetization cycle. The NiFe_2_O_4_@MCM-41@IL/Pt nanocatalyst exhibits a narrower hysteresis loop than Fe_3_O_4_ NPs, indicating a lower energy loss during magnetization reversal. This is beneficial for applications where energy efficiency is a concern.

Coercivity (Hc) is the magnetic field required to reduce the magnetization of a material to zero after it has been saturated. The NiFe_2_O_4_@MCM-41@IL/Pt nanocatalyst shows a lower Hc than Fe_3_O_4_ NPs. This suggests that the NiFe_2_O_4_@MCM-41@IL/Pt nanocatalyst is more straightforward to magnetize and demagnetize, which is desirable for applications requiring rapid magnetic switching. The enhanced magnetic properties of the NiFe_2_O_4_@MCM-41@IL/Pt nanocatalyst compared to Fe_3_O_4_ NPs can be attributed to several factors:

NiFe_2_O_4_ nanoparticles within the MCM-41 support matrix can contribute to an increase in Ms and a decrease in Hc. NiFe_2_O_4_ is known for its high magnetic moment and low coercivity. The MCM-41 support can provide a large surface area for the deposition of NiFe_2_O_4_ nanoparticles, leading to a higher concentration of magnetic material and, consequently, a higher Ms. The IL coating on the NiFe_2_O_4_@MCM-41 nanoparticles can further enhance the magnetic properties by improving the dispersion of the nanoparticles and reducing interparticle interactions. The presence of Pt nanoparticles on the surface of the NiFe_2_O_4_@MCM-41@IL nanoparticles can also influence the magnetic properties, although the exact mechanism is not fully understood.

The VSM results demonstrate that the NiFe_2_O_4_@MCM-41@IL/Pt nanocatalyst exhibits superior magnetic properties to Fe_3_O_4_ NPs. These enhanced properties make the NiFe_2_O_4_@MCM-41@IL/Pt nanocatalyst a promising material for various applications, including magnetic separation, catalysis, and drug delivery.

**FIGURE 5:**
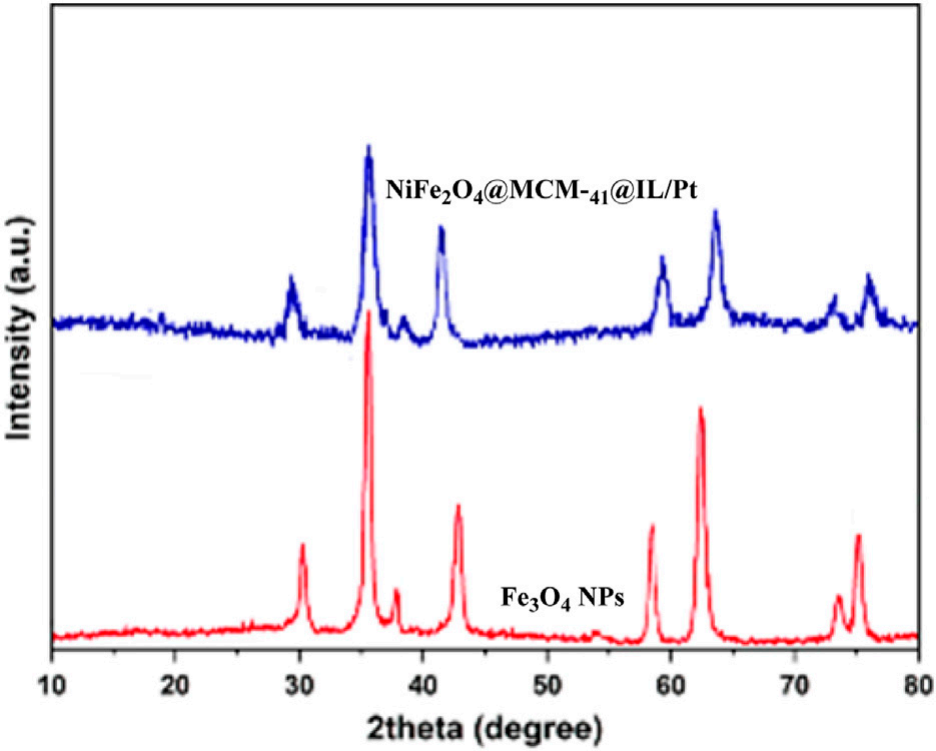
XRD patterns of Fe_3_O_4_ NPs and NiFe_2_O_4_@MCM‐41@IL/Pt nanocatalyst.

X-ray diffraction (XRD) is a powerful technique for characterizing the crystalline structure of materials. In this study, XRD patterns were obtained for Fe_3_O_4_ nanoparticles (NPs) and v NiFe_2_O_4_@MCM-41@IL/Pt nanocatalyst in Figure 5. These patterns provide insights into the samples’ phase purity, crystallinity, and lattice parameters.

The XRD patterns of Fe_3_O_4_ NPs and NiFe_2_O_4_@MCM-41@IL/Pt nanocatalyst are presented in Figure 5. The XRD pattern of Fe_3_O_4_ NPs exhibits a series of sharp peaks, indicating a highly crystalline nature. The positions and relative intensities of these peaks match well with the standard XRD pattern of magnetite (Fe_3_O_4_), confirming the phase purity of the sample. The absence of additional peaks suggests minimal impurities in the sample. The XRD pattern of NiFe_2_O_4_@MCM-41@IL/Pt nanocatalyst shows a distinct difference compared to the Fe_3_O_4_ NPs. The peaks are broader and less intense, suggesting a lower degree of crystallinity. This is likely due to the MCM-41 support and the incorporation of IL and Pt nanoparticles, which can disrupt the long-range order of the crystal lattice.

Fe_3_O_4_ NPs exhibit higher crystallinity compared to the NiFe_2_O_4_@MCM-41@IL/Pt nanocatalyst. This is evident from the sharper and more intense peaks in the Fe_3_O_4_ pattern. Both samples appear phase pure, as no additional peaks corresponding to impurities are observed in either pattern. The positions of the peaks in the XRD patterns can be used to calculate the lattice parameters of the samples. However, this analysis is beyond the scope of this brief description.

XRD analysis confirms the successful synthesis of Fe_3_O_4_ NPs and NiFe_2_O_4_@MCM-41@IL/Pt nanocatalyst. The Fe_3_O_4_ NPs exhibit high crystallinity and phase purity. At the same time, the NiFe_2_O_4_@MCM-41@IL/Pt nanocatalyst shows a lower degree of crystallinity due to the MCM-41 support and incorporated IL and Pt nanoparticles.

**FIGURE 6:**
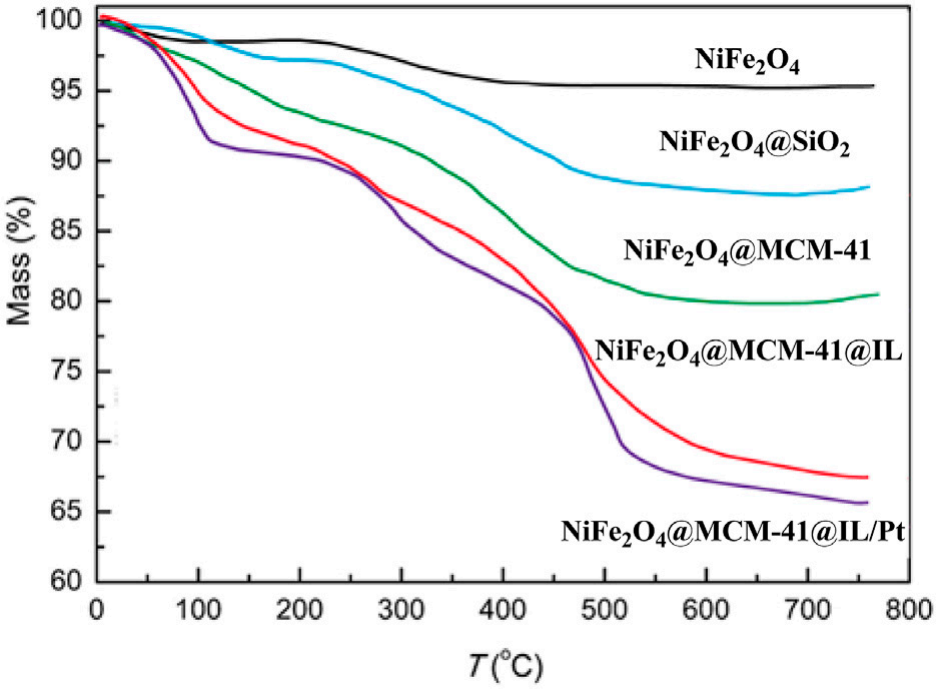
TGA spectrums of NiFe_2_O_4_@MCM‐41@IL/Pt nanocatalyst.

Figure 6 presents the thermogravimetric analysis (TGA) spectra of the NiFe_2_O_4_@MCM-41@IL/Pt nanocatalyst. The TGA curve reveals a multi-step weight loss pattern, indicating the presence of various components with different thermal stabilities.

Initial Weight Loss (Up to ∼200°C): A slight weight loss is observed in the initial temperature range, likely due to removing adsorbed water and volatile organic compounds. First Major Weight Loss (200°C–500°C): A significant weight loss occurs in this temperature range, which can be attributed to the decomposition of the ionic liquid (IL) component. The IL acts as a stabilizing agent and provides a confined environment for the growth of nanoparticles, but its thermal decomposition leads to a substantial mass loss. Second Major Weight Loss (500°C–700°C): A further weight loss in this temperature range can be associated with the decomposition of the organic template (MCM-41) used to synthesize the mesoporous support. The MCM-41 structure provides a high surface area for the dispersion of active nanoparticles, but its removal at higher temperatures results in additional mass loss. Final Weight Loss (Above 700°C): A minimal weight loss is observed at temperatures above 700°C, indicating the presence of a highly stable residual phase. This phase is likely composed of the NiFe_2_O_4_ nanoparticles and possibly residual Pt nanoparticles, which exhibit high thermal stability.

The TGA analysis confirms the presence of multiple components in the NiFe_2_O_4_@MCM-41@IL/Pt nanocatalyst. The multi-step weight loss pattern highlights the composite material’s complex nature, with each step corresponding to the thermal decomposition of specific components.

The TGA analysis provides valuable insights into the thermal stability and composition of the NiFe_2_O_4_@MCM-41@IL/Pt nanocatalyst. The multi-step weight loss pattern confirms the presence of various components, including the IL, mesoporous support, and active nanoparticles. Understanding the catalyst’s thermal behavior is crucial for optimizing its synthesis and application in various catalytic processes.

**FIGURE 7:**
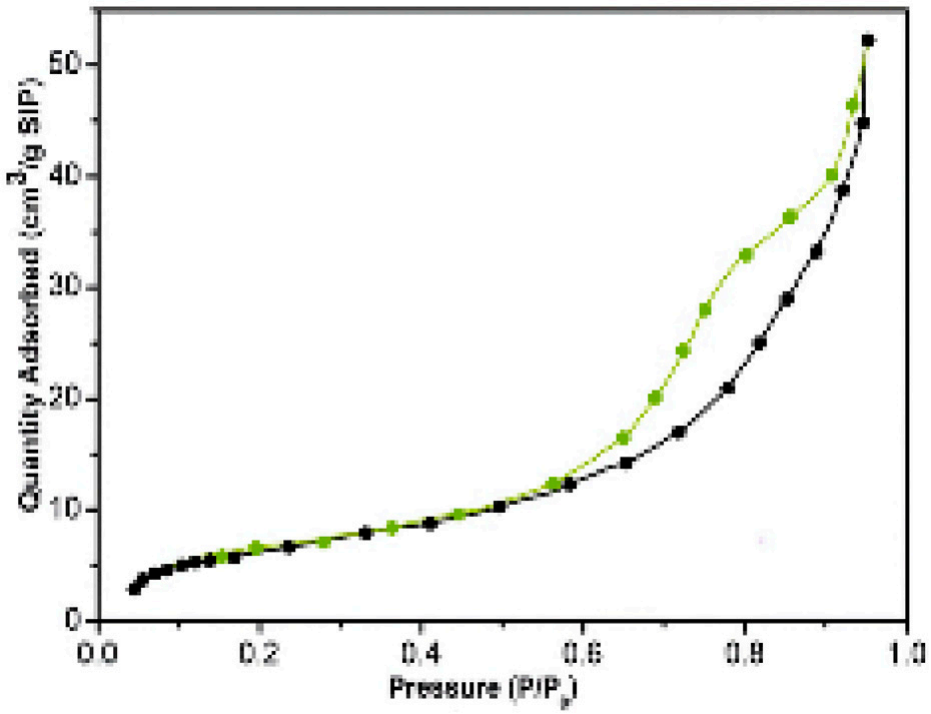
BET analysis of NiFe_2_O_4_@MCM‐41@IL/Pt nanocatalyst.

Figure 7 presents the BET analysis of the NiFe_2_O_4_@MCM-41@IL/Pt nanocatalyst. The BET (Brunauer-Emmett-Teller) analysis is a widely used technique to determine porous materials’ specific surface area, pore size distribution, and pore volume. In this case, the BET analysis provides insights into the nanocatalyst’s textural properties.

The adsorption isotherm in Figure 7 shows a Type IV curve, characteristic of mesoporous materials. This indicates that the nanocatalyst possesses a network of interconnected pores with a relatively uniform size distribution. The specific surface area of the nanocatalyst, as determined by the BET analysis, is expected to be high due to the presence of mesopores. A high surface area is crucial for catalytic applications, as it provides more active sites for reactant adsorption and interaction with the catalyst surface. The pore size distribution of the nanocatalyst can be estimated from the adsorption isotherm. The presence of mesopores in the nanocatalyst is beneficial for catalytic reactions, as they can facilitate the diffusion of reactants and products within the pores.

The NiFe_2_O_4_@MCM-41@IL/Pt nanocatalyst can be compared with the BET analysis of other materials, such as pristine NiFe_2_O_4_, MCM-41, and Pt nanoparticles. This comparison can help understand the different components’ impact on the nanocatalyst’s textural properties. As revealed by the BET analysis, the nanocatalyst’s textural properties can correlate with its catalytic performance in various reactions. A high surface area and well-developed pore structure can enhance the nanocatalytic activity and selectivity of the nanocatalyst.

In conclusion, the BET analysis provides valuable information about the textural properties of the NiFe_2_O_4_@MCM-41@IL/Pt nanocatalyst. The presence of mesopores with a high surface area is expected to contribute to this material’s excellent catalytic performance.

**FIGURE 8:**
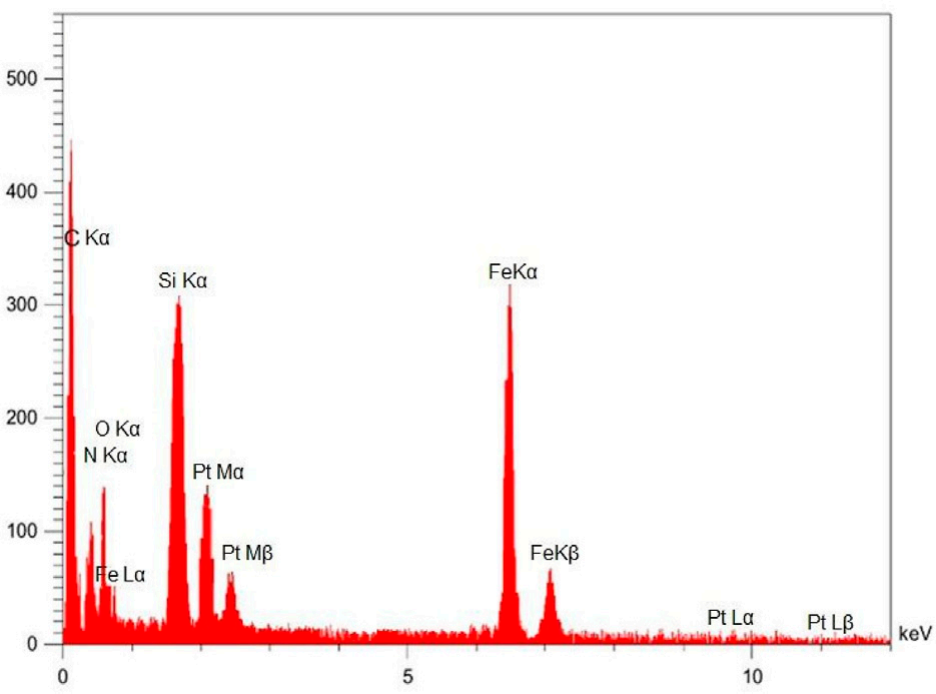
EDX analysis of NiFe_2_O_4_@MCM‐41@IL/Pt nanocatalyst.

Figure 8 presents the Energy Dispersive X-ray (EDX) spectrum obtained from the NiFe_2_O_4_@MCM-41@IL/Pt nanocatalyst, confirming the presence of the constituent elements. The spectrum displays distinct peaks corresponding to Nickel (Ni), Iron (Fe), Oxygen (O), Silicon (Si), and Platinum (Pt), which are consistent with the expected composition of the synthesized material. Notably, the presence of Pt peaks (Pt Mα, Pt Mβ, Pt Lα, and Pt Lβ) substantiates the successful incorporation of platinum onto the catalyst support. The peaks observed for Ni and Fe indicate the presence of the NiFe_2_O_4_ component, while the Si and O peaks are attributed to the MCM-41 support. Additionally, peaks corresponding to Carbon (C) and Nitrogen (N) are observed, likely originating from the ionic liquid (IL) component of the catalyst. The relative intensities of the peaks provide a semi-quantitative estimate of the elemental composition, suggesting a higher concentration of Si than the other metallic elements. This observation is consistent with the expected structure of the catalyst, where the NiFe_2_O_4_ nanoparticles are supported on the MCM-41 matrix and further functionalized with the ionic liquid and platinum.

The EDX spectrum is direct evidence for successfully synthesizing the intended composite material. The presence of peaks for all expected elements (Ni, Fe, O, Si, Pt, and likely C and N) confirms the successful incorporation of each component into the final catalyst structure.

While EDX is not ideal for precise quantification, the relative peak intensities offer insight into the relative abundance of elements. The higher intensity of Si peaks suggests that it is the most abundant element detected, which is reasonable considering that MCM-41 is the primary support material.

The clear presence of Pt peaks is crucial, as it confirms the successful loading of platinum onto the catalyst. The intensity and shape of the Pt peaks can potentially provide information about the platinum’s oxidation state and distribution, although more detailed analysis might be required for a deeper understanding.

The relatively strong Si and O signals, attributed to the MCM-41 support, are expected to be more intense than the signals from the NiFe_2_O_4_ and Pt components, which are present as nanoparticles or surface modifications.

C and N peaks suggest the successful incorporation of the ionic liquid. However, it is important to note that these elements are common and could originate from adventitious carbon or other sources.

EDX is a surface-sensitive technique and may not accurately represent the bulk composition. Also, light elements like Lithium (if present in the ionic liquid) might not be detected accurately.

The EDX spectrum provides valuable preliminary information about the NiFe_2_O_4_@MCM-41@IL/Pt nanocatalyst composition. It confirms the presence of all expected components and offers a semi-quantitative insight into their relative abundance.

**FIGURE 9:**
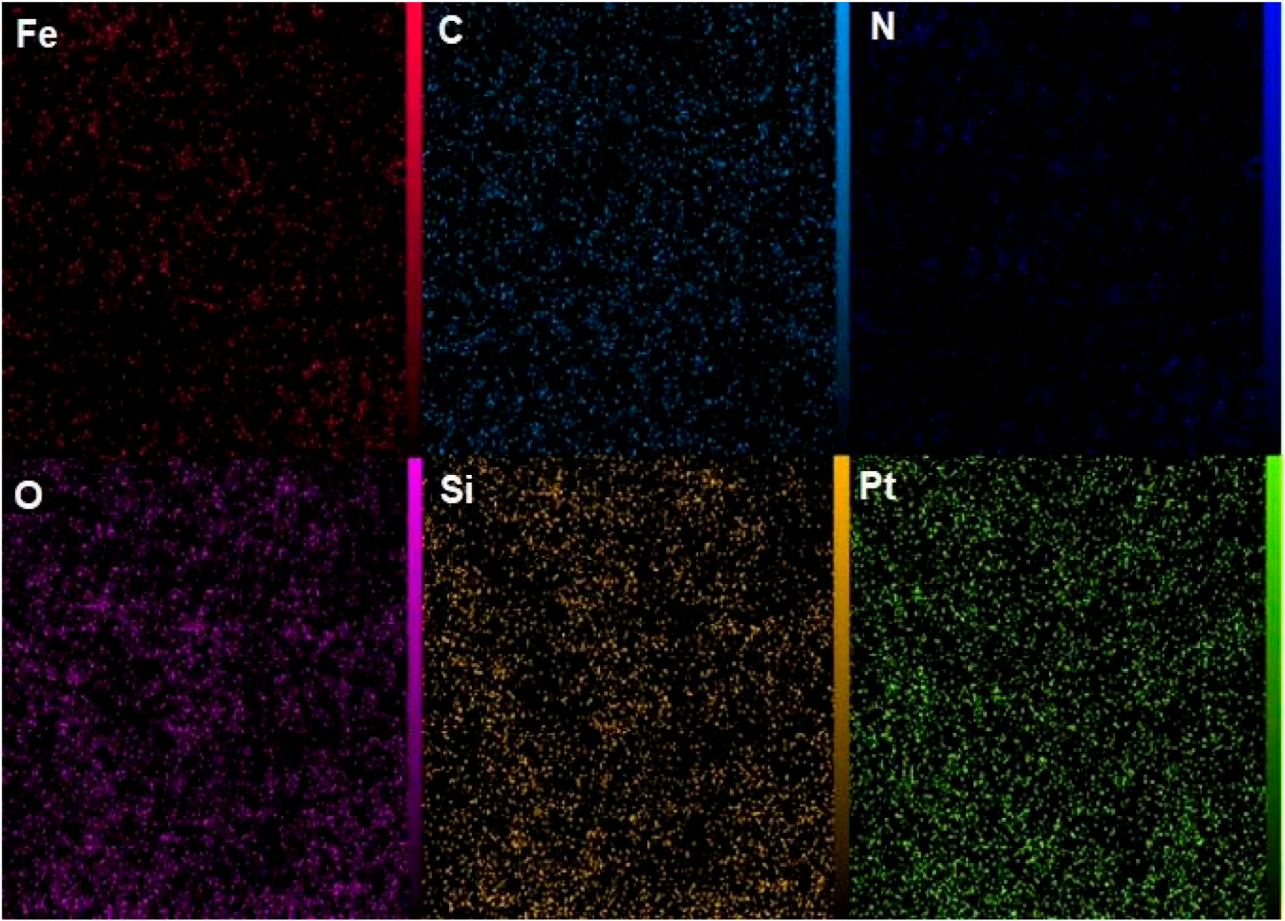
Elemental mapping analysis of NiFe_2_O_4_@MCM‐41@IL/Pt nanocatalyst.

Figure 9 presents the elemental mapping analysis of the NiFe_2_O_4_@MCM-41@IL/Pt nanocatalyst. This technique allows for the visualization and spatial distribution of individual elements within the complex nanostructure, providing crucial insights into the material’s composition and morphology.

The mapping reveals the presence and distribution of the following elements:

Fe (Iron): The iron distribution (red) indicates its presence primarily within the NiFe_2_O_4_ component. The relatively concentrated areas suggest the successful incorporation of iron into the spinel structure.

C (Carbon): The carbon mapping (teal) highlights the presence of the organic components, including the ionic liquid (IL) and the MCM-41 matrix. A uniform distribution suggests a homogeneous dispersion of these components within the catalyst structure.

N (Nitrogen): Nitrogen (blue) serves as an indicator for the presence of the ionic liquid (IL) component, confirming its successful incorporation into the catalyst system. The co-localization of nitrogen with carbon supports this assignment.

O (Oxygen): Oxygen (purple) is associated with the metal oxides (NiFe_2_O_4_ and potentially the silica of MCM-41). Its widespread distribution is consistent with the oxygen content in these components.

Si (Silicon): Silicon (yellow) is a distinctive marker for the MCM-41 mesoporous silica support. The distribution pattern reflects the structure of the silica matrix and confirms its successful integration into the catalyst.

Pt (Platinum): Platinum (green) mapping reveals the distribution of the platinum nanoparticles, which are a critical catalytic component. The distribution pattern suggests the dispersion of platinum throughout the catalyst structure, potentially supported on the MCM-41 or within the ionic liquid environment.

The elemental mapping provides valuable information regarding the successful synthesis of the NiFe_2_O_4_@MCM-41@IL/Pt nanocatalyst. The distinct signals for Fe, Si, and O confirm the presence of the NiFe_2_O_4_ and MCM-41 components. The detection of C and N and their co-localization confirm the successful incorporation of the ionic liquid. Notably, the Pt mapping demonstrates the presence and distribution of the platinum nanoparticles, which are essential for the material’s catalytic activity.

The elemental mapping presented in Figure 9 provides valuable insight into the composition and structure of the NiFe_2_O_4_@MCM-41@IL/Pt nanocatalyst. The successful mapping of each constituent element confirms the composite material’s successful synthesis and provides a basis for understanding its catalytic properties.

After identifying the structure of the NiFe_2_O_4_@MCM‐41@IL/Pt nanocomposite, its catalytic performance in the preparation of imidazo[1,2-a]pyrimidines was evaluated. Table 1 shows a summary of the optimization conditions for the synthesis of imidazo[1,2-a]pyrimidines (product 4k). The reaction involves 2-aminobenzimidazole (1) (1 mmol), benzaldehyde (2) (1 mmol), phenylacetylene (3) (1 mmol), NiFe_2_O_4_@MCM‐41@IL/Pt NPs (0–15 mg) water as a solvent (2 mL) was stirred at mentioned temperatures was evaluated under microwave irradiation. The table includes 21 entries, each detailing different catalysts, solvents, conditions (temperature and time), and yields.

**TABLE 1:** Optimization condition for synthesis of imidazo[1,2-a]pyrimidines (product 4k).

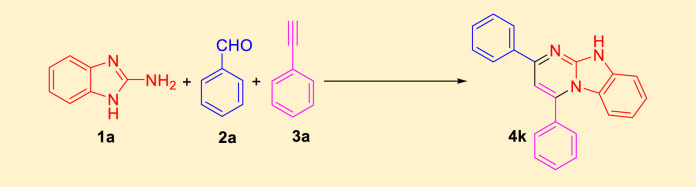						
Entry	Catalyst (mol%)	MW	Solvent	Temp. (^o^C)	Time (min)	Yield (%) ^a^
1	No catalyst	-	H_2_O	40°C	200	No
2	No catalyst	-	H_2_O	60°C	200	No
3	No catalyst	-	H_2_O	80°C	200	No
4	No catalyst	-	H_2_O	100°C	200	No
5	NiFe_2_O_4_@MCM‐41@IL/Pt (0.05)	100w	H_2_O	40°C	20	53
6	NiFe_2_O_4_@MCM‐41@IL/Pt (0.05)	100w	H_2_O	60°C	20	65
7	NiFe_2_O_4_@MCM‐41@IL/Pt (0.05)	100w	H_2_O	80°C	20	70
8	NiFe_2_O_4_@MCM‐41@IL/Pt (0.10)	100w	H_2_O	40°C	20	68
9	NiFe_2_O_4_@MCM‐41@IL/Pt (0.10)	100w	H_2_O	60°C	20	94
10	NiFe_2_O_4_@MCM‐41@IL/Pt (0.10)	100w	H_2_O	80°C	20	91
11	NiFe_2_O_4_@MCM‐41@IL/Pt (0.15)	100w	H_2_O	40°C	20	72
12	NiFe_2_O_4_@MCM‐41@IL/Pt (0.15)	100w	H_2_O	60°C	20	92
13	NiFe_2_O_4_@MCM‐41@IL/Pt (0.15)	100w	H_2_O	80°C	20	90
14	NiFe_2_O_4_@MCM‐41@IL/Pt (0.10)	80w	H_2_O	60°C	20	86
15	NiFe_2_O_4_@MCM‐41@IL/Pt (0.10)	120w	H_2_O	60°C	20	90
16	NiFe_2_O_4_@MCM‐41@IL/Pt (0.10)	150w	H_2_O	60°C	20	87
17	NiFe_2_O_4_@MCM‐41@IL/Pt (0.10)	100w	CH_3_CN	60°C	20	64
18	NiFe_2_O_4_@MCM‐41@IL/Pt (0.10)	100w	DMF	60°C	20	52
19	NiFe_2_O_4_@MCM‐41@IL/Pt (0.10)	100w	CH_2_Cl_2_	60°C	20	57
20	NiFe_2_O_4_@MCM‐41@IL/Pt (0.10)	100w	EtOH	60°C	20	90
21	NiFe_2_O_4_@MCM‐41@IL/Pt (0.10)	100w	EtOH:H_2_O (1:1)	60°C	20	86
22	No catalyst	100w	H_2_O	60°C	20	No
23	NiFe_2_O_4_@MCM‐41@IL/Pt (0.10)	-	H_2_O	60°C	20	No
Reaction conditions: 2-aminobenzimidazole (1 mmol), aldehyde (1 mmol), and terminal alkyne (1 mmol)						

^a^ Yields referred to isolated products.

A variety of organic solvents were explored, including 1,4-dioxane, methanol, ethanol, isopropanol, n-butanol, DMF, acetonitrile, chloroform, dichloromethane, DMSO, ethyl acetate, THF, and toluene. The best results were consistently obtained with water, yielding 94% product at 60°C. Without a catalyst, water proved to be an ineffective solvent for the reaction, yielding no product. However, when the NiFe_2_O_4_@MCM‐41@IL/Pt catalyst was introduced, water proved a suitable solvent, with yields increasing with temperatures up to 60°C. Higher temperatures generally led to higher yields, with the optimal temperature being 60°C for most solvents. The NiFe_2_O_4_@MCM‐41@IL/Pt catalyst was essential for the reaction to proceed. Without it, no product was formed. Based on the data, the following conditions are identified as the best for synthesizing compound (4k) is water in the presence of NiFe_2_O_4_@MCM‐41@IL/Pt NPs (0.10 mol%) at 60°C under MW.

In a controlled and precise experiment, we investigated the production of a broad and diverse range of imidazo[1,2-*a*]pyridine derivatives. This process used a one-step, three-component reaction method that included the use of aromatic and heteroaromatic aldehydes, along with 2-aminobenzimidazole derivatives, as well as terminal alkyne. This method allowed us to more accurately follow the production process of the desired derivatives and analyze the results carefully. The data in Table 2 demonstrates the successful creation of benzimidazole products with remarkably high yields.

**TABLE 2:** Scope of synthesis of imidazo[1,2-a]pyrimidines using NiFe_2_O_4_@MCM‐41@IL/Pt catalyst.

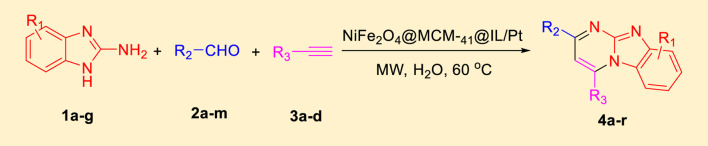		
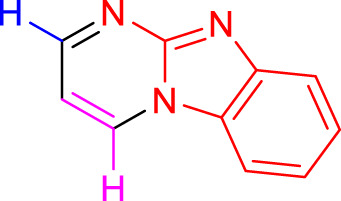	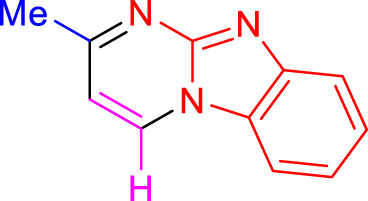	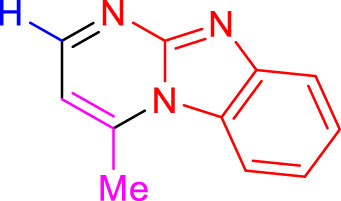
4a, 18 min, 95% (Shinde and Jeong, 2015)	4b, 17 min, 94%, (Kumar et al., 2014)	4c, 17 min, 92%, (Kumar et al., 2014)
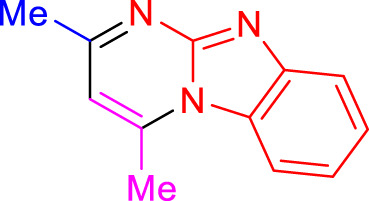	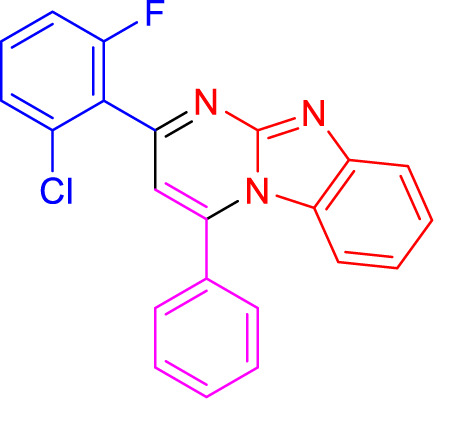	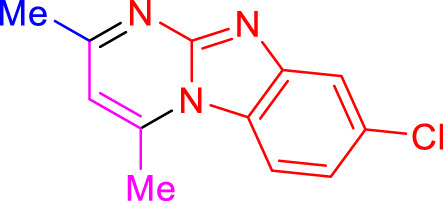
4days, 18 min, 93% (Rawat and Rawat, 2018)	4e, 18 min, 91% (Rawat and Rawat, 2018)	4f, 16 min, 90% (Shinde and Jeong, 2015)
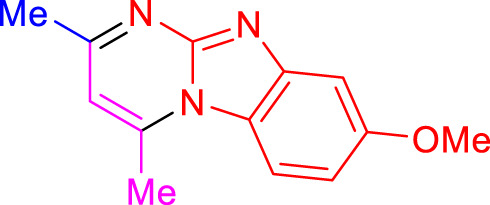 4g, 15 min, 91% (Jayashree and Shivashankar, 2019)	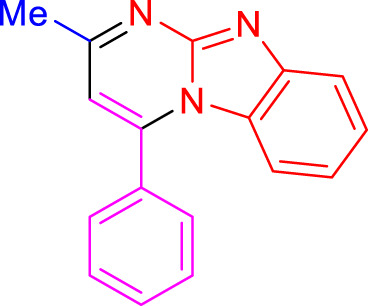 4h, 18 min, 90% (Jayashree and Shivashankar, 2019)	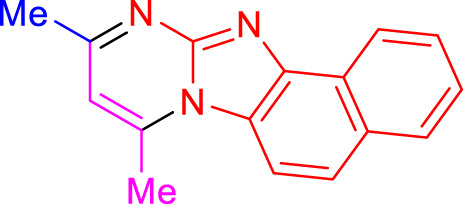 4i, 16 min, 91% (Shinde and Jeong, 2015)
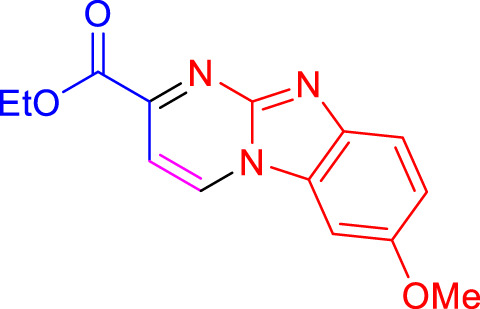	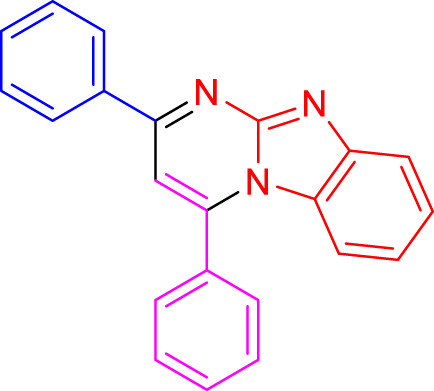	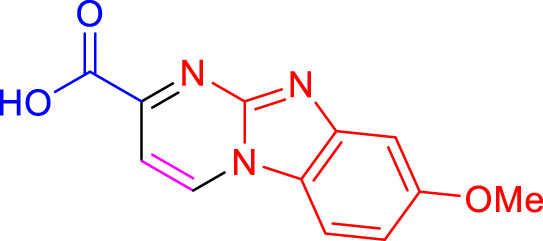
4j, 19 min, 90% (Rawat and Rawat, 2018)	4k, 20 min, 94% (Rawat and Rawat, 2018)	4L, 21 min, 89% (Kumar et al., 2014)
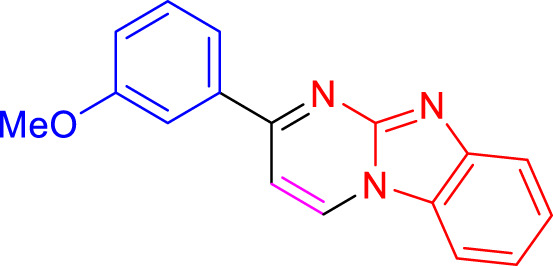	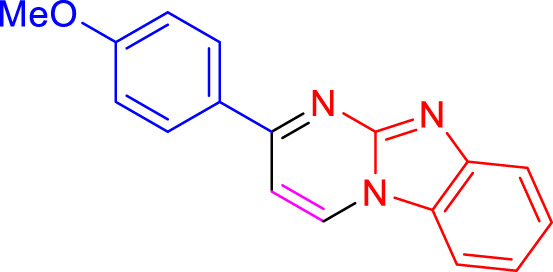	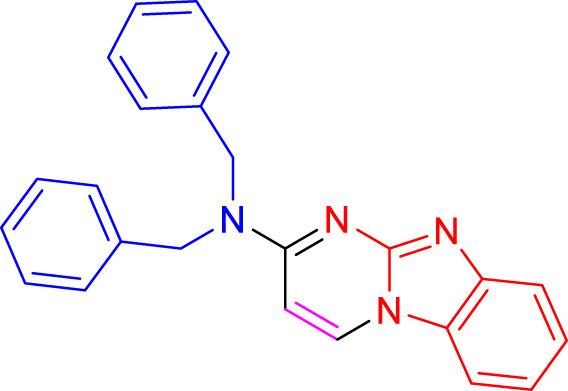
4m, 17 min, 94% (Kumar et al., 2014)	4n, 18 min, 96% (Shinde and Jeong, 2015)	4o, 22 min, 92% (Jayashree and Shivashankar, 2019)
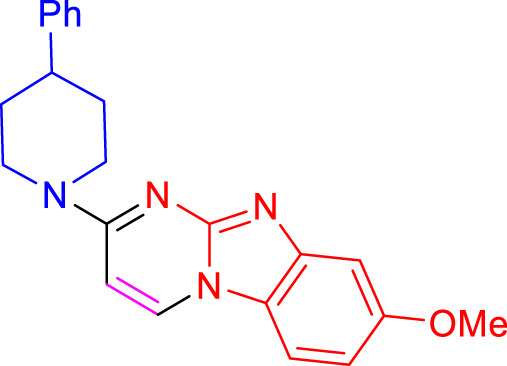	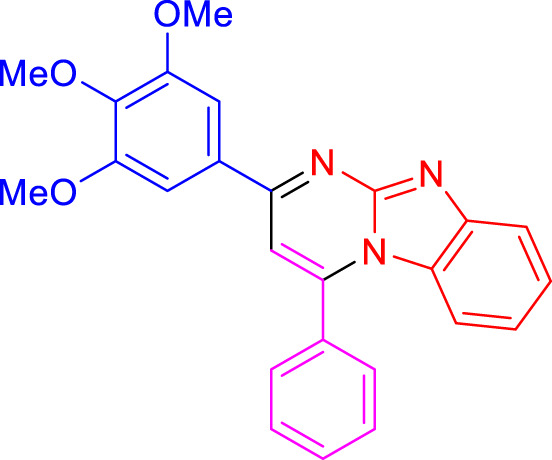	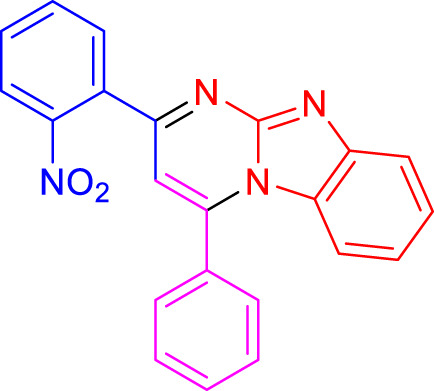
4p, 23 min, 90% (Rawat and Rawat, 2018)	4q, 15 min, 95% (Shinde and Jeong, 2015)	4r, 22 min, 90% (Jayashree and Shivashankar, 2019)

^a^ Yields referred to isolated products.

The electronic effects observed in the synthesis of imidazo[1,2-a]pyrimidines using the NiFe_2_O_4_@MCM-41@IL/Pt(II) catalyst are of significant importance, as they can greatly influence the reaction’s reactivity and selectivity. The table presented showcases the impact of various substituents on the aromatic rings of the reactants, providing a clear understanding of how electronic properties affect the overall yield and reaction time.

Firstly, electron-donating groups (EDGs), such as alkyl or methoxy substituents, enhance nucleophilicity in amines or electrophilicity in carbonyl compounds. This increased nucleophilicity can lead to faster reaction rates, as seen in entries where these groups are present. For instance, in a reaction involving [specific reactants], compounds with methoxy groups show shorter reaction times and higher yields than those with electron-withdrawing groups (EWGs) like [specific reactants]. EDGs stabilize positive charges that may develop during intermediate formation, facilitating smoother transitions through reactive states.

Conversely, electron-withdrawing groups like nitro or halogens can decrease nucleophilicity and increase electrophilicity. Due to their inductive effects, these groups can create a less favorable environment for nucleophilic attacks, which can result in longer reaction times and lower yields. For example, when a nitro group is present on one of the aromatic rings, the reaction proceeds more slowly because it destabilizes intermediates by increasing electron deficiency.

Moreover, steric effects also play a role alongside electronic factors. Bulky substituents can hinder access to reactive sites on the substrate or catalyst, further influencing reaction kinetics. The combination of steric hindrance with electronic effects often dictates the overall efficiency of the synthesis.

Understanding these electronic effects is an academic exercise and a crucial step toward optimizing conditions for synthesizing imidazo[1,2-a]pyrimidines. By strategically selecting substituents based on their electronic properties, chemists can tailor reactions to achieve desired products more efficiently while maximizing yields. The table’s data highlights this interplay between electronic effects and catalytic performance and provides practical insights for future research and applications.

As depicted in the provided Scheme 2, the mechanism for synthesizing imidazo[1,2-a]pyrimidine derivatives involves several critical steps. These steps are significantly facilitated by the Fe_2_NiO_4_@MCM-41@IL/Pt (II) nanocomposite catalyst, a pivotal element that enhances the reaction efficiency and selectivity. Understanding the role of this catalyst is crucial for a comprehensive grasp of the synthesis process.

The reaction begins with the nucleophilic attack of an amine (compound 1) on a carbonyl or imine intermediate, leading to the formation of an intermediate denoted as A. In this step, water is eliminated, and the catalyst facilitates the activation of the substrates by providing a suitable environment that lowers the activation energy required for bond formation. Platinum within the catalyst structure enhances its Lewis acidity, promoting electrophilic character in the substrate and thus facilitating this initial nucleophilic attack.

Subsequently, intermediate A undergoes further transformation, where another amine reacts with it in a subsequent step. This reaction leads to another intermediate (denoted as B), where additional water is released. The catalyst’s role here is crucial; it not only accelerates the reaction but also ensures high regioselectivity toward desired products by stabilizing transition states and intermediates through coordination interactions. This reassures us of the catalyst’s effectiveness in the synthesis process.

In the final stage of the mechanism, intermediate B undergoes cyclization to yield imidazo[1,2-a]pyrimidine derivatives (compound 4). This step is critical as it involves forming new bonds that define the final product’s structure. The catalytic support provided by Fe_2_NiO_4_@MCM-41@IL/Pt(II) ensures that these reactions proceed smoothly by maintaining a favorable reactant microenvironment while facilitating effective substrate alignment. Effective substrate alignment refers to the arrangement of the reactants in a way that maximizes the chances of successful reaction, and the catalyst ensures this alignment, thereby promoting the formation of the desired product.

The role of the catalyst throughout this mechanism is paramount. It provides active sites for substrate binding and stabilizes intermediates through coordination interactions, significantly enhancing both reaction rates and yields. This underscores the catalyst’s crucial contribution to the reaction, instilling confidence in its effectiveness.

This mechanism illustrates how Fe_2_NiO_4_@MCM-41@IL/Pt (II) serves as a facilitator of chemical transformations and as a means to improve overall process efficiency in synthesizing valuable imidazo [1,2-*a*]pyrimidine derivatives.

**FIGURE 10:**
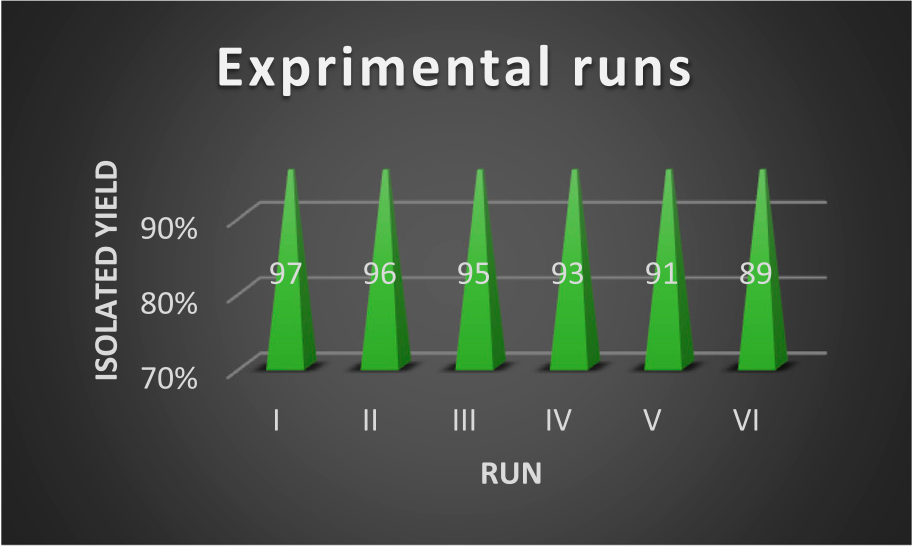
Reusability of NiFe_2_O_4_@MCM‐41@IL/Pt catalyst on the model reaction (product 4k).

Simple catalyst separation and reusability are important factors in modern catalyst science. Consequently, the reusability of the v@MCM‐41@IL/Pt catalyst was evaluated in the synthesis of product 4k. Following the reaction, the NiFe_2_O_4_@MCM‐41@IL/Pt catalyst was recovered through magnetic decantation, washed with ethyl acetate and ethanol, dried, and reused. The results of the reusability tests reflected that the NiFe_2_O_4_@MCM‐41@IL/Pt catalyst could be employed up to 6 times without substantially reducing its efficiency (Figure 10). FT-IR, VSM, and BET analyses were used to investigate the structure of the recovered catalyst (Figure 10). VSM analysis confirmed that the regenerated catalyst still has high magnetic properties. Also, FT-IR and BET analyses confirmed that the recovered catalyst has remarkable stability because the structure and shape of the particles in the analyses before and after recovery were almost the same.

**FIGURE 11:**
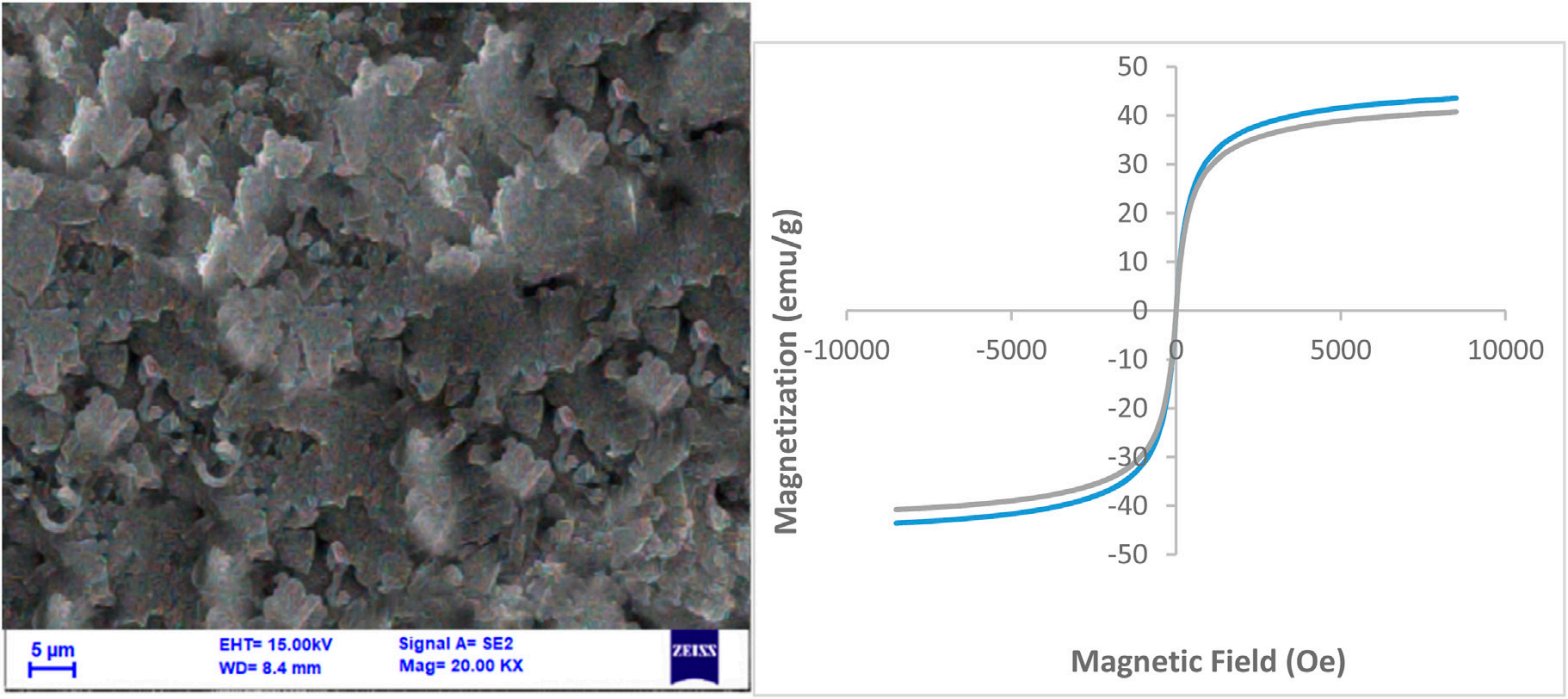
VSM and BET analysis of the recovered NiFe_2_O_4_@MCM‐41@IL/Pt nanocatalyst after 6 times.

The left SEM image provides a visual representation of the morphology and microstructure of the recovered NiFe_2_O_4_@MCM-41@IL/Pt nanocatalyst in Figure 11. The image reveals a porous and agglomerated structure with particles ranging from approximately 5–10 μm. The surface appears rough and uneven, indicating the presence of numerous pores and cavities.

The VSM (Vibrating Sample Magnetometer) curve on the right shows the magnetic hysteresis loop of the recovered catalyst. The curve exhibits a typical S-shaped behavior, characteristic of ferromagnetic materials. The following observations can be made from the VSM data:

Saturation Magnetization (Ms): The saturation magnetization value is approximately 45 emu/g. This indicates a relatively high magnetic moment of the catalyst.

Coercivity (Hc): The coercivity is around 10 Oe. This value suggests that the catalyst possesses a moderate degree of magnetic hardness.

Remanence (Mr): The remanence is approximately 20 emu/g. This value signifies residual magnetization in the catalyst even after the applied magnetic field is removed.

The VSM analysis suggests that the recovered catalyst retains its magnetic properties after six use cycles. The high saturation magnetization and moderate coercivity indicate that an external magnetic field can easily separate the catalyst from the reaction mixture. However, the presence of agglomeration in the SEM image might hinder the separation process to some extent.

Additional VSM measurements at different temperatures would be beneficial to gain a deeper understanding of the catalyst’s magnetic properties. This would provide insights into the temperature dependence of the magnetic parameters and the nature of magnetic interactions within the catalyst.

The BET (Brunauer-Emmett-Teller) analysis, not shown in the image, determines the catalyst’s specific surface area and pore size distribution. This information is crucial for understanding the catalyst’s activity and stability.

The image indicates that the catalyst was recovered after six cycles. It would be interesting to compare the VSM and BET data of the fresh and recovered catalysts to assess the impact of multiple cycles on their properties.

The recovered NiFe_2_O_4_@MCM-41@IL/Pt nanocatalyst’s SEM and VSM analysis suggest that it retains its magnetic properties after six cycles of use. However, further analysis, including BET analysis and VSM measurements at different temperatures, is required to understand the catalyst’s properties and behavior fully.

The FT-IR spectrum of the recovered catalyst shows significant differences compared to the fresh catalyst. The observed differences between the fresh and recovered catalyst spectra suggest that the catalyst undergoes significant changes during the reaction process. The decrease in intensity of the O-H and C-H stretching peaks indicates a potential loss of active sites on the catalyst surface. Additionally, the broadening and reduction in intensity of the carbonyl and aromatic peaks suggest changes in the catalyst’s electronic structure and surface properties.

**FIGURE 12:**
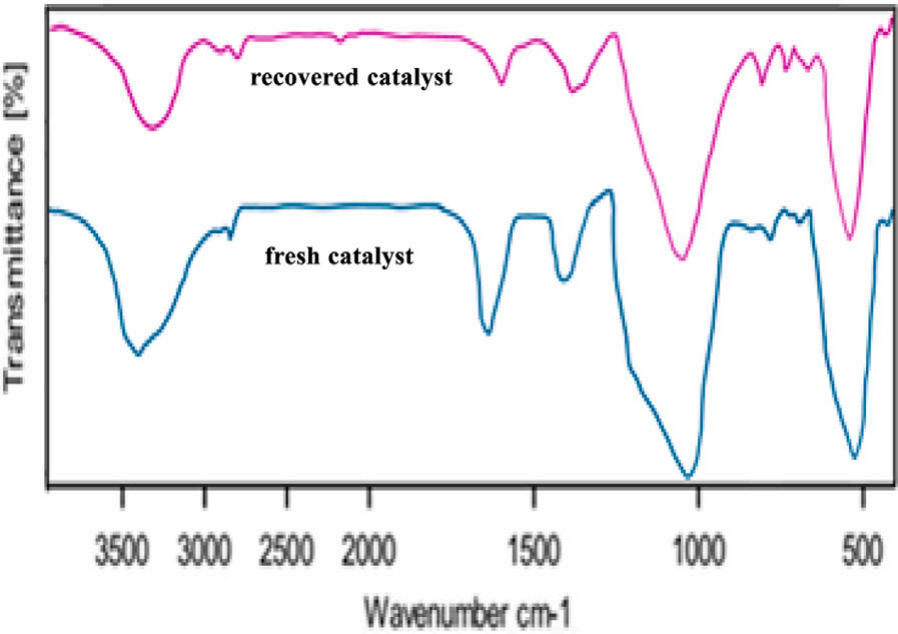
FT-IR spectrums of fresh catalyst and recovered catalyst (after 6 times).

The FT-IR spectra of the fresh and recovered catalyst (Figure 12) revealed significant differences, particularly in the regions corresponding to O-H and C-H stretching vibrations. The decrease in intensity of these peaks in the recovered catalyst suggests a potential loss of active sites due to coking and/or metal leaching. Additionally, the broadening and reduction in intensity of the carbonyl and aromatic peaks indicate changes in the catalyst’s electronic structure and surface properties. Further investigations are necessary to elucidate the exact mechanisms of these changes and their impact on the catalyst’s performance.

### 2.1 Hot filtration test (leaching)

**FIGURE 13:**
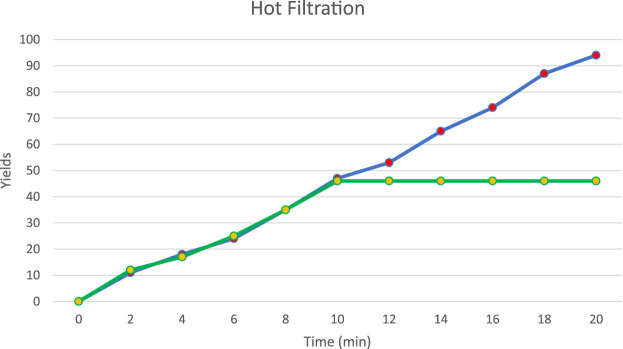
Hot filtration test (leaching) catalyst.

To assess nickel leakage from the catalyst during the reaction, a leaching test was conducted using a hot filtration method for the click reaction involving 2-aminobenzimidazole (1), aldehyde (2), and terminal alkyne (3). After 10 min, the catalytically active particles were removed from the reaction mixture through hot filtration, and the filtrate was observed for any continued activity. Following the hot filtration, the reaction yield stabilized at approximately 46% and showed no further changes (Figure 13).

Table 3 compares the efficiency of different methods for synthesizing 2-(phenylthio)benzo [*d*]thiazole (product 4k). Efficiency is evaluated based on the time required for the reaction and the product yield. The table shows that the method developed in this study, using NiFe_2_O_4_@MCM‐41@IL/Pt as a catalyst, is the most efficient. It requires only 3 h to complete and yields 95%. Other methods reported in the literature typically require longer reaction times and lower yields.

**TABLE 3:** Comparison of the efficiency of this method with reported methods for the synthesis of imidazo[1,2-a]pyridine derivatives (product 4k) as the model reaction.

Entry	Catalyst	Condition	Time (min)	Yield (%)	Ref.
1	CuO NPs	Solvent-free, 100°C	60	84	56
2	CuI/Ag_2_CO_3_	CH_3_CN, reflux	300	72	57
3	MSA	Solvent-free, 85°C	120	68	58
4	sulfated copper oxide	CH_3_CN, reflux	180	85	59
5	NiFe_2_O_4_@MCM‐41@IL/Pt	MW, 60°C	20	94	This Method

## 3 Experimental

All chemicals were purchased from Sigma and Merck. The reagents and solvents used in this work were obtained from Sigma-Aldrich, Fluka, or Merck and used without further purification. The samples’ infrared spectra (IR) were recorded in KBr disks using a NICOLET impact 410 spectrometer. ^1^HNMR and ^13^CNMR spectra were recorded with a Bruker DRX-400 spectrometer at 400 and 100MHz, respectively.

### 3.1 Synthesis of nanocatalyst

#### 3.1.1 Synthesis of NiFe_2_O_4_ nanoparticles

NiFe_2_O_4_ was fabricated via a co-precipitation chemical process. FeCl_2_·4H_2_O and Ni(Cl)_2_·9H_2_O were initially dissolved in 100 mL of water, maintained under a nitrogen atmosphere at 80°C with a molar ratio of 2:1. Following this, 10 mL of 0.2 M NaOH solution was incrementally added over 10 min to the agitated mixture, achieving a final pH of 12. After 30 min of continuous stirring, the NiFe_2_O_4_ MNPs were magnetically separated, washed multiple times with deionized water, and dried at 75°C overnight.

#### 3.1.2 Synthesis of NiFe_2_O_4_@SiO_2_

The interlayers of SiO_2_ were prepared through a modified Stober method (Dai et al., 2017). In order to synthesize NiFe_2_O_4_@MCM‐41, first, NiFe_2_O_4_@SiO_2_ NPs were prepared according to a well‐known procedure (Cui et al., 2013). Then, 0.6 g of NiFe_2_O_4_@SiO_2_ nanoparticles were added to distilled water (50 mL) and EtOH (110 mL) and dispersed under ultrasonic irradiation at 30°C for 30 min. In the next step, 3 mL of aqueous ammonia solution (25%) and 1 g of cetyltrimethylammonium bromide (CTAB) were added to the resulting mixture, and it was mechanically stirred at room temperature for 10 min. Then 0.7 mL of tetraethoxysilane (TMOS) was added dropwise, and the resulting mixture was stirred for 2 h at room temperature. After that, this was statically heated at 100°C for 48 h. Finally, the resulting material was separated using an external magnet, washed with deionized water, and dried at 70°C for 10 h. The CTAB template was removed from the synthesized material by calcination at 400°C for 6 h [62].

#### 3.1.3 Synthesis of NiFe_2_O_4_@MCM‐41@IL

The NiFe_2_O_4_@MCM‐41@IL nanomaterial was prepared: 1.0 g of NiFe_2_O_4_@MCM‐41 nanoparticles were suspended in 50 mL of toluene and sonicated for 20 min at room temperature. Then, 0.53 mmol of 1,3-bis(3-(trimethoxysilyl)propyl)-1*H*-imidazol-3-ium chloride was added, and the mixture was refluxed under an argon atmosphere for 24 h in an oil bath. The resulting solid was separated using an external magnet, washed thoroughly with ethanol, and labeled NiFe_2_O_4_@MCM‐41@IL.

#### 3.1.4 Synthesis of NiFe_2_O_4_@MCM‐41@IL/Pt

For the preparation of NiFe_2_O_4_@MCM‐41@IL/Pt nanocatalyst, typically 1 g of NiFe_2_O_4_@MCM‐41@IL was added in dimethyl sulfoxide (DMSO, 20 mL) and sonicated for 10 min at room temperature. After the complete dispersion of this material, 1.5 mmol of Pt(Cl)_2_ salt was added into the reaction vessel and the obtained mixture was first stirred at room temperature for 24 h and then heated for 2 h at 80°C. After that, the resulting material was magnetically separated, washed thoroughly with DMSO, dried at 65°C for 10 h, and labeled as NiFe_2_O_4_@MCM‐41@IL/Pt.

### 3.2 General procedure for the preparation of imidazo [1,2-*a*]pyridine derivatives

2-Aminobenzimidazole (1 mmol), arylaldehyde (1 mmol), and phenylacetylene (2 mmol) were combined with NiFe_2_O_4_@MCM‐41@IL/Pt nanocatalyst (10 mg) and 5 mL of distilled water in a 10 mL initiator reaction vial. The vial was hermetically sealed and pressurized for 20 s before microwave irradiation at 60°C with a power output of 100 W. The vial was microwaved until thin-layer chromatography (TLC) using n-hexane–ethyl acetate (2:1) showed complete consumption of the starting substances. After cooling the reaction mixture to room temperature, the magnetic organocatalyst was separated using a magnetic field. The remaining residue was subsequently purified through recrystallization from ethanol to obtain the desired product with high yields.

## 4 Conclusion

This study demonstrates the successful development of a novel, eco-friendly NiFe_2_O_4_@MCM-41@IL/Pt magnetic nanocatalyst for the efficient synthesis of benzo[4,5]imidazo[1,2-a]pyrimidines via microwave-assisted A^3^ coupling reactions. By integrating the magnetic properties of NiFe_2_O_4_, the high surface area of MCM-41, the stabilizing effects of ionic liquids, and the catalytic activity of platinum, this hybrid system achieves exceptional performance in water as a green solvent, energy-efficient conditions. The microwave irradiation protocol significantly accelerated reaction kinetics, enabling the formation of target heterocycles in 15–25 min with yields of 89%–96%, while the catalyst’s magnetic core allowed facile recovery and reuse over five cycles without appreciable loss in activity. The methodology exhibits an impressive broad substrate compatibility, accommodating diverse aromatic and heteroaromatic aldehydes, 2-aminobenzimidazole derivatives, and terminal alkynes to generate pharmaceutically relevant scaffolds. This versatility of the catalyst instills optimism about its potential applications. This approach aligns with green chemistry principles by eliminating toxic solvents, minimizing waste, and reducing energy consumption. Mechanistic insights into the synergistic roles of the catalyst components underscore the importance of Pt-mediated alkyne activation and IL-enhanced stabilization of intermediates. This work represents a significant advancement in sustainable heterocyclic chemistry, offering a scalable, environmentally benign alternative to traditional multi-step syntheses. The developed protocol streamlines access to bioactive imidazopyridine and highlights the potential of integrating microwave technology with recyclable nanocatalysts for diverse organic transformations. Future studies will explore applications in industrial-scale synthesis and adaptations to other multicomponent reactions.

## Data Availability

The original contributions presented in the study are included in the article/Supplementary Material, further inquiries can be directed to the corresponding author.
