# Supplementary material for: Magnetic nanocatalyst for microwave-assisted synthesis of Benzo[4,5]imidazo[1,2-a]pyrimidines via A3 coupling
Source: Front Chem. 2025 Jul 14;13:1631183. doi: 10.3389/fchem.2025.1631183 (PMC12302415; doi:10.3389/fchem.2025.1631183)

**Presentation a novel and green magnetic nanocatalyst for preparation of**

**benzo[4,5]imidazo[1,2-*a*]pyrimidines via microwave-assisted A3 coupling reactions**

**Yuqiang Pan**

Guangxi Guida Agricultural Technology Co., Ltd Nanning, Guangxi 530000 China

Corresponding Author: pany42976@gmail.com

**benzo[4,5]imidazo[1,2-a]pyrimidine (4a)**

M.P: 181-183 ^o^C; ^1^H NMR (400 MHz, CDCl_3_) δ 8.52 (d, J = 8.2 Hz, 1H), 8.48 (d, J = 8.4 Hz, 1H), 8.33 (dd, J = 9.0, 2.5 Hz, 1H), 7.65 (d, J = 7.6 Hz, 1H), 7.45 – 7.40 (m, 2H), 7.34-7.30 (m, 1H) ppm. ^13^C NMR (101 MHz, CDCl_3_) δ 157.8, 149.0, 143.4, 139.1, 127.6, 123.8, 121.0, 119.6, 116.5, 106.4 ppm.

**2-methylpyrimido<1,2-a>benzimidazole(4b)**

M.P: 230-232 ^o^C; ^1^H NMR (400 MHz, CDCl_3_) δ 8.61 (d, J = 7.6 Hz, 1H), 8.56 (d, J = 8.0 Hz, 1H), 7.53 (d, J = 8.4 Hz, 2H), 7.37– 7.17 (m, 3H), 2.45 (s, 3H) ppm. ^13^C NMR (101 MHz, CDCl_3_) δ 165.4, 151.3, 140.9, 137.6, 136.9, 128.7, 127.8, 125.4, 122.0, 121.9, 117.1, 23.0 ppm.

**4-methyl-benzo[4,5]imidazo[1,2-a]pyrimidine (4c)**

M.P: 224-226 ^o^C; 1H NMR (400 MHz, CDCl3) δ 8.83 (d, J = 7.7 Hz, 1H), 7.41 (d, J = 8.1 Hz, 1H), 7.85 (d, J = 7.9 Hz, 1H), 7.53 – 7.37 (m, 3H), 2.27 (s, 3H) ppm. 13C NMR (101 MHz, CDCl3) δ 167.8, 160.1, 153.4, 143.6, 137.2, 124.3,121.0, 120.9, 119.7, 117.3, 20.7 ppm.

**1,3-dimethylpyrimido[1,2-a]benzimidazole( 4d)**

M.P: 234-236 ^o^C; 1H NMR (400 MHz, CDCl3) δ 8.33 (d, J = 7.4 Hz, 1H), 7.65 (d, J = 7.7 Hz, 1H), 7.49 (s, 1H), 7.15 – 7.13 (m, 1H), 7.06-7.00 (m, 1H), 3.20 (s, 3H), 2.71 (s, 3H) ppm. 13C NMR (101 MHz, CDCl3) δ 167.1, 165.4, 150.8, 138.2, 150.8, 138.2, 135.4, 125.4, 123.6, 121.9, 121.7, 115.8, 108.4, 23.5, 20.8 ppm.

**2-(2-chloro-6-fluorophenyl)-4-phenylbenzo[4,5]imidazo[1,2-a]pyrimidine (4e)**

M.P: 247-249 ^o^C; 1H NMR (400 MHz, CDCl3) δ 8.67 (d, J = 8.4 Hz, 1H), 8.16 (d, J = 8.2 Hz, 2H), 7.83 (s, 1H), 7.76 (d, J = 7.9 Hz, 1H), 7.55-7.51 (m, 3H), 7.32 – 7.23 (m, 3H), 7.01-6.95 (m, 2H) ppm. 13C NMR (101 MHz, CDCl3) δ 165.3, 158.2, 150.7, 133.2, 132.0, 130.7, 129.8, 128.6, 127.0, 125.2, 120.7, 119.5, 118.4, 115.3, 113.6, 110.9, 105.5 ppm.

**1,3-dimethyl-7-chloropyrimido<1,2-a>benzimidazole (4f)**

M.P: 278-280 ^o^C; 1H NMR (400 MHz, CDCl3) δ 8.38 (s, 1H), 7.60-7.53 (m, 1H), 7.40 (s, 1H), 7.33 (d, J = 8.4 Hz, 1H), 2.78 (s, 3H), 2.32 (s, 3H) ppm. 13C NMR (101 MHz, CDCl3) δ 160.4, 150.7, 141.1, 138.9, 135.3, 126.5, 124.3, 122.9, 121.0, 117.6, 116.3, 24.0, 20.9 ppm.

**7-methoxy-1,3-dimethylpyrimido[1,2-a]benzimidazole (4g)**

M.P: 188-190 ^o^C; 1H NMR (400 MHz, CDCl3) δ 8.38 (dd, J = 8.3, 4.2 Hz, 1H), 8.20 (s, 1H), 7.63 (s, 1H), 7.56 (d, J = 8.2 Hz, 1H), 3.81 (s, 3H), 2.76 (s, 3H), 2.40 (s, 3H) ppm. 13C NMR (101 MHz, CDCl3) δ 164.0, 163.9, 158.1, 149.8, 143.3, 128.7, 119.6, 118.5, 116.3, 116.0, 60.1, 23.7, 20.1 ppm.

**2-methyl-4-phenylbenzo[4,5]imidazo[1,2-a]pyrimidine (4h)**

M.P: 197-199 ^o^C; 1H NMR (400 MHz, CDCl3) δ 8.55 (d, J = 8.4 Hz, 1H), 7.83 (s, 1H), 7.76 (d, J = 8.0 Hz, 2H), 7.55-7.50 (m, 3H), 7.33 (d, J = 7.7 Hz, 1H), 7.22-7.16 (m, 2H), 2.43 (s, 3H) ppm. 13C NMR (101 MHz, CDCl3) δ 163.2, 150.4, 139.7, 136.7, 134.3, 130.9, 128.7, 127.5, 126.3, 121.3, 120.8, 115.1, 107.6, 21.0 ppm.

**1,3-dimethylpyrimido[1,2-a]naphtho[1,2-d]imidazole (4i)**

M.P: 234-236 ^o^C; 1H NMR (400 MHz, CDCl3) δ 8.45 (d, J = 8.0 Hz, 1H), 8.24 (d, J = 7.9 Hz, 1H), 8.03 (d, J = 8.0 Hz, 1H), 7.84 – 7.78 (m, 2H), 7.62 (d, J = 8.4 Hz, 1H), 7.24 (s, 1H), 2.56 (s, 3H), 2.28 (s, 3H) ppm. 13C NMR (101 MHz, CDCl3) δ 166.5, 165.4, 151.2, 149.0, 133.7, 131.9, 128.0, 127.2, 120.8, 116.3, 23.9, 20.7 ppm.

**ethyl 4-methoxy-1,8,10-triazatricyclo[7.4.0.02,7]trideca-2(7),3,5,8,10,12-hexaene-11-carboxylate (4j)**

1H NMR (400 MHz, CDCl3) δ 9.24 (d, J = 8.4 Hz, 1H), 8.42 (d, J = 7.7 Hz, 1H), 7.53 (dd, J = 7.6 Hz, 1H), 7.34 (s, 1H), 7.16 (d, J = 8.0 Hz, 1H), 4.19 (q, J = 7.6 Hz, 2H), 3.90 (s, 3H), 1.32 (t, J = 7.6 Hz, 3H) ppm. 13C NMR (101 MHz, CDCl3) δ 167.5, 150.1, 138.4, 132.1, 130.9, 128.5, 128.0, 121.7, 120.5, 118.4, 117.3, 100.4, 62.1, 57.3, 20.8 ppm.

**5-methoxy-1,8,10-triazatricyclo[7.4.0.02,7]trideca-2,4,6,8,10,12-hexaene-11-carboxylic acid (4k)**

M.P: 271-273 ^o^C; 1H NMR (400 MHz, CDCl3) δ 10.90 (s, 1H), 9.42 (d, J = 7.9 Hz, 1H), 8.63 (d, J = 7.7 Hz, 1H), 8.53 (dd, J = 8.6, 4.0 Hz, 1H), 8.12 (s, 1H), 7.97 (dd, J = 8.0,3.9 Hz, 1H), 3.76 (s, 3H) ppm. 13C NMR (101 MHz, CDCl3) δ 168.1, 157.9, 143.2, 131.7, 129.8, 129.7, 128.5, 126.2, 120.8, 118.3, 117.4, 115.9, 100.6, 60.5 ppm.

**2-(3-methoxyphenyl)benzo[4,5]imidazo[1,2-a]pyrimidine (4l)**

M.P: 202-204 ^o^C; 1H NMR (400 MHz, CDCl3) δ 8.63 (d, J = 7.9 Hz, 1H), 8.53 (d, J = 8.0Hz, 1H), 8.25 (d, J = 8.6 Hz, 1H), 8.19 (d, J = 7.6 Hz, 2H), 7.88 (s, 1H), 7.76 – 7.72 (m, 2H), 7.67-7.64 (m, 1H), 7.53 (d, J = 7.6 Hz, 1H), 3.80 (s, 3H)ppm. 13C NMR (101 MHz, CDCl3) δ 161.9, 154.8, 140.9, 137.8, 125.3, 124.6, 124.0, 122.0, 121.9, 117.6, 116.9, 113.2, 110.7, 58.7 ppm.

**2-(4-methoxyphenyl)benzo[4,5]imidazo[1,2-a]pyrimidine (4m)**

M.P: 196-198 ^o^C; 1H NMR (400 MHz, CDCl3) δ 8.65 (d, J = 8.6 Hz, 1H), 8.56 (d, J = 8.4 Hz, 1H), 7.87 (d, J = 7.6 Hz, 1H), 7.73 (d, J = 7.6 Hz, 2H), 7.61 (d, J = 7.6 Hz, 1H), 7.47 – 7.40 (m, 1H), 7.36-7.31 (m, 1H), 7.20 (d, J = 7.8 Hz, 1H), 3.73 (s, 3H) ppm. 13C NMR (101 MHz, CDCl3) δ 164.1, 151.6, 147.2, 132.7, 131.0, 129.6, 127.3, 125.4, 124.3, 118.7, 117.0, 116.4, 115.0, 114.2, 60.7 ppm.

**N,N-dibenzylbenzo[4,5]imidazo[1,2-a]pyrimidin-2-amine (4n)**

M.P: 191-193 ^o^C; 1H NMR (400 MHz, CDCl3) δ 8.24 (d, J = 8.0 Hz, 1H), 7.96 (d, J = 7.9 Hz, 1H), 7.81 (d, J = 7.6 Hz, 1H), 7.74 (t, J = 7.6 Hz, 4H), 7.46-7.41 (m, 2H), 7.34 – 7.30 (m, 1H), 7.27 (d, J = 7.8 Hz, 4H), 7.16-7.12 (m, 1H), 6.86 (d, J = 7.8 Hz, 1H), 4.55 (s, 4H) ppm. 13C NMR (101 MHz, CDCl3) δ 165.4, 150.9, 143.2, 139.0, 138.4, 124.3, 123.2, 121.0, 120.7, 119.0, 118.4, 117.6, 113.7, 113.0, 60.3 ppm.

**8-methoxy-2-(4-phenylpiperidin-1-yl)benzo[4,5]imidazo[1,2-a]pyrimidine (4o)**

M.P: 250-252 ^o^C; 1H NMR (400 MHz, CDCl3) δ 8.12(dd, J = 8.6, 3.5 Hz, 1H), 7.95 (d, J = 8.0 Hz, 1H), 7.73 (d, J = 7.7 Hz, 1H), 7.65 (d, J = 7.9 Hz, 1H), 7.56 (s, 1H), 7.23 – 7.16 (m, 5H), 3.81 (s, 3H), 2.65-2.63 (m, 2H), 2.17-2.15 (m, 2H), 1.97-1.94 (m, 2H), 1.30-1.26 (m, 2H) ppm. 13C NMR (101 MHz, CDCl3) δ 168.0, 153.2, 149.7, 145.3, 140.6, 128.4, 127.6, 125.2, 124.1, 122.8, 120.6, 119.7, 118.0, 112.3, 100.4, 59.8, 54.3, 41.0, 28.7 ppm.

**4-phenyl-2-(3,4,5-trimethoxyphenyl)benzo[4,5]imidazo[1,2-a]pyrimidine (4p)**

M.P: 231-233 ^o^C; 1H NMR (400 MHz, CDCl3) δ 8.65 (s, 1H), 8.93 (s, 2H), 7.75 (d, J = 8.6 Hz, 2H), 7.53 (t, J = 8.6 Hz, 2H), 7.25 (d, J = 7.6 Hz, 2H), 7.18-7.11 (m, 3H), 3.80 (s, 6H), 3.77 (s, 3H) ppm. 13C NMR (101 MHz, CDCl3) δ 166.0, 160.6, 158.1, 147.3, 137.6, 135.4, 132.0, 130.6, 129.8, 121.2, 119.6, 115.0, 106.4, 60.7, 57.3 ppm.

**2-(2-nitrophenyl)-4-phenylbenzo[4,5]imidazo[1,2-a]pyrimidine (4q)**

M.P: 219-221 ^o^C; ^1^H NMR (400 MHz, CDCl_3_) δ 8.18 (d, J = 7.8 Hz, 1H), 7.65 (s, 1H), 7.56-7.50 (m, 2H), 7.46 (d, J = 7.6 Hz, 2H), 7.36 (dd, J = 7.7 Hz, 2H), 7.07 (d, J = 8.2 Hz, 2H), 6.97-6.84 (m, 3H) ppm. ^13^C NMR (101 MHz, CDCl_3_) δ 168.5, 163.0, 152.9, 133.9, 132.7, 130.5, 129.4, 128.7, 127.2, 125.4, 124.1, 120.9, 119.6, 118.4, 117.3, 116.1, 114.2 ppm.


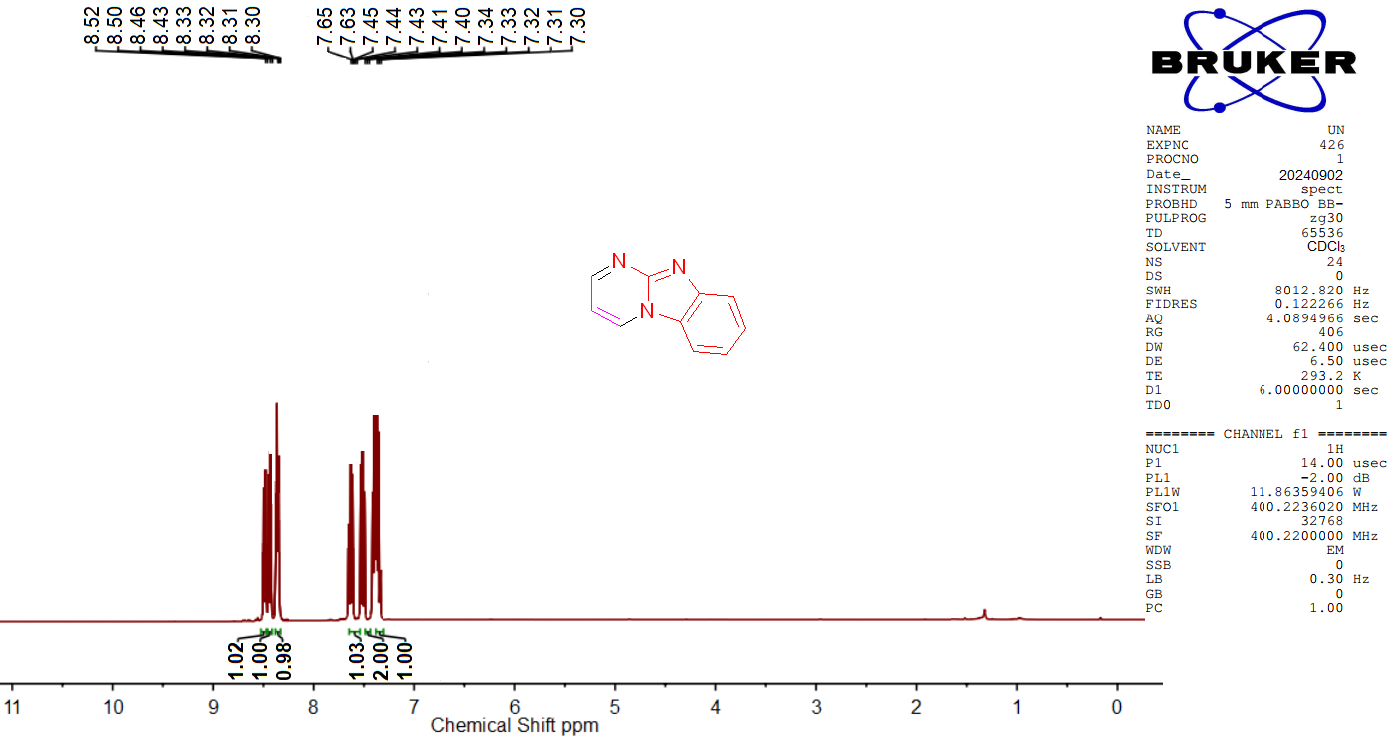


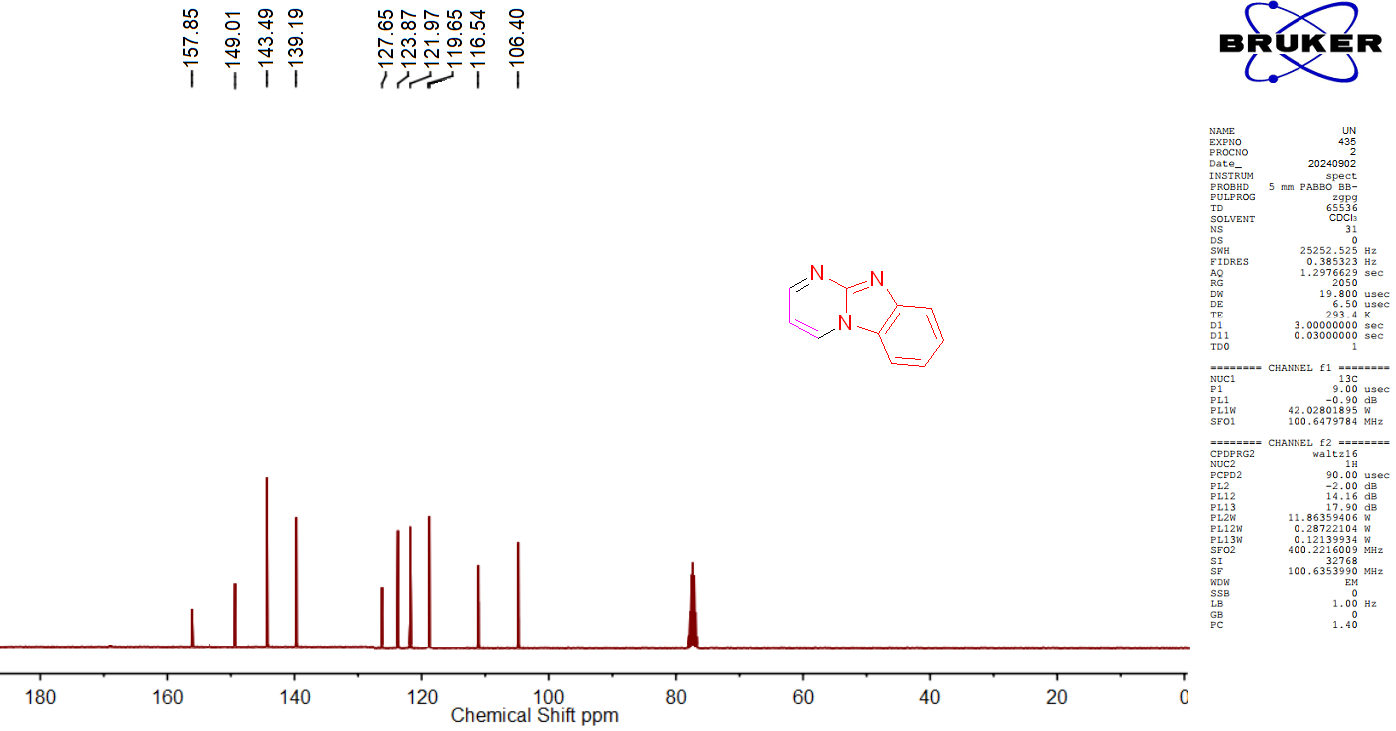


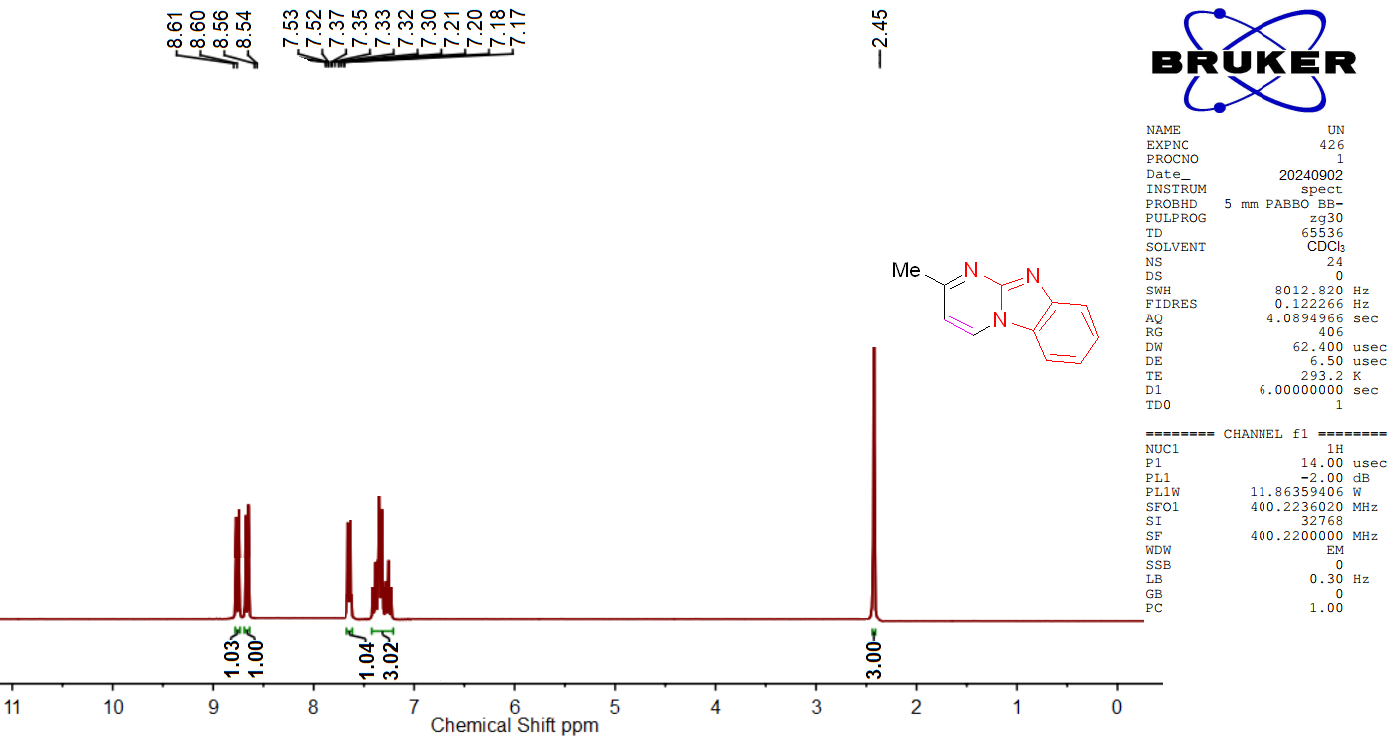


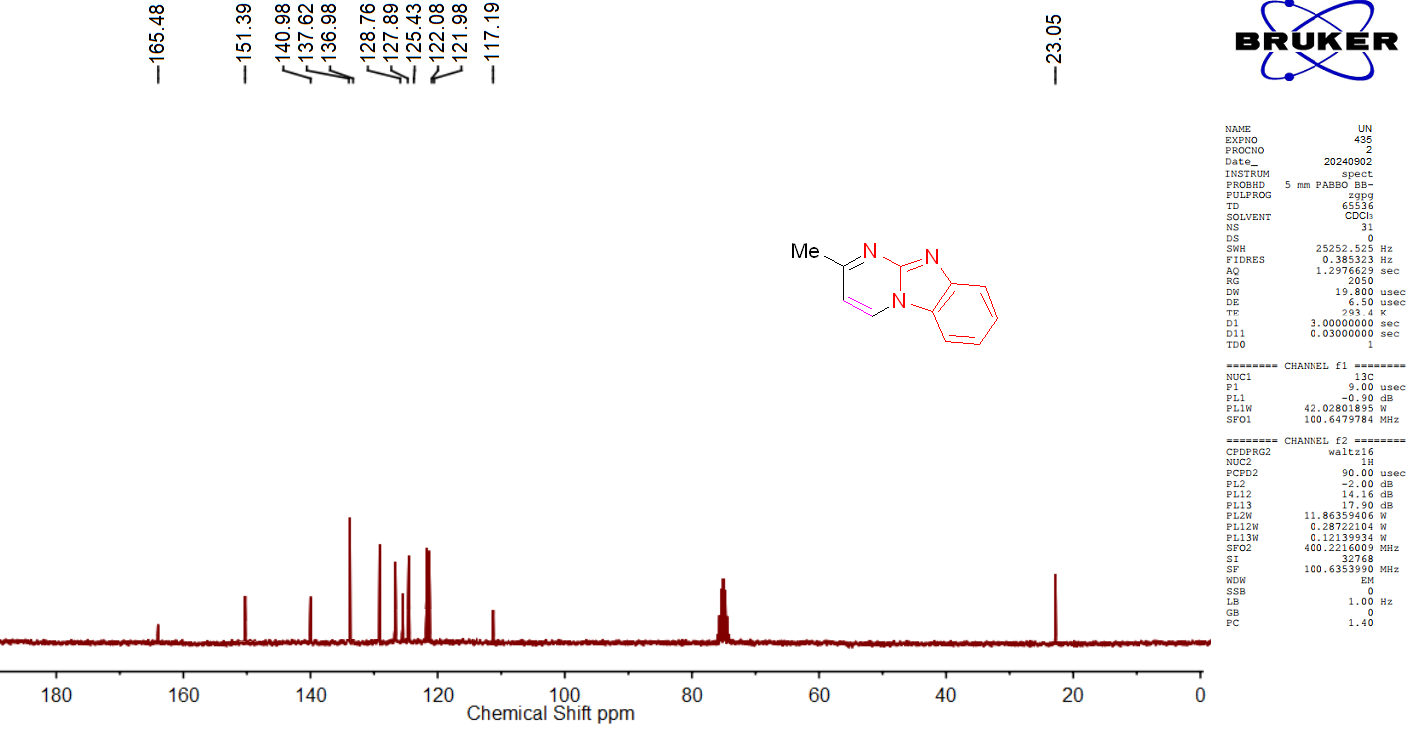


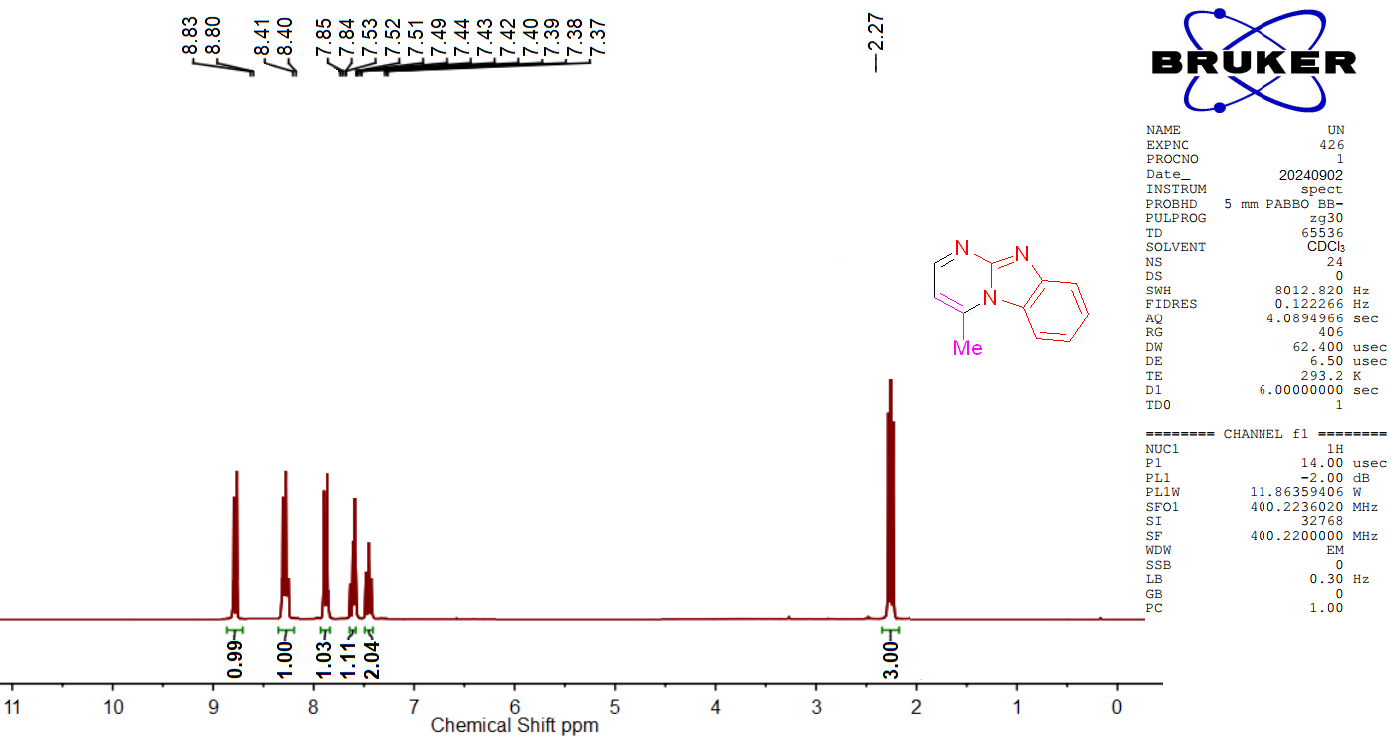


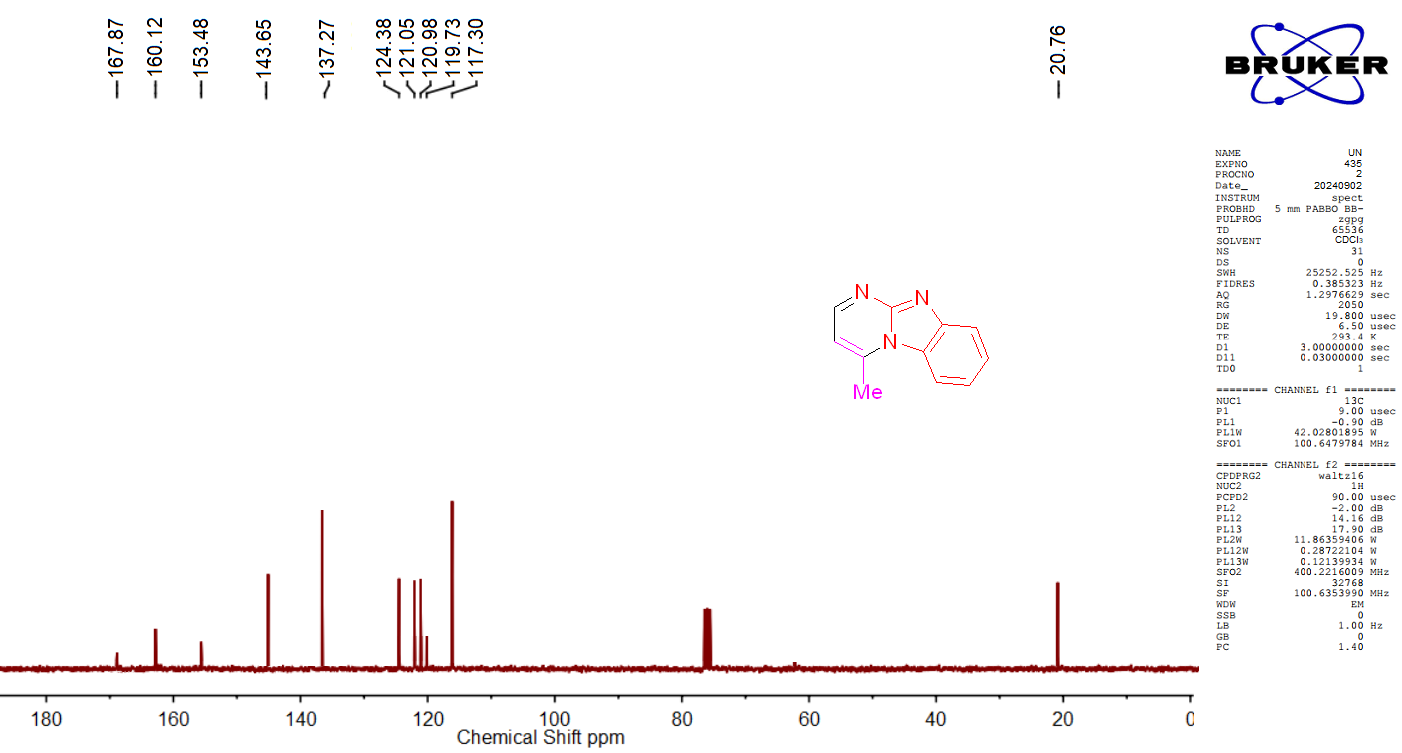


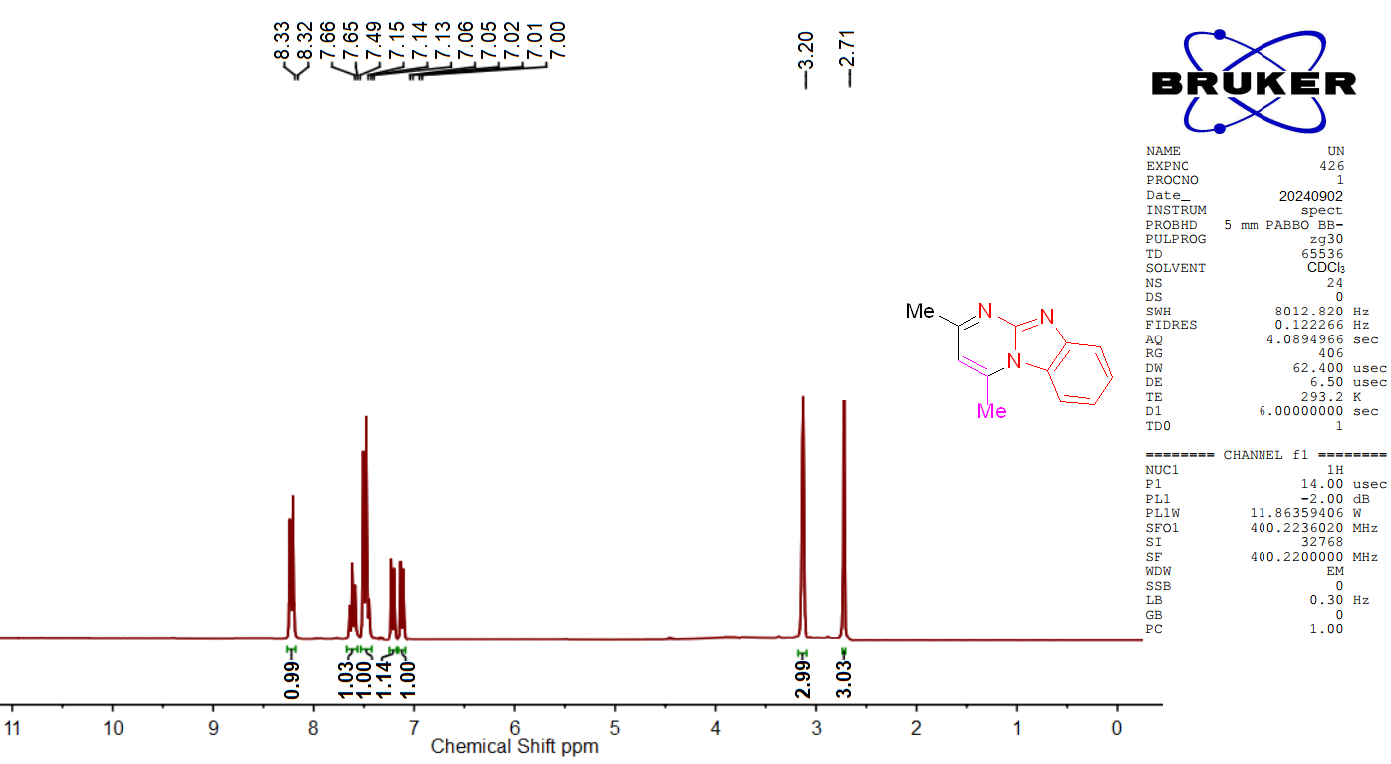


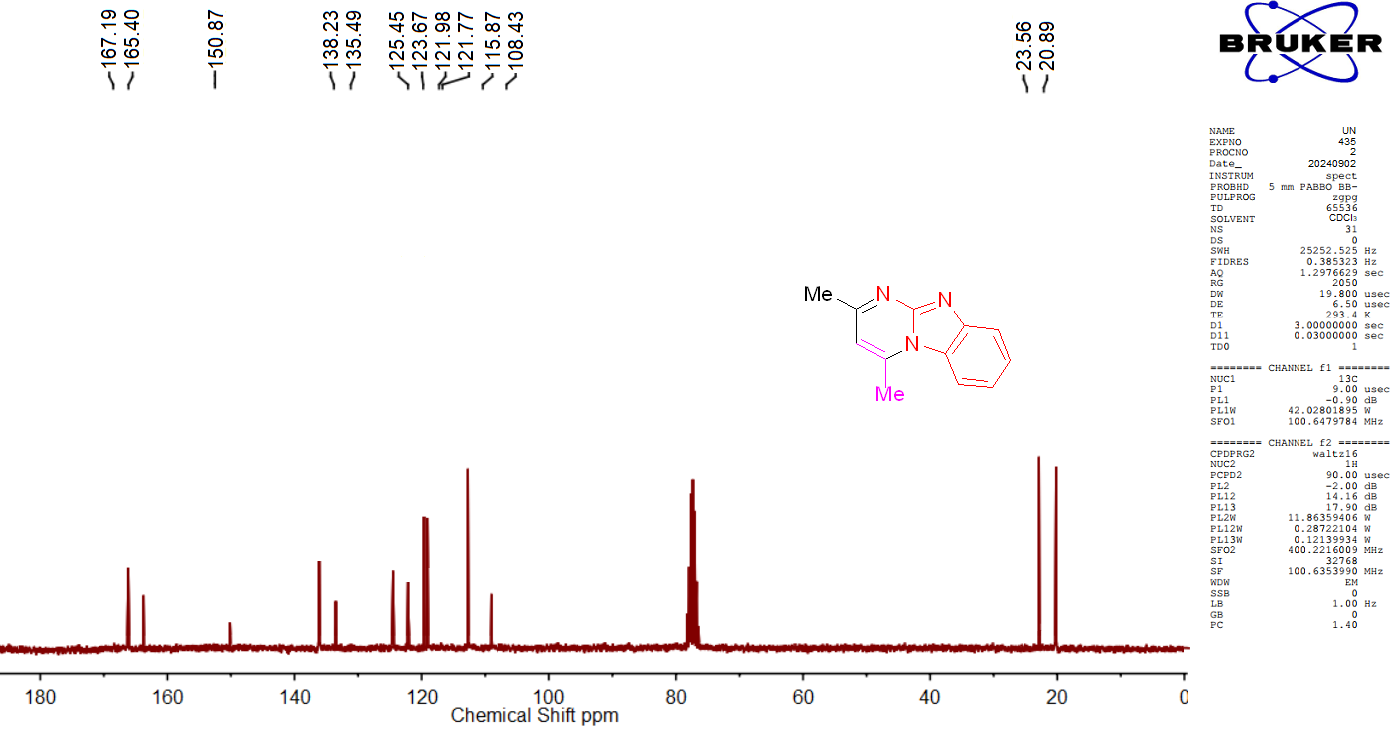


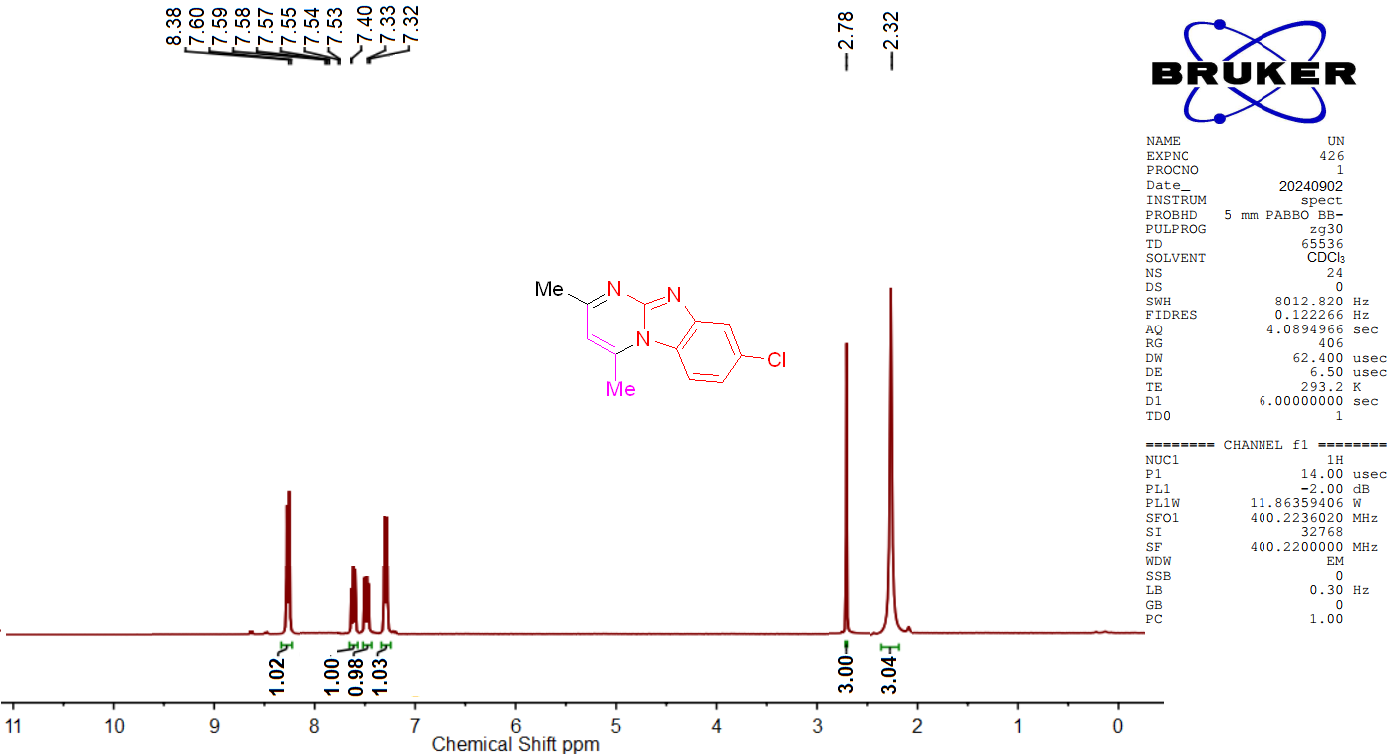


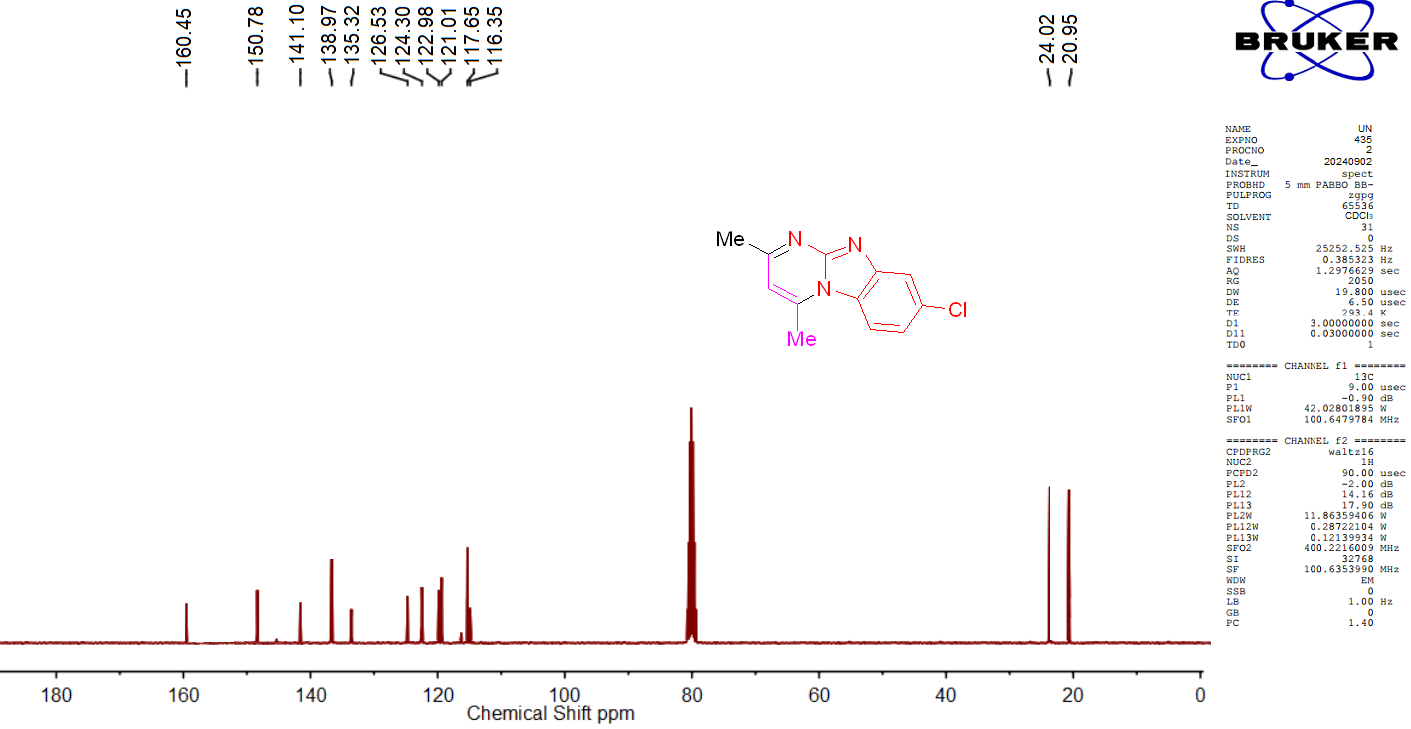


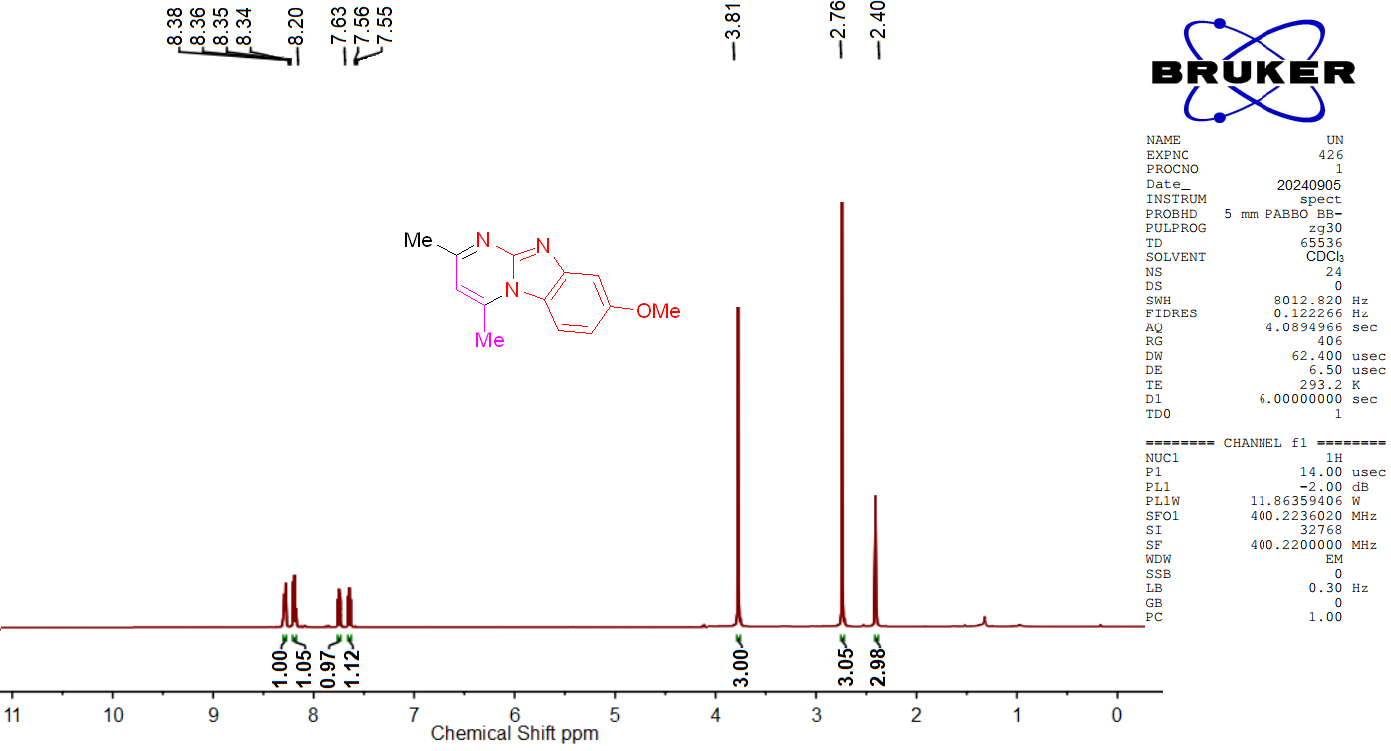


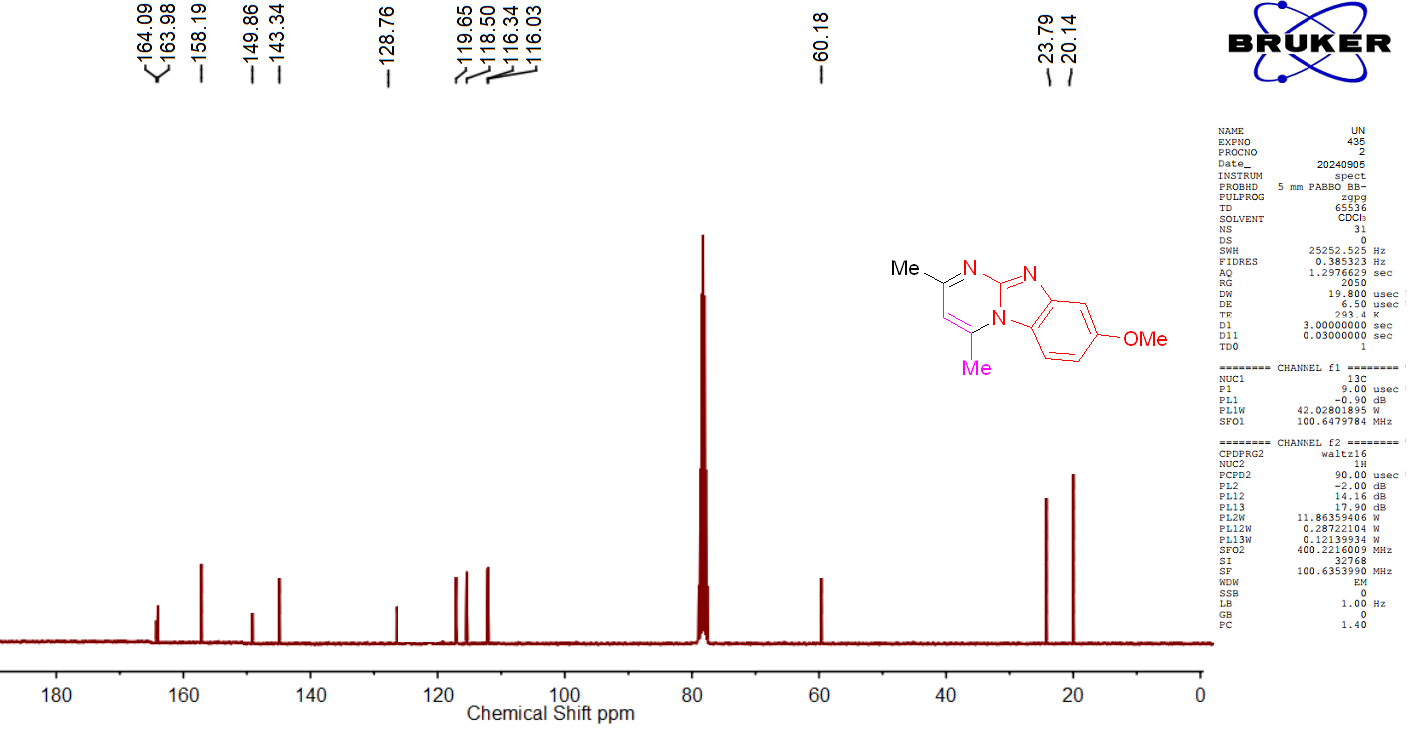


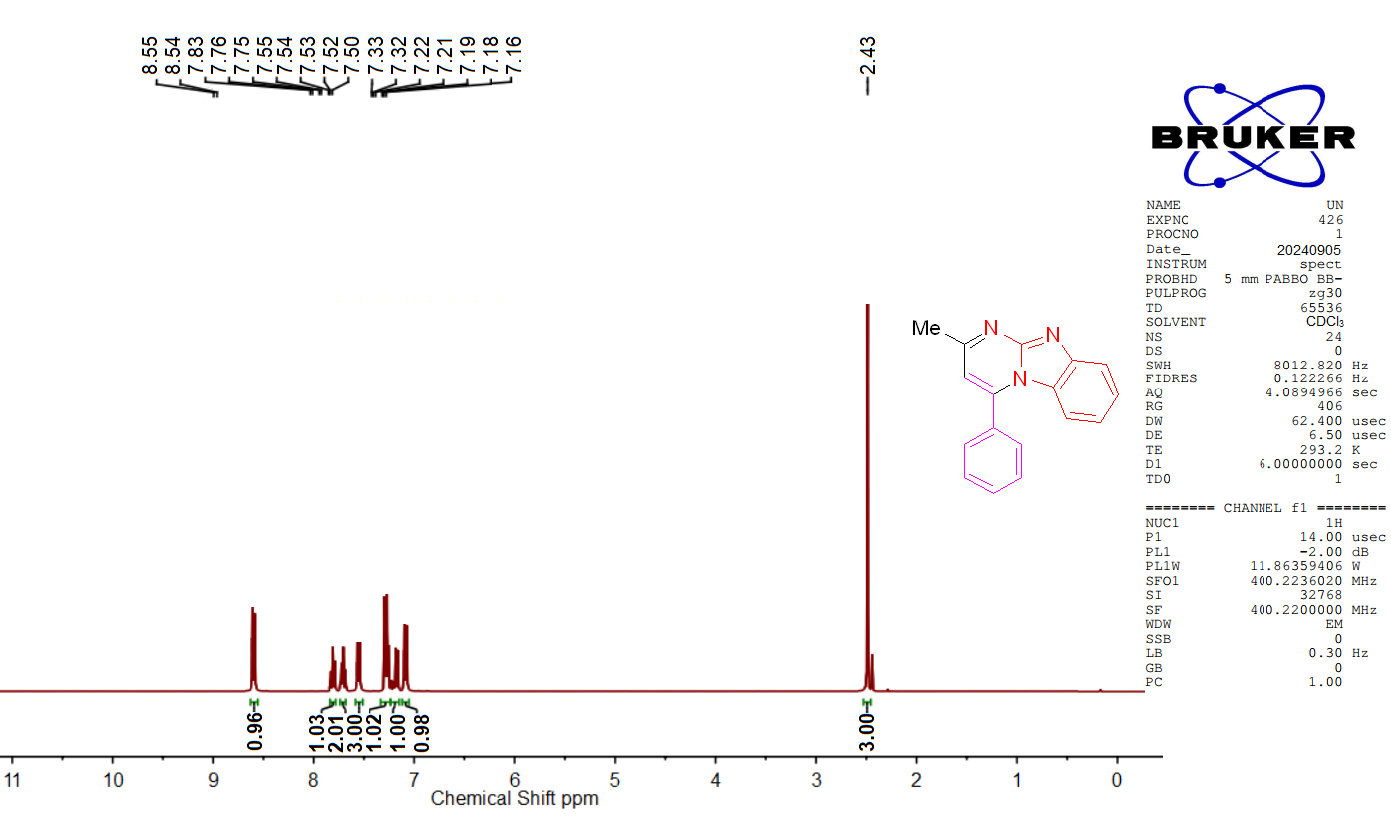


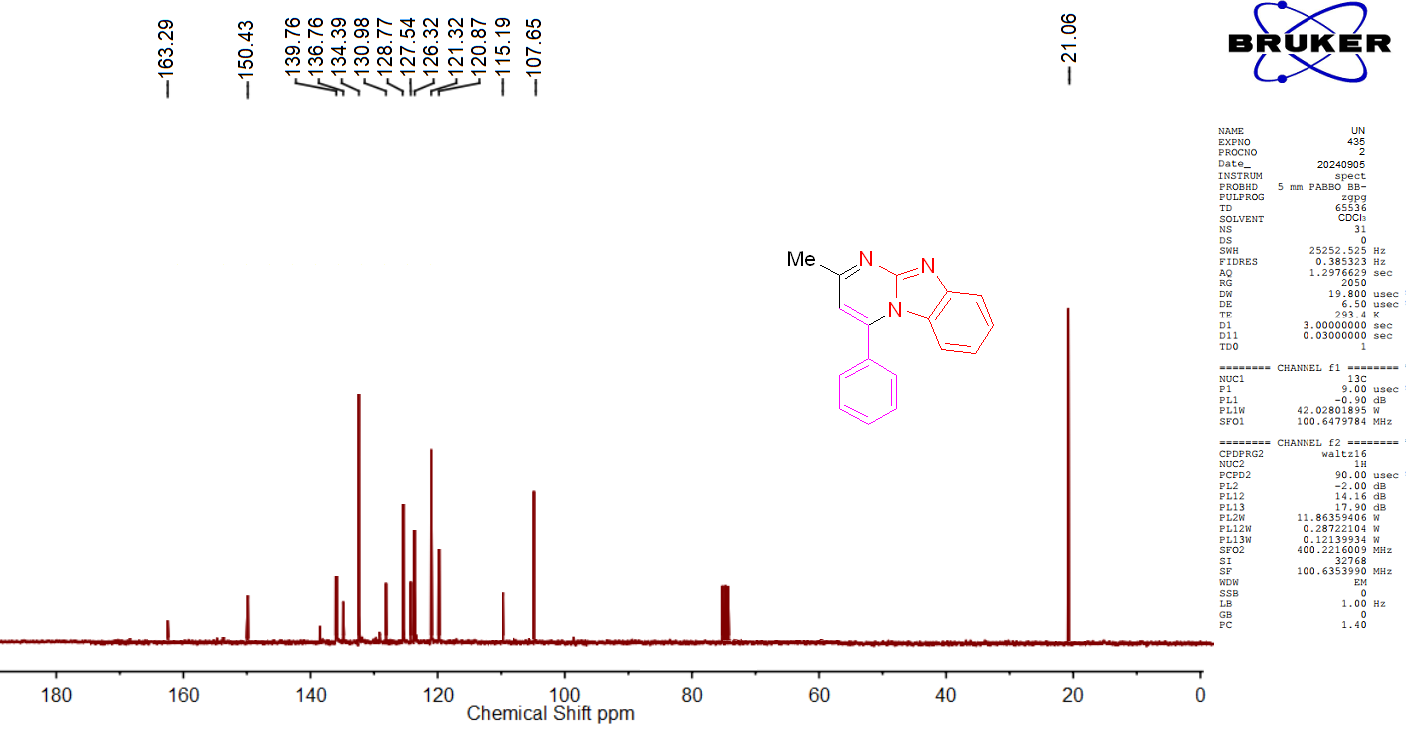


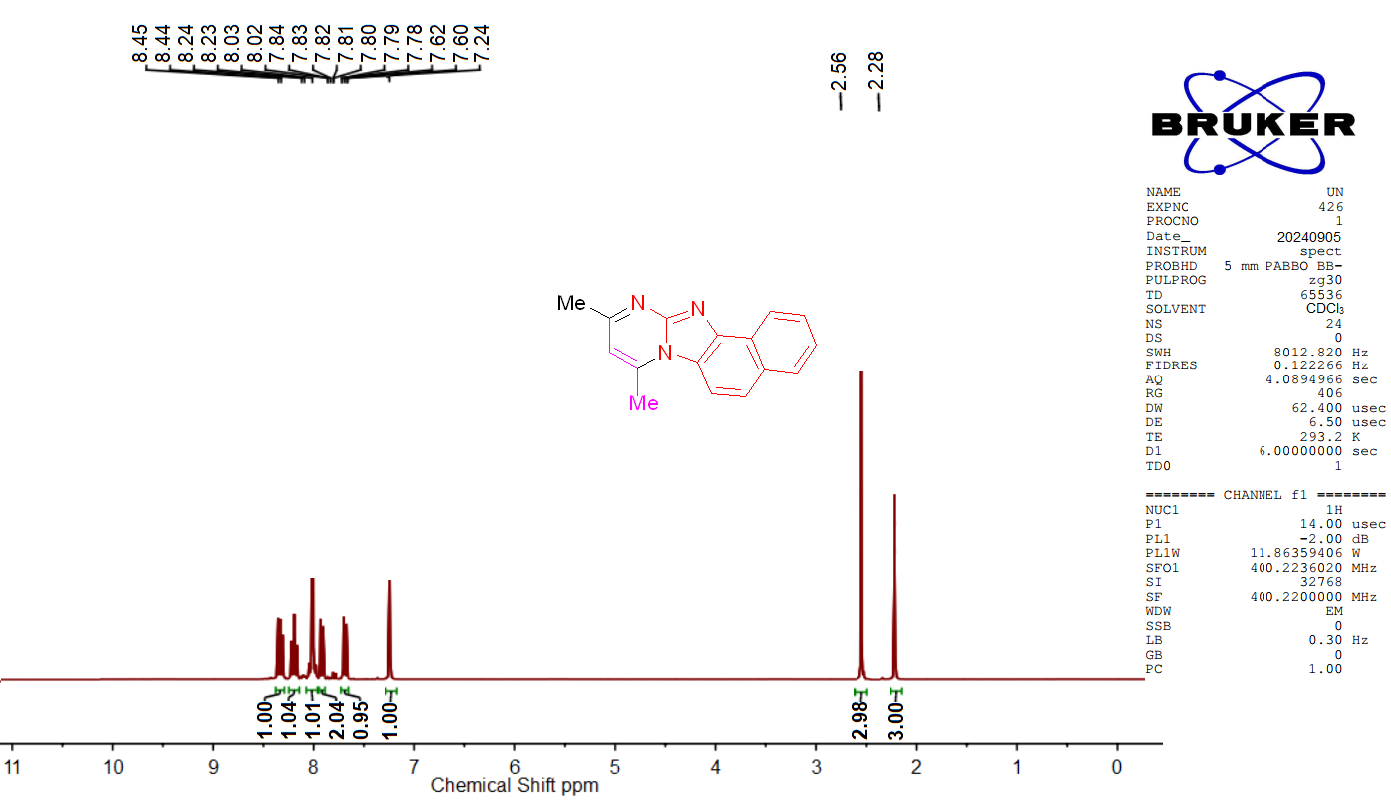


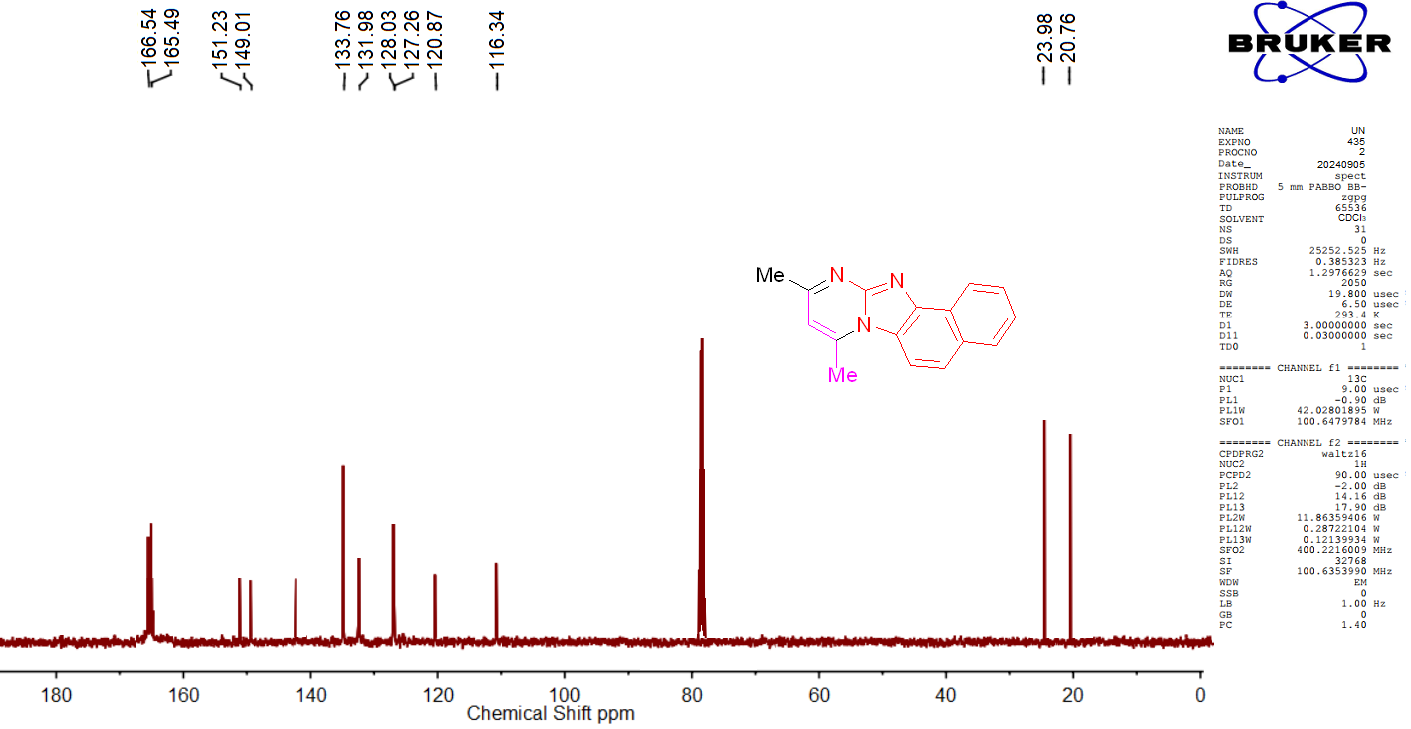


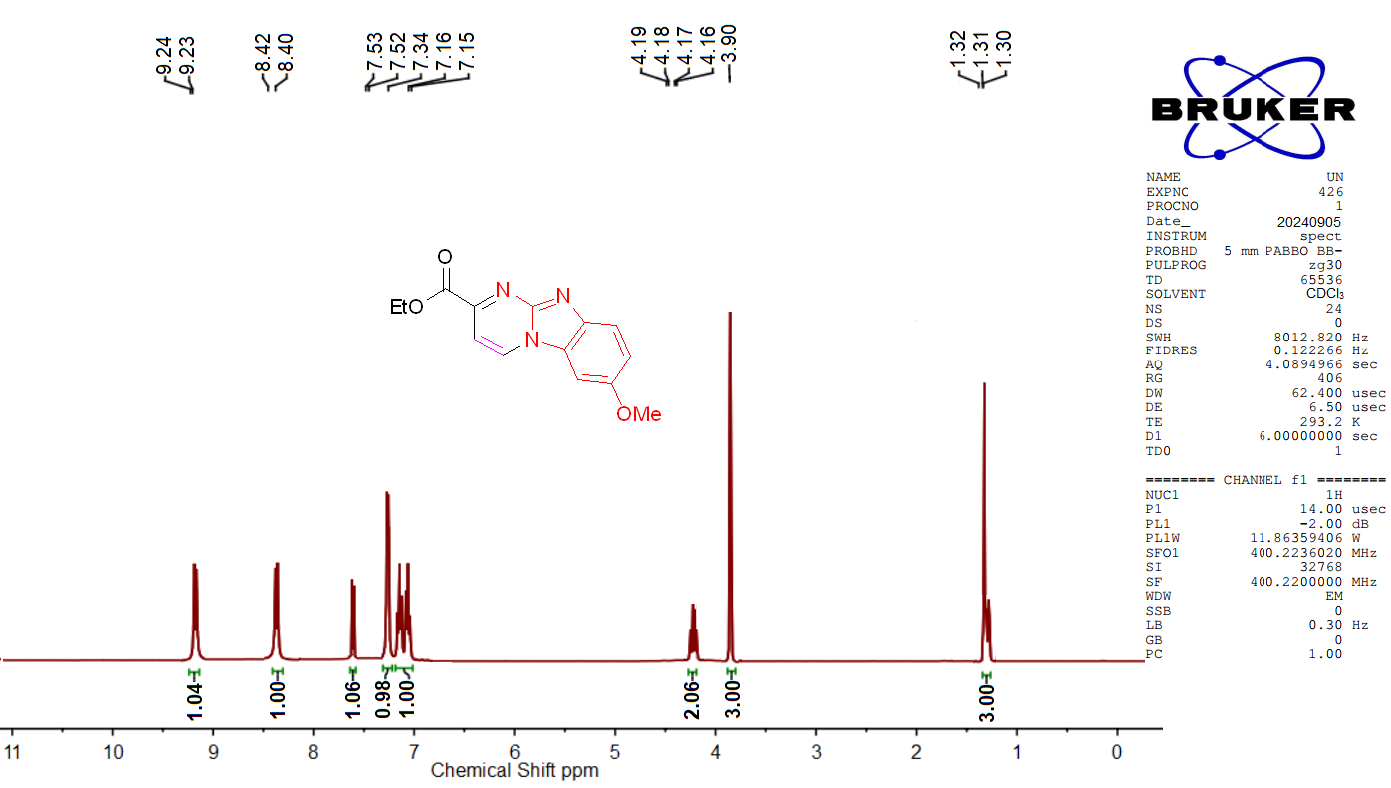


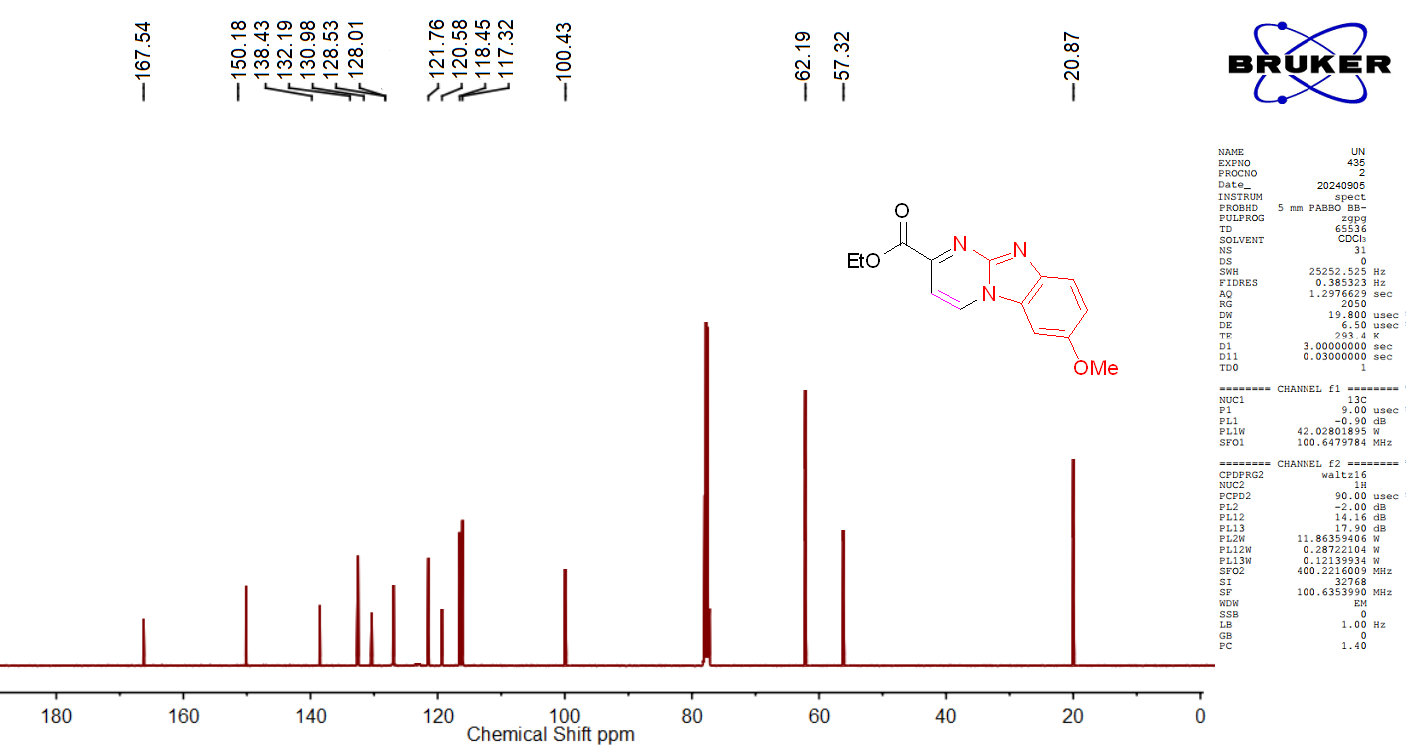


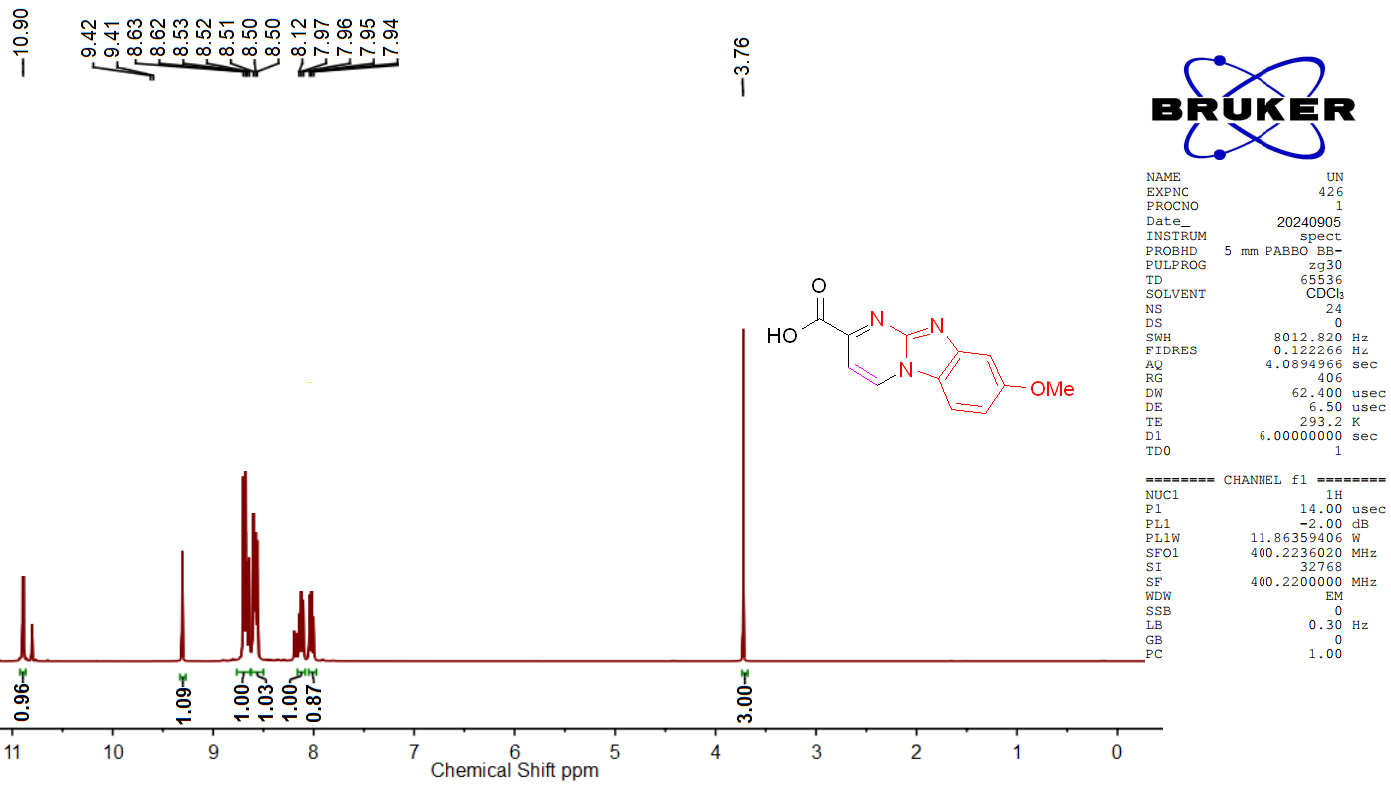


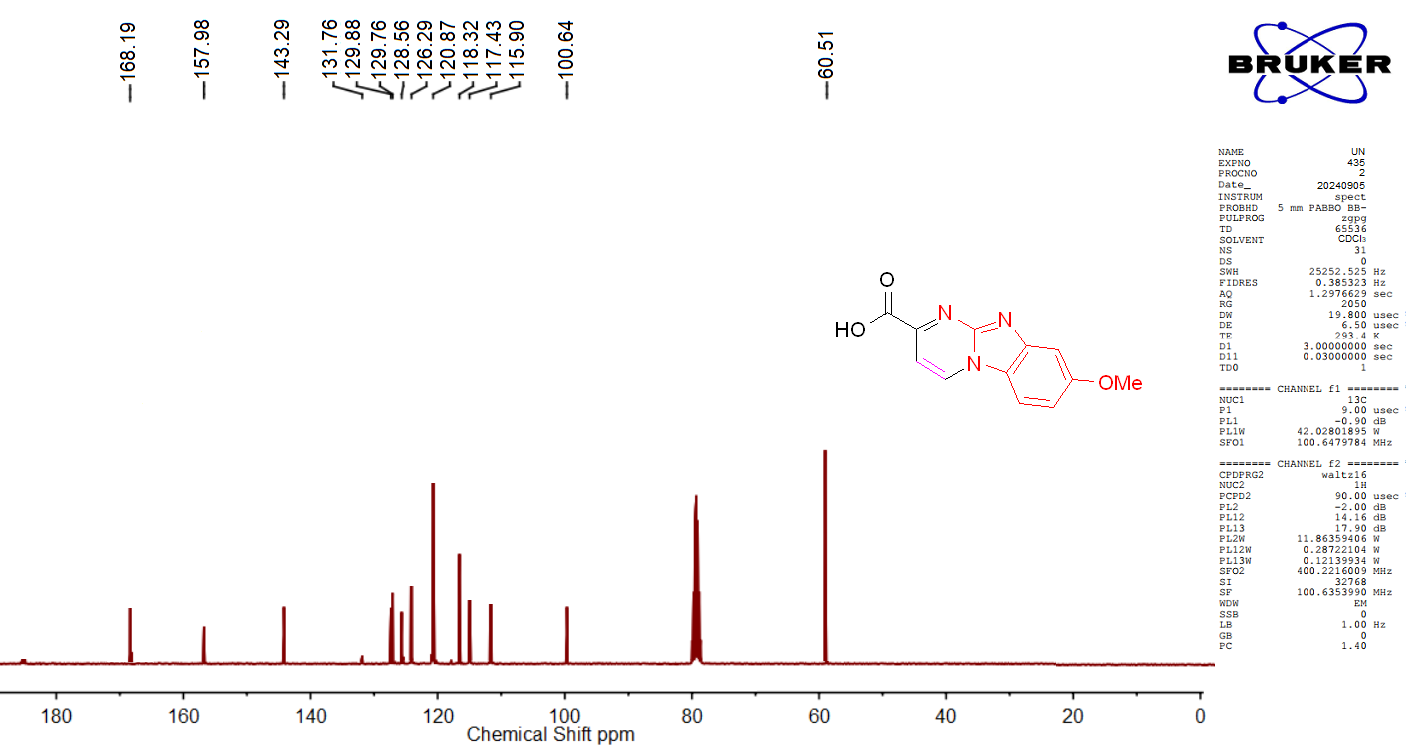


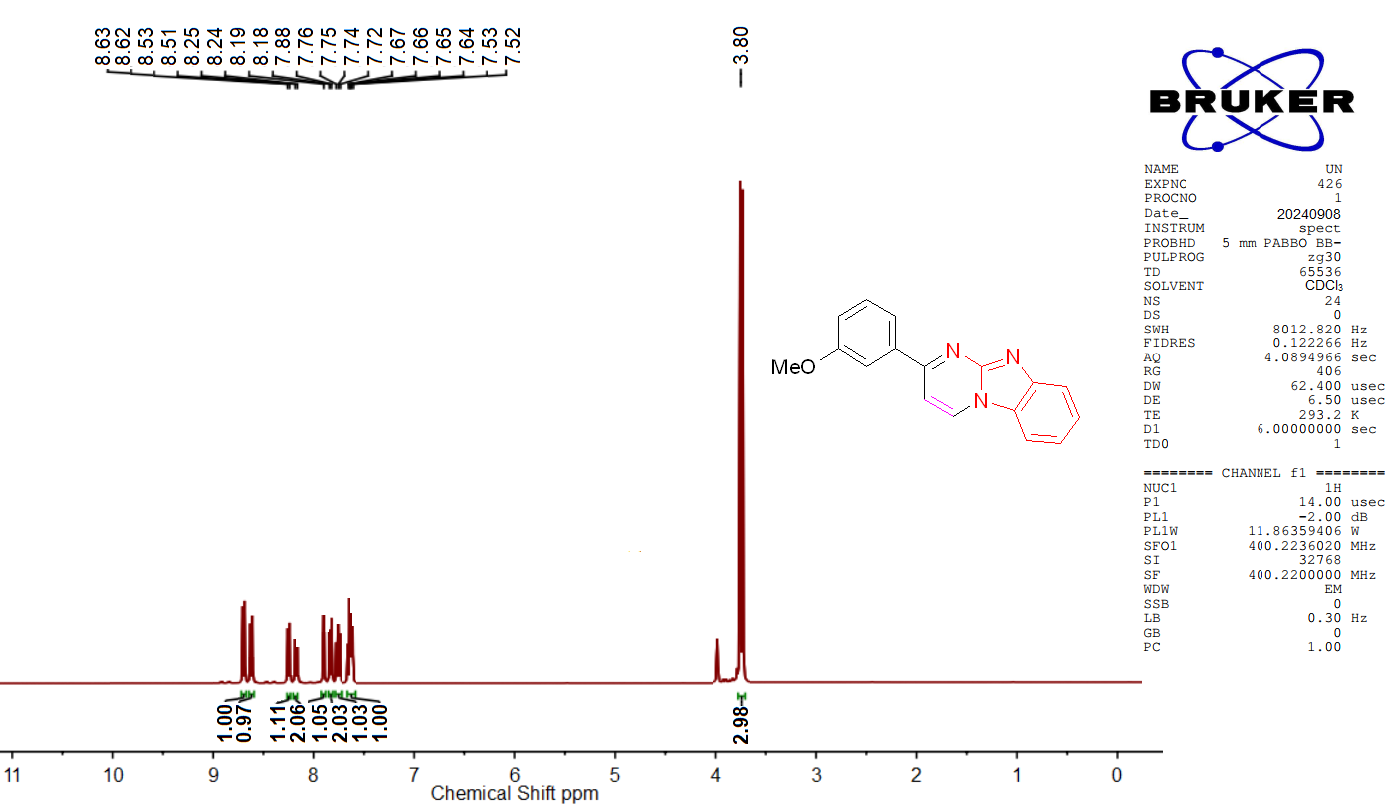


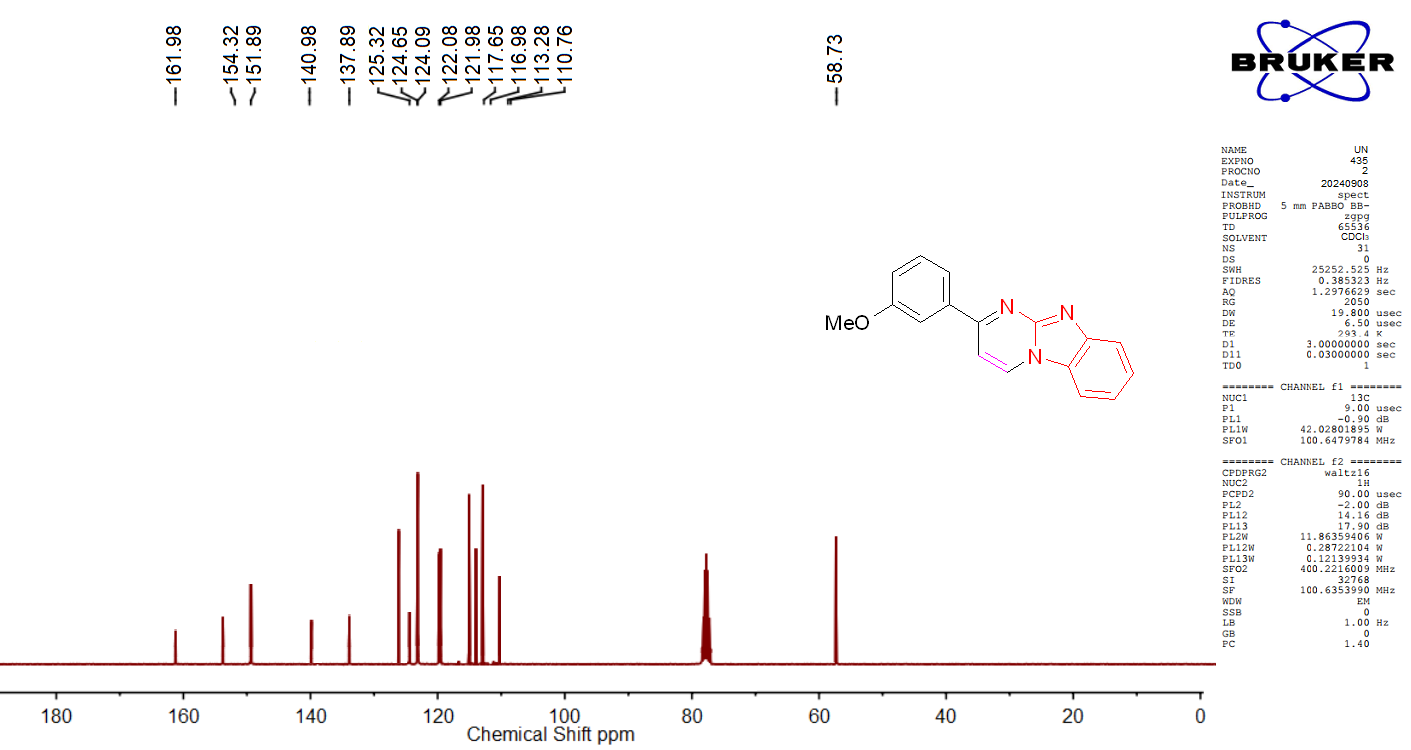


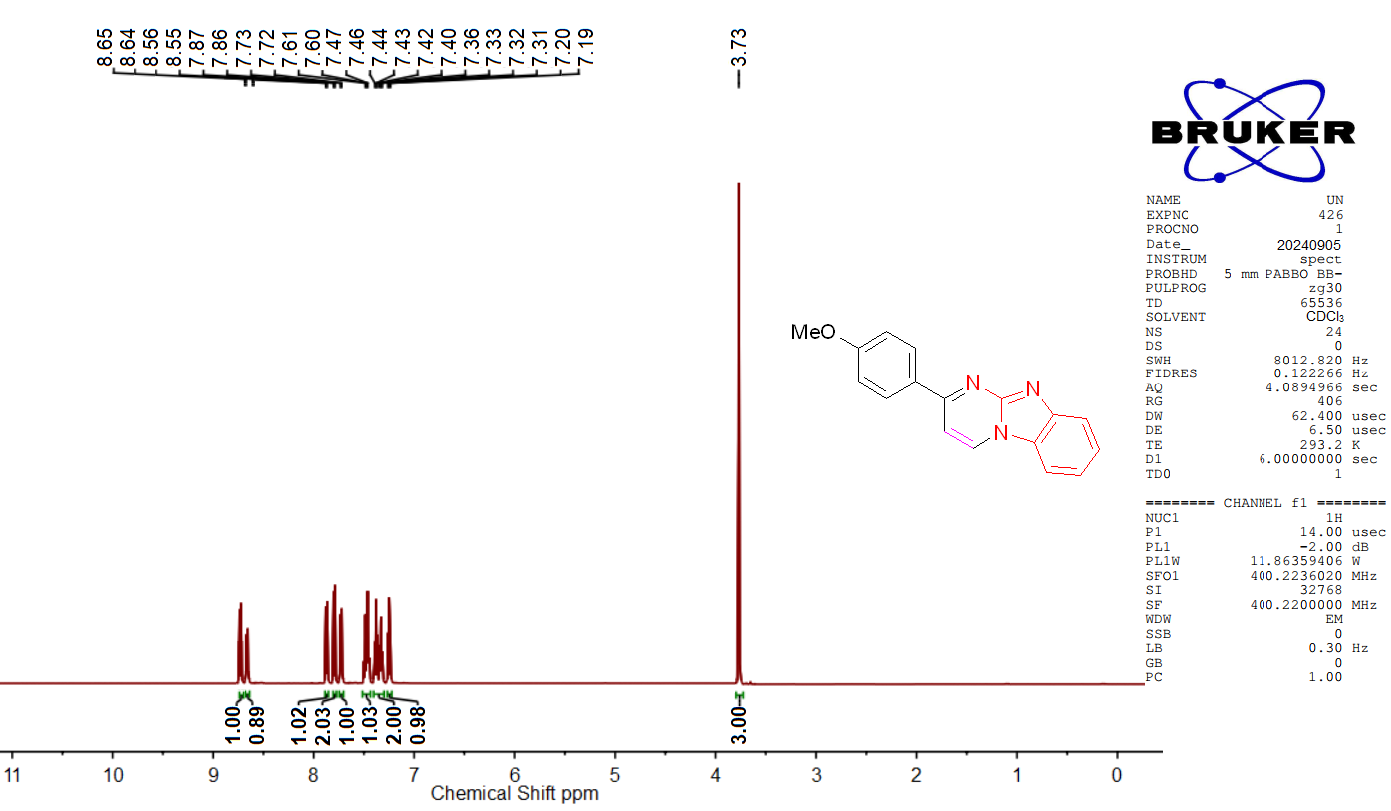


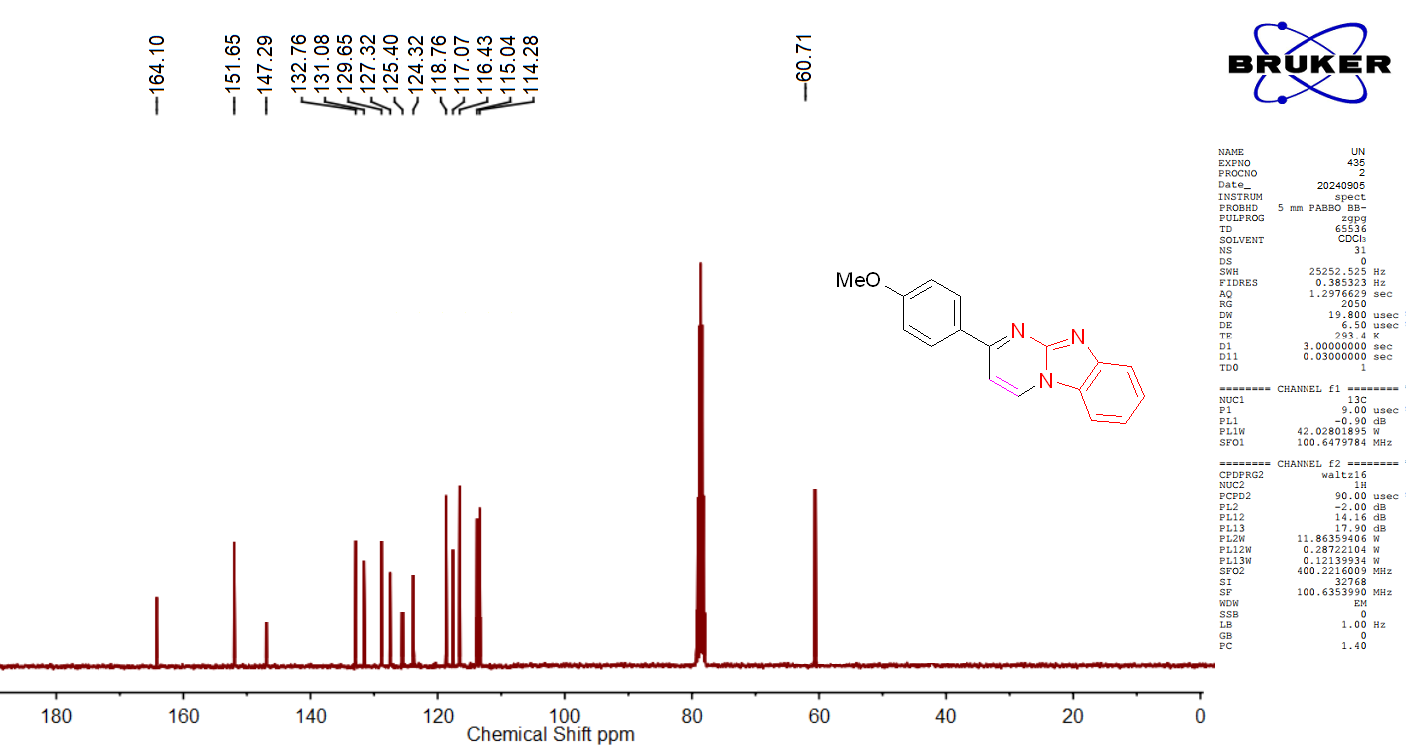


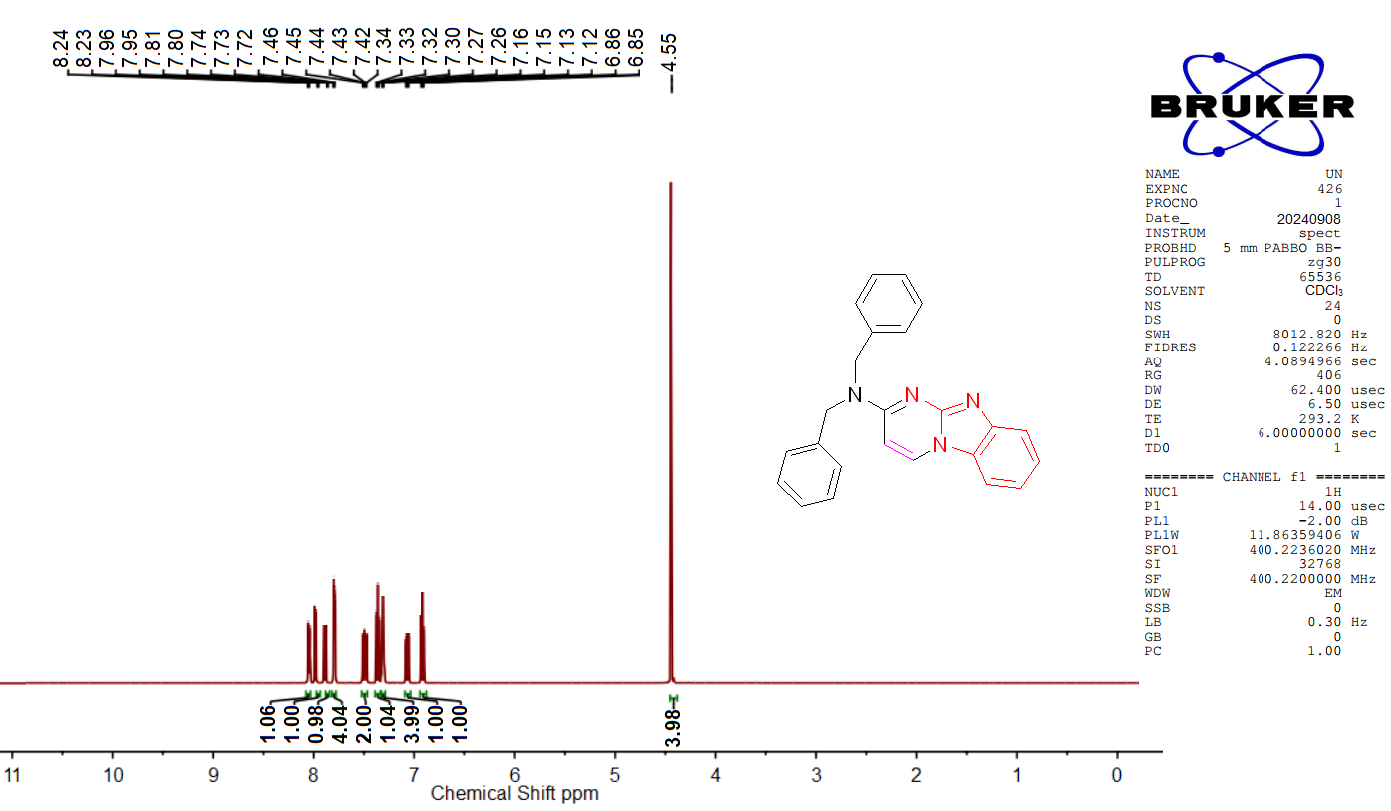


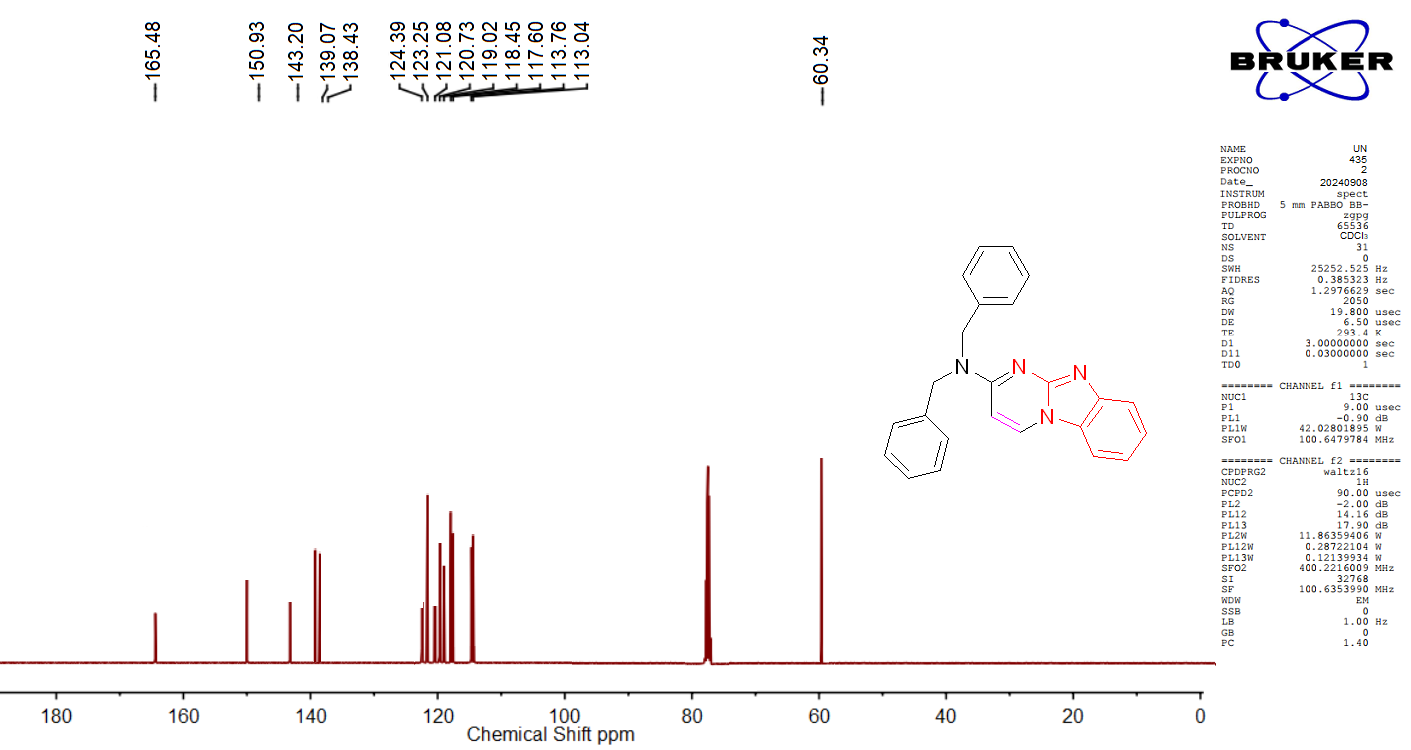


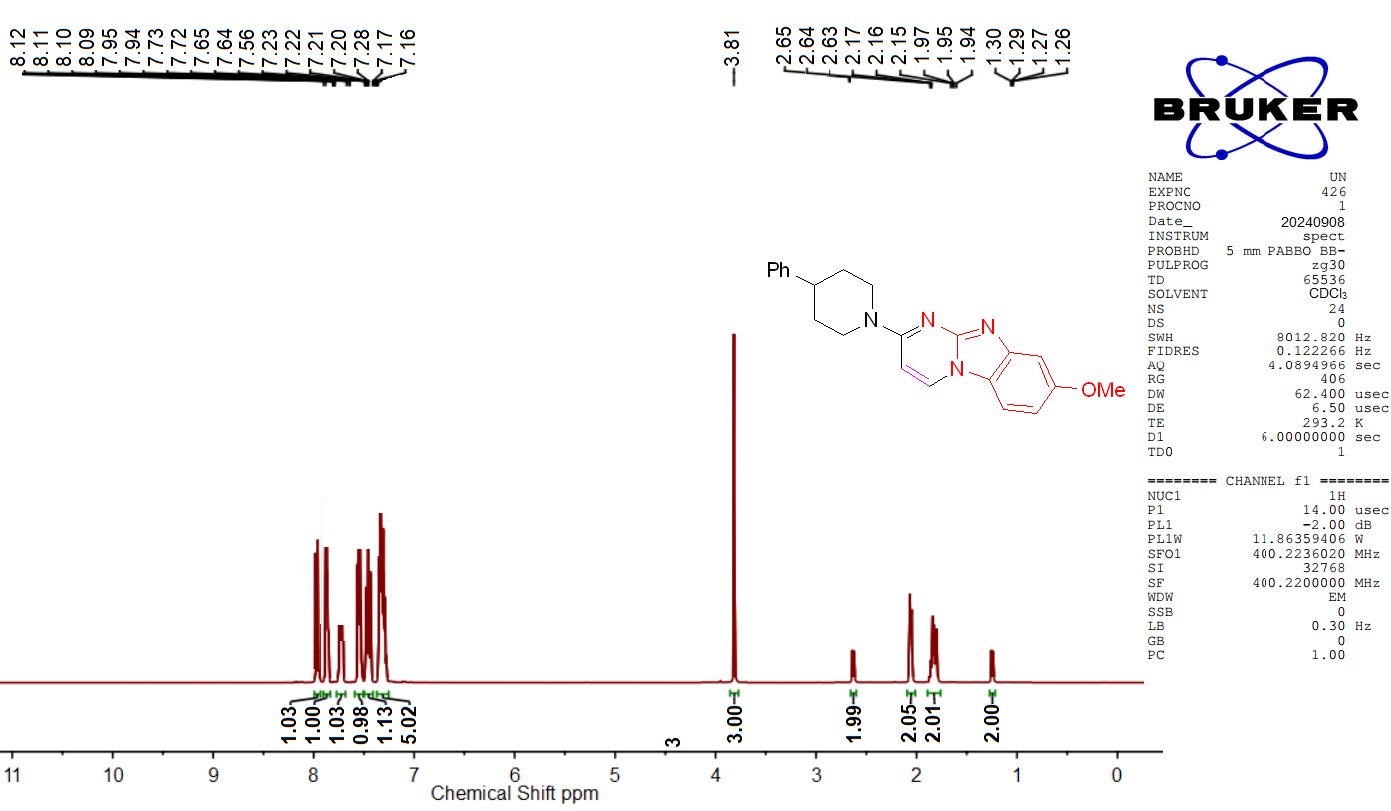


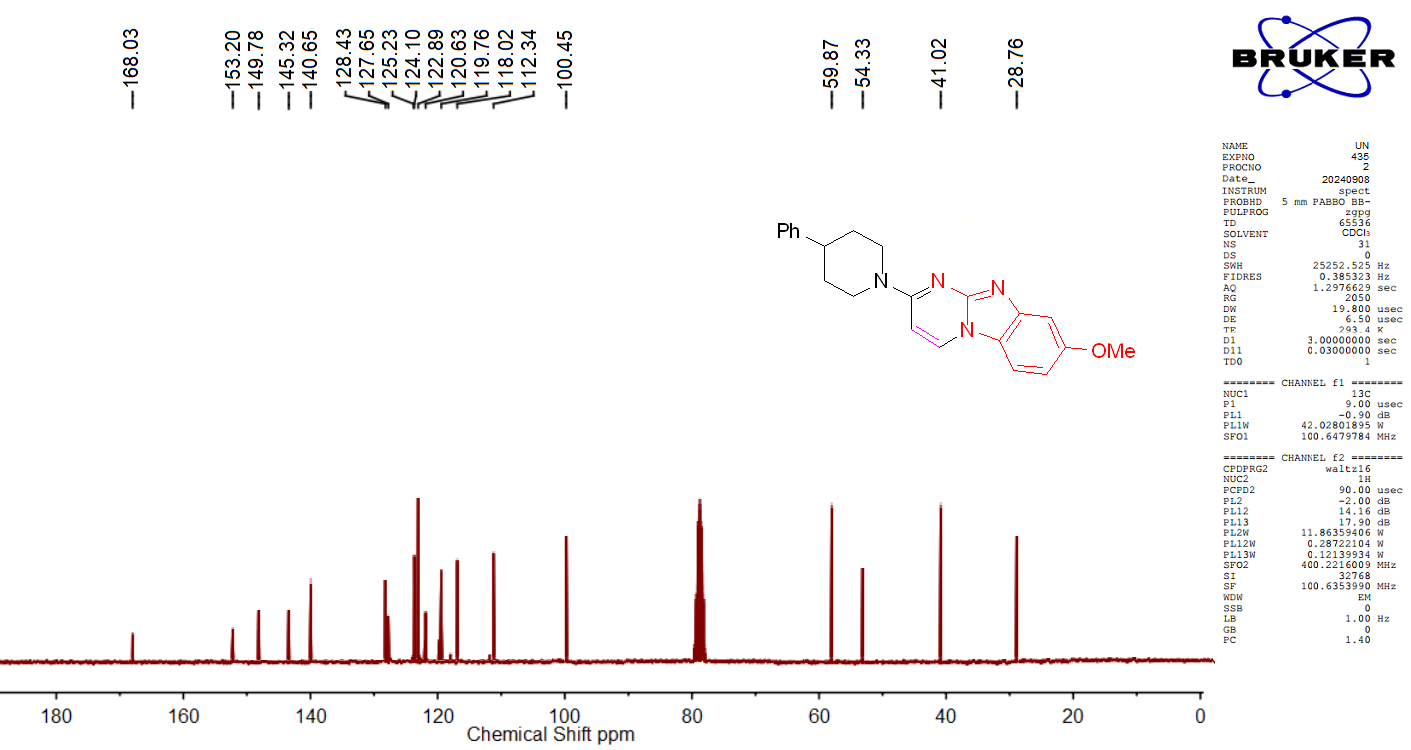


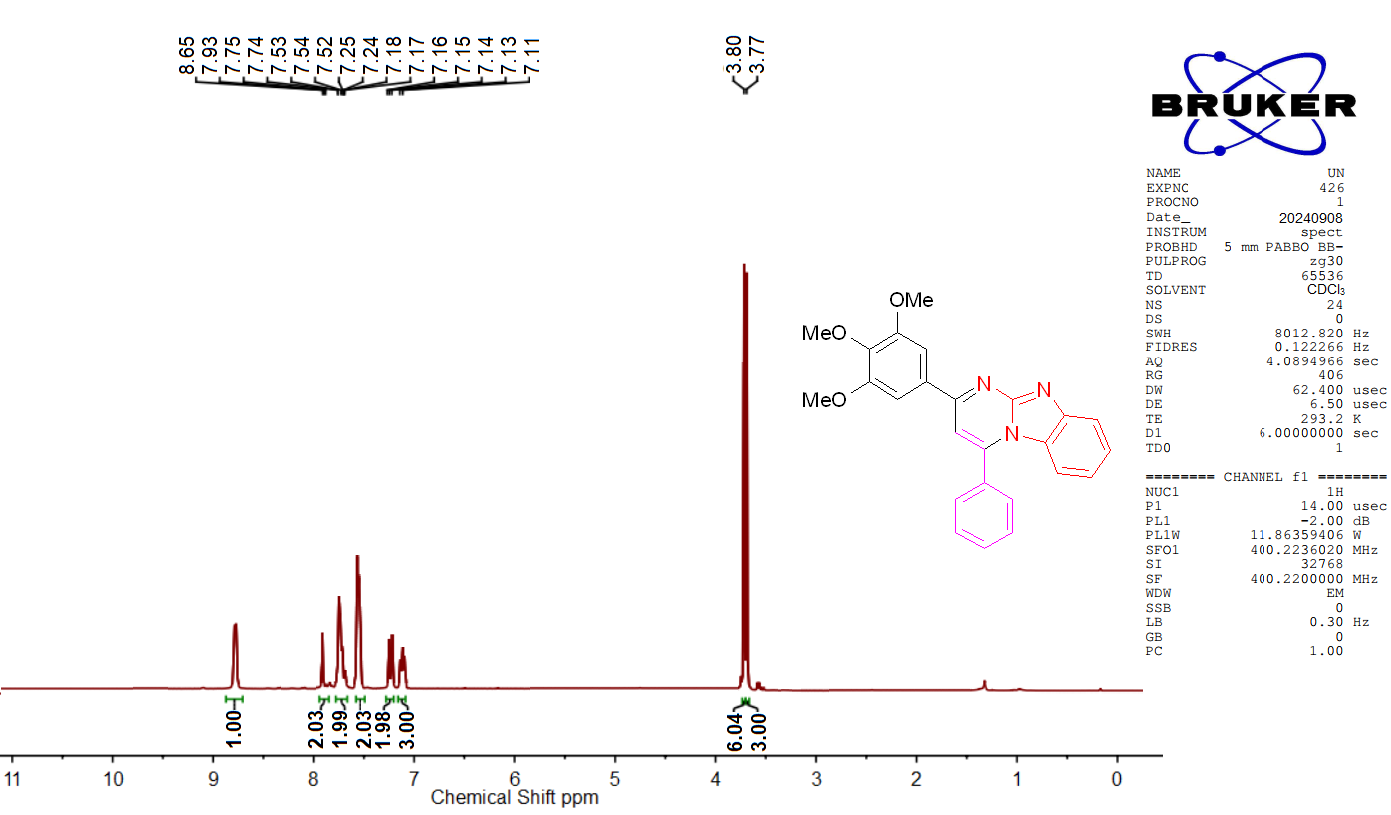


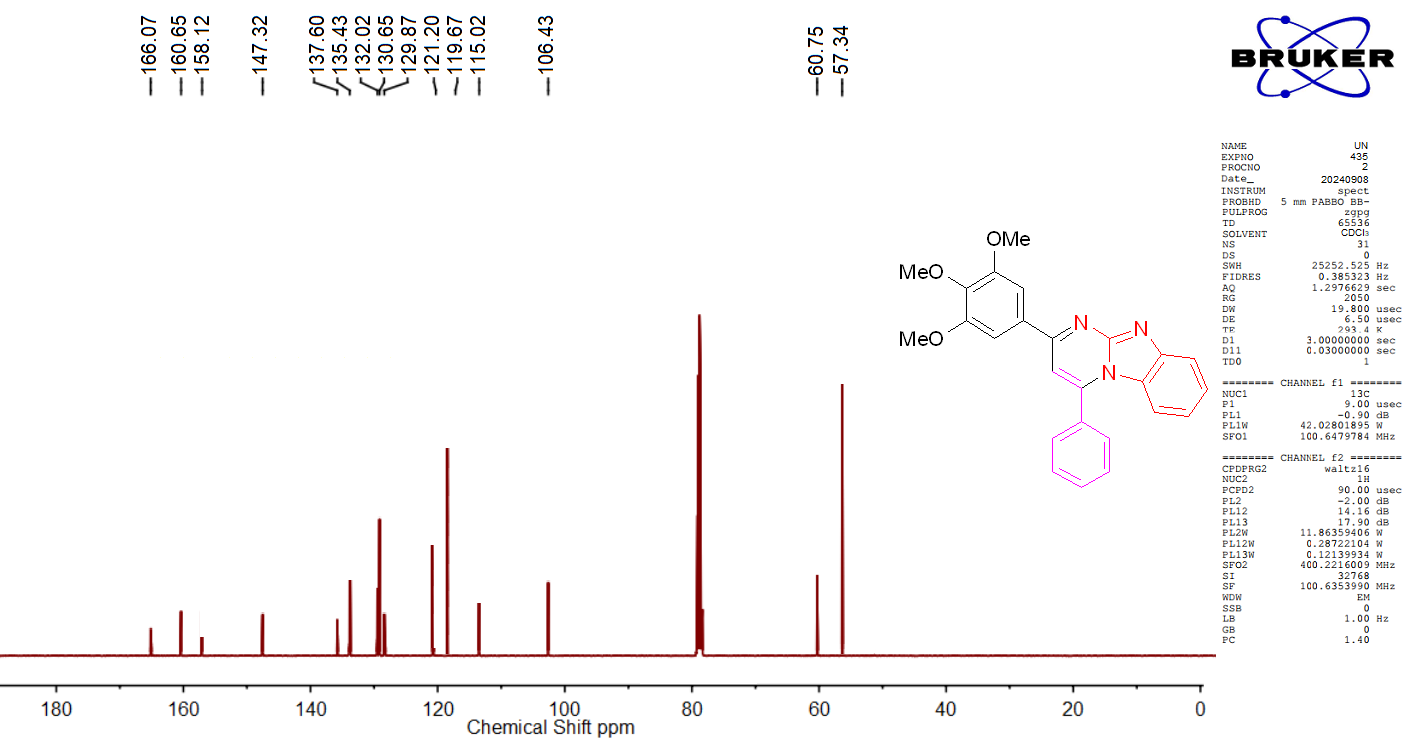


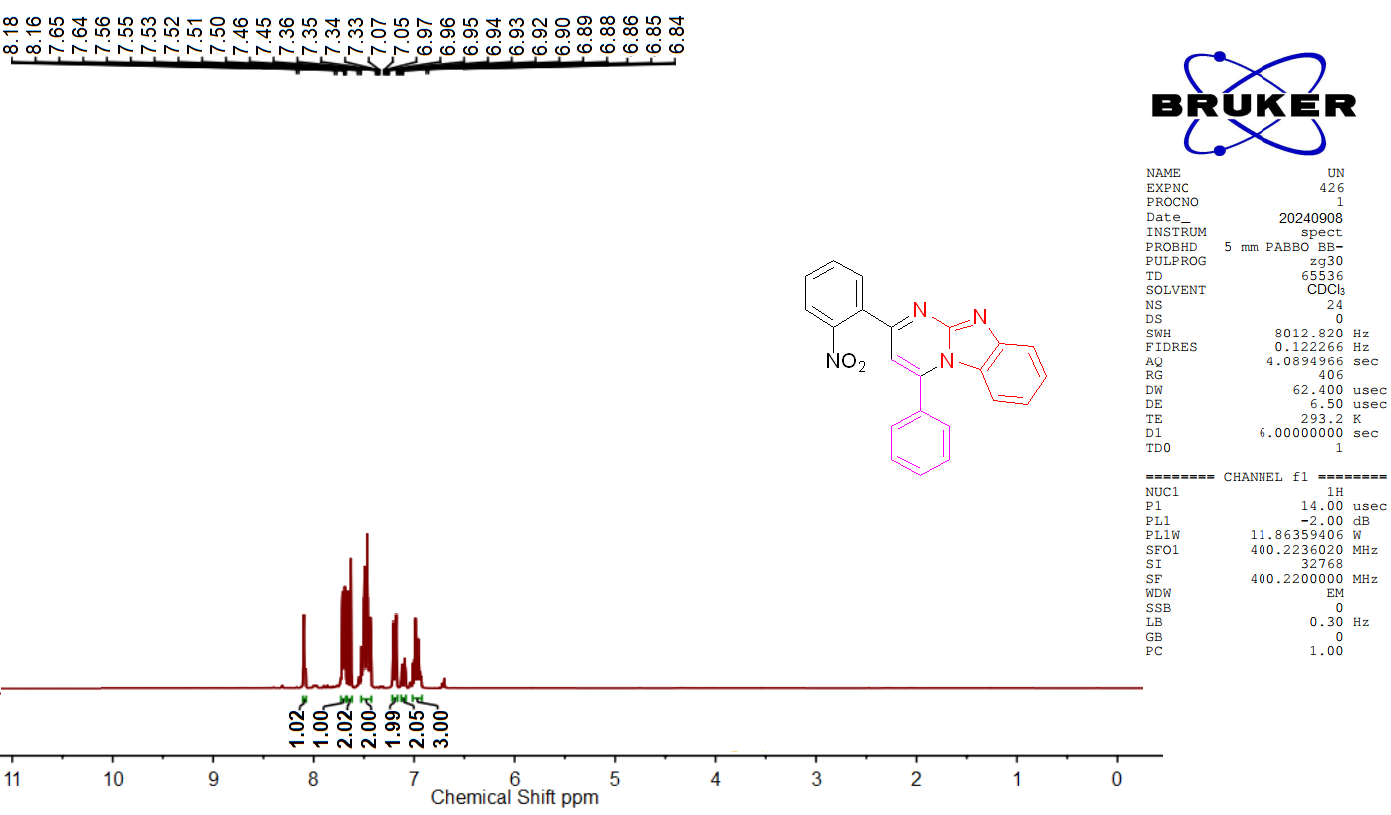


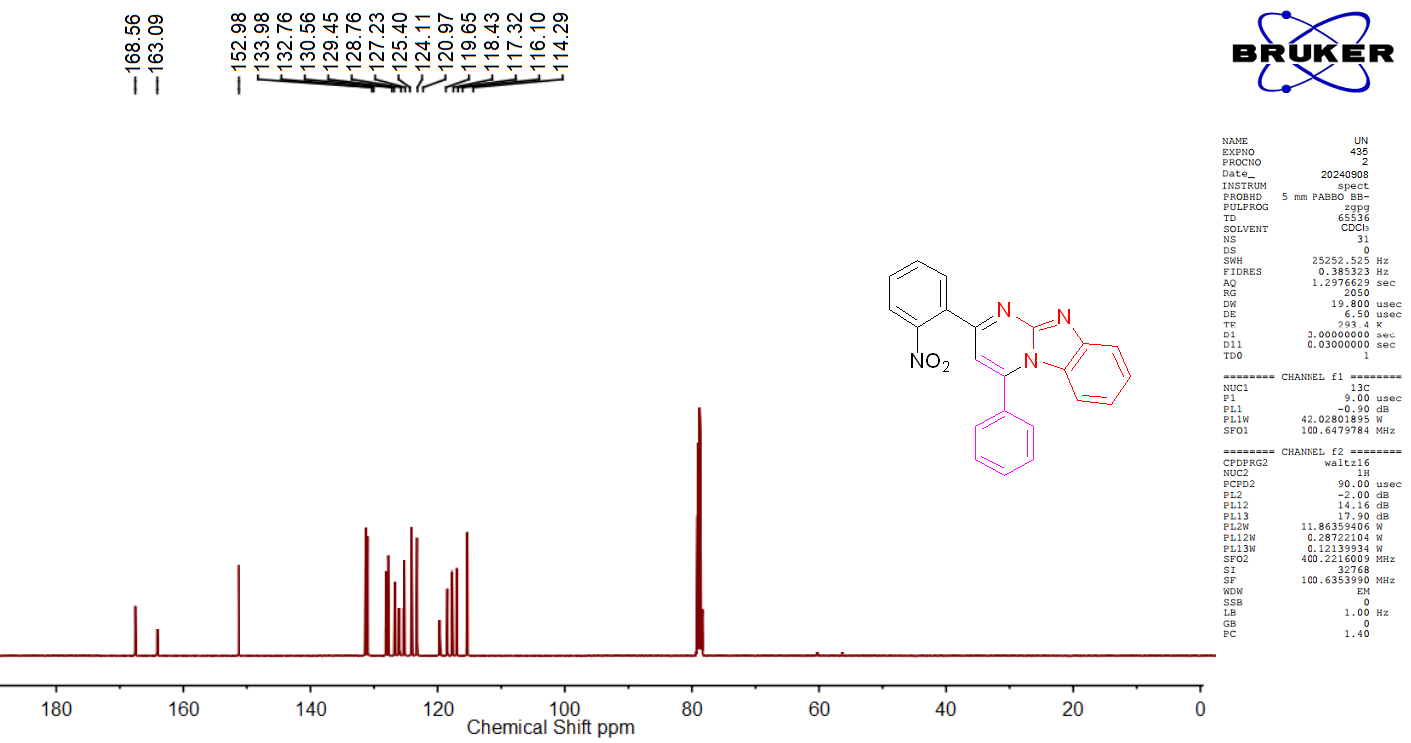


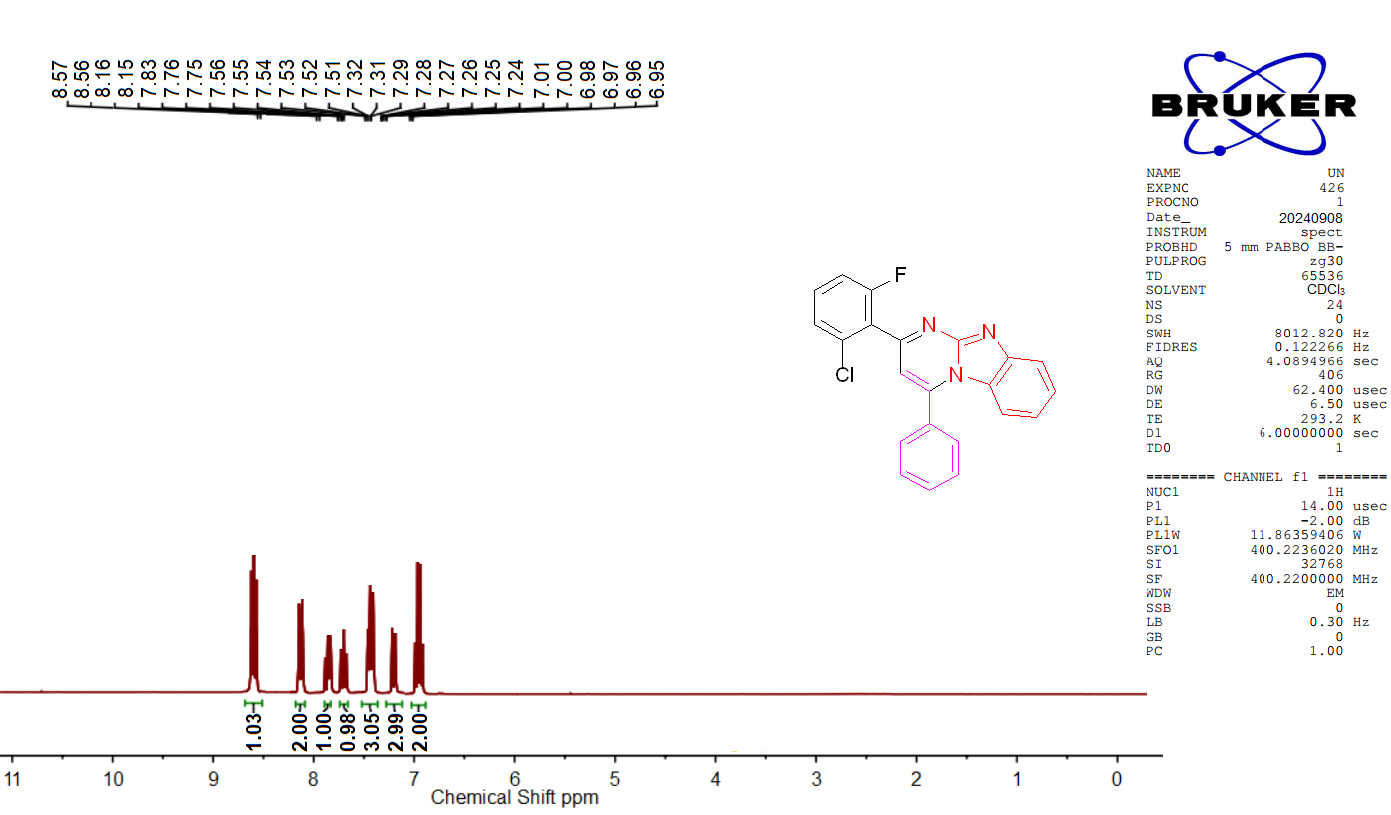


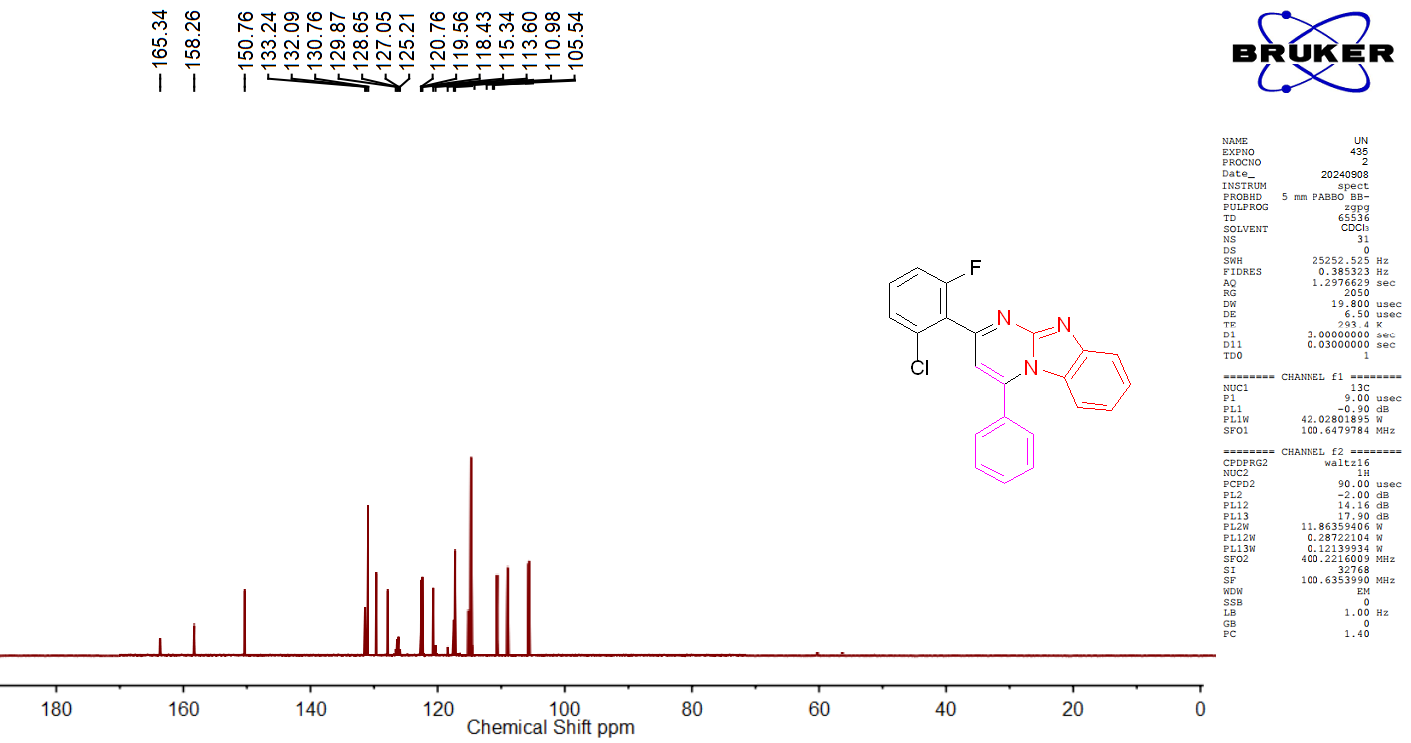

Supplement: Supplementary file 1 [file DataSheet1.docx]
